# A phase I, first-in-human study to evaluate the safety and tolerability, pharmacokinetics, and pharmacodynamics of MRG-001 in healthy subjects

**DOI:** 10.1016/j.xcrm.2023.101169

**Published:** 2023-08-25

**Authors:** Ali R. Ahmadi, George Atiee, Bart Chapman, Laurie Reynolds, John Sun, Andrew M. Cameron, Russell N. Wesson, James F. Burdick, Zhaoli Sun

**Affiliations:** 1 MedRegen LLC, Baltimore, MD, USA; 2 ICON PLC, San Antonio, TX, USA; 3 Department of Surgery, Johns Hopkins School of Medicine, Baltimore, MD 21205, USA

**Keywords:** clinical trial, phase I, healthy volunteers, safety, MRG-001, stem cells, plerixafor, tacrolimus, pharmacokinetics, pharmacodynamics

## Abstract

Preclinical studies demonstrate that pharmacological mobilization and recruitment of endogenous bone marrow stem cells and immunoregulatory cells by a fixed-dose drug combination (MRG-001) improves wound healing, promotes tissue regeneration, and prevents allograft rejection. In this phase I, first-in-human study, three cohorts receive subcutaneous MRG-001 or placebo, every other day for 5 days. The primary outcome is safety and tolerability of MRG-001. Fourteen subjects received MRG-001 and seven received a placebo. MRG-001 is safe over the selected dose range. There are no clinically significant laboratory changes. The intermediate dose group demonstrates the most significant white blood cell, stem cell, and immunoregulatory cell mobilization. PBMC RNA sequencing and gene set enrichment analysis reveal 31 down-regulated pathways in the intermediate MRG-001 dose group compared with no changes in the placebo group. MRG-001 is safe across all dose ranges. MRG-001 may be a clinically useful therapy for immunoregulation and tissue regeneration (ClinicalTrials.gov: NCT04646603).

## Highlights

• MRG-001 exhibits immunoregulatory and regenerative properties in animal models
• The phase I trial confirms the favorable safety profile for multiple-dose regimens
• Stem cells and immunoregulatory T cells are successfully mobilized by MRG-001
• MRG-001 down-regulates 31 pathways related to inflammation and allograft rejection

## Graphical abstract

**Figure:**
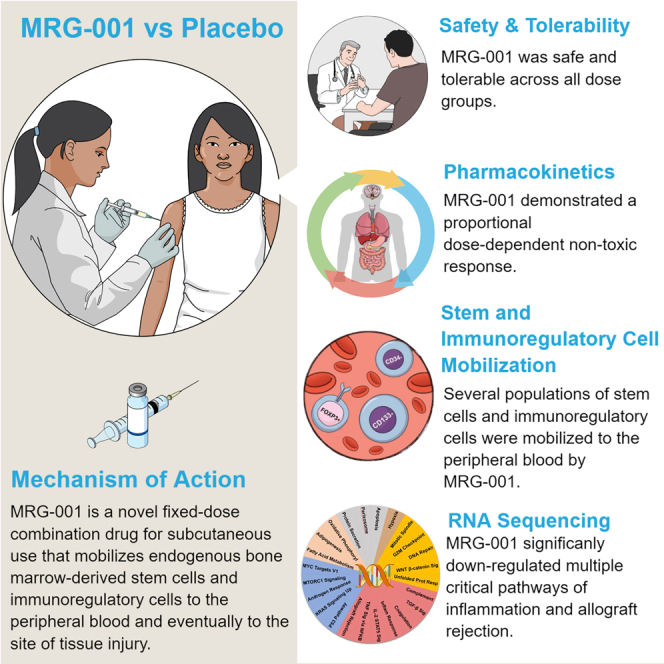

## Teaser

Ahmadi et al. show that MRG-001, a fixed-dose combination drug, is safe and mobilizes stem and immunoregulatory cells to the circulation, which could act to induce repair and regeneration and modulate inflammation. MRG-001 can potentially be used for treating a variety of human diseases caused by tissue injury and inflammation.

## Introduction

The common thread in a variety of diseases is that injury triggers inflammation, while inflammation intensifies injury and the damaged tissues heal with fibrosis. This is true for liver, kidney, heart, lung, and intestinal diseases or skin damage, such as burn wounds. An ideal therapy for injured organs is not only to regulate (hyper)inflammation, but also to promote tissue repair and regeneration. The bone marrow is a reservoir of stem cells such as hematopoietic stem cells (HSCs), endothelial progenitor cells (EPCs), mesenchymal stem cells, stage-specific embryonic antigen 3 (SSEA3^+^) Muse cells, and immunoregulatory cells including Foxp3^+^ regulatory T cells (Tregs). Recruiting these primordial cells to differentiate at the injured site may regulate inflammation and promote tissue repair.

In searching for the mechanisms of liver allograft tolerance, Dr. Sun’s group at the Johns Hopkins University School of Medicine discovered a synergy between AMD3100 (plerixafor) and low-dose FK506 (tacrolimus) (administered at less than one-tenth of the immunosuppressive dose) when injected subcutaneously together. This synergism resulted in healing via the mobilization of bone marrow stem cells and immunoregulatory cells and their recruitment into the sites injured by rejection^1^ or surgery.^2^ AMD3100 (plerixafor), an antagonist of CXCR4, was approved by the US Food and Drug Administration (FDA) in 2008 as a treatment to mobilize CD34^+^ HSCs to the peripheral blood for collection and subsequent autologous transplantation in patients with non-Hodgkin’s lymphoma or multiple myeloma.^3^ FK506 (tacrolimus), an immunosuppressant, was approved by the FDA in 1994, and used in higher doses than in MRG-001 that were clinically effective for the prophylaxis of organ rejection in patients undergoing allogeneic organ transplants. Previous non-clinical studies have demonstrated that short-term treatment with AMD3100 combined with low-dose FK506 (AF) resulted in long-term liver^1^ and kidney^4^^,^^5^ allograft survival without immunosuppression through induction of allograft chimerism^6^^,^^7^^,^^8^ that resulted in allograft acceptance. In addition, the AF combination accelerated cutaneous wound healing and decreased scar formation in healthy animals^2^^,^^9^ and in severely diabetic animals in excisional wound healing,^10^ promoted liver regeneration,^11^ prevented intra-abdominal adhesions after surgery,^12^ and ameliorated inflammatory bowel disease.^13^ These animal studies indicate that AF combination could represent a promising immunoregulatory and regenerative therapy for the treatment of a variety of human diseases related to inflammation and tissue injury. Mechanistic studies demonstrated that the synergistic effect of AF combination in immunoregulation and tissue repair is not established through calcineurin-dependent immunosuppression, but rather because of the ability of a low, but not immunosuppressive, dose of FK506 to activate the bone morphogenic protein (BMP) pathway.^14^

Based on these findings, a fixed-dose combination drug containing plerixafor and low-dose tacrolimus, named MRG-001, was developed for subcutaneous (SC) injection by MedRegen, LLC. Preclinical toxicity and pharmacokinetic and pharmacodynamic studies in rats and pigs with more than 6 weeks of treatment every other day demonstrated the drug’s preclinical safety. Here we report the results of the first-in-human trial of MRG-001 in healthy subjects.

## Results

### Disposition of subjects

A total of 21 subjects were randomized into the study and received at least one dose of their assigned study drug. A total of 18 subjects (85.7%) received all doses per protocol. Eighteen subjects (85.7%) completed the study, while 3 subjects (14.3%) discontinued early. One subject (4.8%) withdrew consent and 2 subjects (9.5%) were discontinued because of protocol violations.

### Baseline characteristics

The mean age of the subjects was 32.4 years (Table 1). Overall, females outnumbered males by 2:1. Each dose group also had more females than males enrolled. The majority of subjects were of Hispanic or Latino ethnicity (n = 13, 61.9%), with the remainder being White. The MRG-001 groups were all approximately one-half Hispanic or Latino, while the pooled placebo was less diverse with six subjects (85.7%) of Hispanic or Latino ethnicity. The overall mean height was 166.27 cm. The dose groups were well matched and within 5% of the overall mean.

**Table 1:** Demographic and other baseline characteristics

Parameter	Statistic	Cohort 1 MRG-001 0.005 mL/kg (N = 4) n (%)	Cohort 2 MRG-001 0.01 mL/kg (N = 5) n (%)	Cohort 3 MRG-001 0.02 mL/kg (N = 5) n (%)	Pooled placebo (N = 7) n (%)	Overall (N = 21) n (%)
Age (years)	n	4	5	5	7	21
	Mean	33.5	32.4	36.2	29.1	32.4
	Min	21	26	27	20	20
	Max	42	36	44	35	44

Gender						

Female	n (%)	3 (75.0)	3 (60.0)	3 (60.0)	5 (71.4)	14 (66.7)
Male	n (%)	1 (25.0)	2 (40.0)	2 (40.0)	2 (28.6)	7 (33.3)

Race						

Asian	n (%)	0	0	1 (20.0)	0	1 (4.8)
Black or African American	n (%)	0	1 (20.0)	2 (40.0)	1 (14.3)	4 (19.0)
White	n (%)	4 (100.0)	4 (80.0)	2 (40.0)	6 (85.7)	16 (76.2)

Ethnicity						

Hispanic or Latino	n (%)	2 (50.0)	3 (60.0)	2 (40.0)	6 (85.7)	13 (61.9)
Not Hispanic or Latino	n (%)	2 (50.0)	2 (40.0)	3 (60.0)	1 (14.3)	8 (38.1)
Height (cm)	n	4	5	5	7	21
	mean	169.98	168.14	164.46	164.11	166.27
	min	155.5	158.8	153.2	158.0	153.2
	max	185.6	176.0	175.5	172.6	185.6
Weight (kg)	n	4	5	5	7	21
	mean	69.23	76.24	73.60	69.84	72.14
	min	59.1	61.3	52.2	55.4	52.2
	max	87.0	92.7	96.6	76.7	96.6
BMI (kg/m^2^)	n	4	5	5	7	21
	mean	24.00	26.90	26.80	25.89	25.99
	min	20.2	22.2	21.9	22.2	20.2
	max	28.3	30.7	31.4	29.1	31.4

Abbreviations: BMI = body mass index; max = maximum; min = minimum; n = number of non-missing observations; N = total number of subjects in respective category.

The overall mean weight was 72.14 kg. The dose groups were all within 10% of the overall mean with the MRG-001 0.005 mL/kg group being the lightest (69.23 kg) and the MRG-001 0.01 mL/kg group being the heaviest (76.24 kg). The overall BMI was 25.99 kg/m^2^. The dose groups were well matched and within 5% of the overall mean.

### Safety and treatment-emergent adverse events

No deaths, no serious adverse events, or significant adverse events leading to discontinuation of the study or study drug were reported (Table 2). There were no clinically significant vital signs or ECG abnormalities. Overall, 11 subjects (52.4%) reported 38 treatment-emergent adverse events (TEAE) after study drug treatment; 2 subjects (50.0%) reported 6 TEAE after treatment with MRG-001 0.005 mL/kg, 3 subjects (60.0%) reported 5 TEAEs after treatment with MRG-001 0.01 mL/kg, 5 subjects (100.0%) reported 26 TEAE after treatment with MRG-001 0.02 mL/kg, and 1 subject (14.3%) reported 1 TEAE after treatment with placebo.

**Table 2:** Summary of TEAEs by system organ class and preferred term (safety set)

System organ class Preferred term	Cohort 1 MRG-001 0.005 mL/kg (N = 4) n (%)^c^		Cohort 2 MRG-001 0.01 mL/kg (N = 5) n (%)^c^		Cohort 3 MRG-001 0.02 mL/kg (N = 5) n (%)^c^		Pooled placebo (N = 7) n (%)^c^		Overall (N = 21) n (%)^c^	
	No. of subjects (%)^a^	No. of events^b^	No. of subjects (%)^a^	No. of events^b^	No. of subjects (%)^a^	No. of events^b^	No. of subjects (%)^a^	No. of events^b^	No. of subjects (%)^a^	No. of events^b^
Subjects with any TEAE	2 (50.0)	6	3 (60.0)	5	5 (100.0)	26	1 (14.3)	1	11 (52.4)	38
Gastrointestinal disorders	2 (50.0)	2	2 (40.0)	2	3 (60.0)	8	0	0	7 (33.3)	12
Abdominal pain	0	0	0	0	1 (20.0)	3	0	0	1 (4.8)	3
Diarrhea	1 (25.0)	1	1 (20.0)	1	1 (20.0)	1	0	0	3 (14.3)	3
Dry mouth	1 (25.0)	1	0	0	0	0	0	0	1 (4.8)	1
Nausea	0	0	1 (20.0)	1	2 (40.0)	4	0	0	3 (14.3)	5
General disorders and administration site conditions	1 (25.0)	1	2 (40.0)	2	5 (100.0)	12	1 (14.3)	1	9 (42.9)	16
Influenza like illness	0	0	0	0	1 (20.0)	1	0	0	1 (4.8)	1
Injection site erythema	0	0	0	0	1 (20.0)	1	0	0	1 (4.8)	1
Injection site hemorrhage	0	0	0	0	1 (20.0)	1	0	0	1 (4.8)	1
Injection site pain	1 (25.0)	1	2 (40.0)	2	4 (80.0)	8	1 (14.3)	1	8 (38.1)	12
Injection site swelling	0	0	0	0	1 (20.0)	1	0	0	1 (4.8)	1
Infections and infestations	0	0	0	0	1 (20.0)	1	0	0	1 (4.8)	1
Vulvovaginal candidiasis	0	0	0	0	1 (20.0)	1	0	0	1 (4.8)	1
Musculoskeletal and connective tissue disorders	0	0	0	0	1 (20.0)	1	0	0	1 (4.8)	1
Musculoskeletal chest pain	0	0	0	0	1 (20.0)	1	0	0	1 (4.8)	1
Nervous system disorders	2 (50.0)	3	1 (20.0)	1	2 (40.0)	3	0	0	5 (23.8)	7
Dizziness	0	0	0	0	1 (20.0)	1	0	0	1 (4.8)	1
Headache	2 (50.0)	3	0	0	1 (20.0)	1	0	0	3 (14.3)	4
Parasthesia	0	0	1 (20.0)	1	0	0	0	0	1 (4.8)	1
Somnolence	0	0	0	0	1 (20.0)	1	0	0	1 (4.8)	1
Psychiatric disorders	0	0	0	0	1 (20.0)	1	0	0	1 (4.8)	1
Insomnia	0	0	0	0	1 (20.0)	1	0	0	1 (4.8)	1

a Subject will be counted only once in each category for more than 1 event.

b Subject can be represented more than once.

c N = total number of subjects in respective category.

All TEAE were considered mild (n = 34) or moderate (n = 4) in severity; there were no serious TEAE. The majority of TEAE were definitely (9 subjects [42.9%], 15 TEAE), probably (2 subjects [9.5%], 2 TEAE), or possibly (7 subjects 33.3[%], 16 TEAE) related.

In the MRG-001 0.005 mL/kg group (n = 4), two subjects (50.0%) reported six TEAE including diarrhea, dry mouth, injection site pain, and headache. In the MRG-001 0.01 mL/kg group (n = 5), three subjects (60.0%) reported five TEAE including diarrhea, nausea, injection site pain, and paresthesia. In the MRG-001 0.02 mL/kg group (n = 5), 5 subjects (100.0%) reported 26 TEAE, including abdominal pain, diarrhea, nausea, influenza-like illness, injection site erythema, injection site hemorrhage, injection site pain, injection site swelling, vulvovaginal candidiasis, musculoskeletal chest pain, dizziness, headache, somnolence, and insomnia.

In the pooled placebo group (n = 7), one subject (14.3%) reported one mild, definitely related TEAE of injection site pain. Injection site reaction TEAE were noted in all three MRG-001 groups, with the MRG-001 0.02 mL/kg group having the greatest numbers. In the 0.005 mL/kg group, one subject had a mild TEAE of injection site pain that had a duration of 5 h. Two subjects in the 0.01 mL/kg group each had one mild TEAE of injection site pain with a duration of 10 min and 2 h 9 min. All five subjects in the 0.02 mL/kg group experienced injection site reaction TEAE. The most common injection site reaction TEAE was injection site pain (verbatim term: burning at the injection site) of mild (10 TEAE) or moderate (2 TEAE) intensity, with durations ranging from 15 min to 3 h. The only other injection site-related TEAE were reported in a single subject receiving MRG-001 0.02 mL/kg and included injection site erythema (mild), injection site swelling (mild), and injection site hemorrhage (moderate), all lasting approximately 1 day.

There were no clinically significant abnormalities for chemistry, cardiac troponins, coagulation, or urinalysis parameters. The only significant abnormality, which had no clinical impact, was the increase of mean white blood cells (WBC) and its lineages in the peripheral circulation in subjects treated with MRG-001, as was expected. On average the WBC counts in test subjects increased 2- to 4-fold from baseline, while the placebo subjects did not show any increase.

### Pharmacokinetics: Plerixafor and tacrolimus

#### Plerixafor

Subjects received MRG-001 on day 1, 3 and 5. Plerixafor PK was evaluated after a subject received a single dose on day 1 and after the last dose administration on day 5. The mean plasma concentration-time profiles of plerixafor increased with dose on day 1 and day 5. The elimination appeared to be monophasic for all three doses after dosing on day 1 and day 5. One treated subject had an aberrant value (BLQ) at 3 h after the dose on day 1 for plerixafor. As this sample was at the potential time to maximum plasma concentration (T_max_), PK parameters were excluded from the summaries.

**Figure 1:**
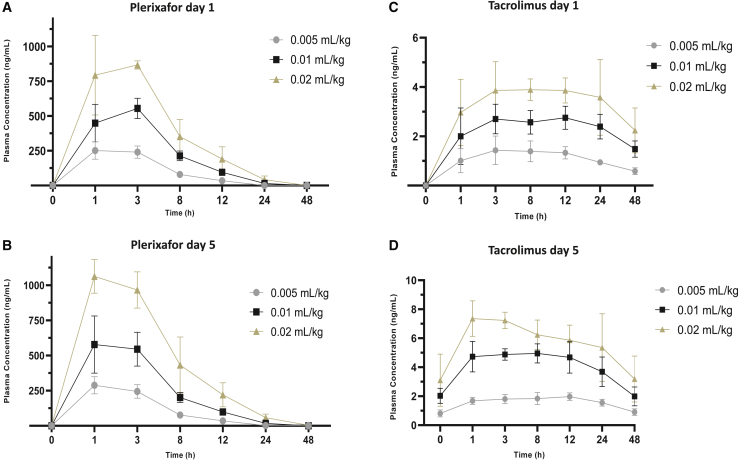
MRG-001 pharmacokinetics (A) Time profiles for plerixafor following SC administration of a single dose of 0.005, 0.01, and 0.02 mL/kg MRG-001 on day 1. (B) Time profiles for plerixafor following SC administration of 0.005, 0.01, and 0.02 mL/kg MRG-001 on day 5. (C) Time profiles for tacrolimus following SC administration of a single dose of 0.005, 0.01, and 0.02 mL/kg MRG-001 on day 1. (D) Time profiles for tacrolimus following SC administration of 0.005, 0.01, and 0.02 mL/kg MRG-001 on day 5. n = 4 per group. Bars represent standard deviation of the mean.

Plasma PK parameters for plerixafor following SC administration of MRG-001 on day 1 and day 5 are summarized in Table S1 and Figures 1A and 1B. The inter-subject variability as represented by the GeoCV% for peak plasma concentrations (C_max_) and area under the curve (AUC) was low (ranging from 7.5% to 23.1%) for all dose levels on days 1 and 5.

The geometric mean C_max_ of plerixafor increased with increasing dose of MRG-001 on day 1 and day 5. The median time to C_max_ was approximately 1–3 h after either on day 1 or day 5. The geometric mean terminal half-life (t_1/2_) ranged from 3 to 6 h. Plerixafor was fully eliminated at 48 h across all dosages.

#### Tacrolimus

Subjects received MRG-001 on day 1, 3 and 5. Tacrolimus PK was evaluated after a subject received a single dose on day 1 and after the last dose administration on day 5. The mean whole blood tacrolimus concentration-time profiles on day 1 and day 5 are presented in Table S2 and Figures 1C and 1D. The mean whole blood concentration-time profiles of tacrolimus increased with dose on day 1 and day 5. The elimination phase was not present in many of the subjects during the 48-h sample period for day 1 and seemed to be monophasic following dosing on day 5.

Whole blood PK parameters for tacrolimus following SC administration of MRG-001 on day 1 and day 5 are summarized in Table S2. The intersubject variability (GeoCV%) for C_max_ and AUC was low to moderate (ranging from 10.8% to 34.7%) for all dose levels on days 1 and 5.

The GM C_max_ of tacrolimus increased with increasing dose of MRG-001 on both day 1 and day 5. The median T_max_ was approximately 2–12 h following MRG-001 dosing either on day 1 or day 5. The GM t_1/2_ ranged from 24 to 38 h.

The trough concentration (C_trough_) of tacrolimus increased with an increasing dose of MRG-001 on both day 1 and day 5. The median C_trough_ was 0.58, 1.48, and 2.24 ng/mL, respectively, with increasing dosages on day 1. On day 5, the C_trough_ were 0.90, 1.99, and 3.19 ng/mL.

In summary, after SC administration of a single dose on day 1 and after multiple doses to day 5 of MRG-001 0.005, 0.01, and 0.02 mL/kg (24 mg plerixafor plus 0.5 mg tacrolimus per mL), the C_max_ and AUC for both plerixafor and tacrolimus increased with increasing dose in what seems to be a dose-proportional manner. The t_1/2_ for plerixafor was short for all doses (3–6 h) and longer for tacrolimus (24–38 h).

### Pharmacodynamics: Mobilization of immune cells and bone marrow stem cells

#### White Blood Cell mobilization

**Figure 2:**
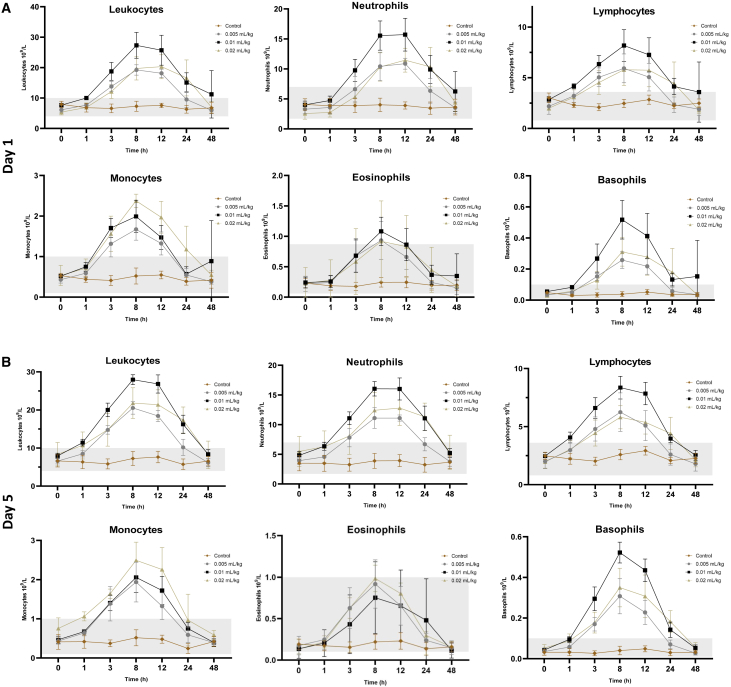
WBC mobilization with MRG-001 in healthy subjects Subjects received SC injections every other day of saline (placebo control, n = 6), low-dose (0.005 mL/kg), mid-dose (0.01 mL/kg), or high-dose (0.02 mL/kg) MRG-001 (n = 4/group). Venous blood was collected to determine the WBC differential count at several time intervals. (A) WBCs on day 1 after a single dose SC injection of saline or MRG-001. (B) WBCs on day 5 after the third dose SC injection of saline or MRG-001. Each value represents the mean ± SD. The gray area represents the normal reference range.

The WBC count was increased by approximately 2- to 4-fold from baseline levels after injection for all MRG-001-treated subjects, reached the peak levels between 8 and 12 h, and returned to baseline levels or near baseline levels at 48 h (Figure 2A). Interestingly, mid-dose MRG-001 (0.01 mL/kg) mobilized more leukocytes, neutrophils, lymphocytes, and basophils at 3, 8, and 12 h (peak levels) than high-dose (0.02 mL/kg) or low-dose (0.005 mL/kg) MRG-001. MRG-001 consistently mobilized WBC in a similar pattern on day 5 after the third dose injection (Figure 2B).

#### Mobilization of immunoregulatory T cells to the peripheral blood

**Figure 3:**
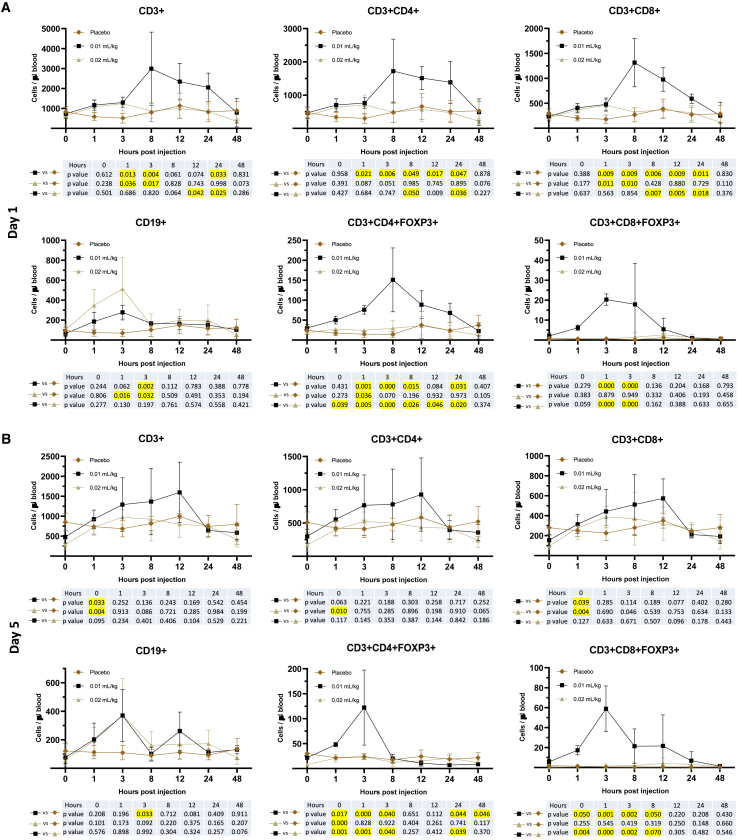
Lymphocyte mobilization with MRG-001 in healthy subjects Subjects received SC injections every other day of saline (placebo control), mid-dose (0.01 mL/kg), or high-dose (0.02 mL/kg) MRG-001 (n = 4/group). Venous blood was collected and PBMCs were isolated at different time intervals on day 1 after a single dose SC injection of saline or MRG-001 and on day 5 after the third dose. Lymphocyte populations (CD3^+^, CD3^+^CD4^+^, CD3^+^CD8^+^, CD19^+^, CD3^+^CD4^+^Foxp3^+^, and CD3^+^CD8^+^Foxp3^+^) in PBMC were analyzed by flow cytometry. At each blood draw timepoint, absolute number of circulating lymphocyte subsets, such as CD3^+^ T cells, was calculated by multiplication of CD3^+^ cell fraction of PBMC by the total circulating PBMC count. (A) Lymphocyte populations in peripheral blood on day 1 after a single dose SC injection of saline or MRG-001. (B) Lymphocyte populations in peripheral blood on day 5 after the third dose. Each value represents the mean ± SD. Yellow marked cells represent significant difference with p < 0.05.

In the mid-dose group (0.01 mL/kg), the numbers of CD3^+^, CD4^+^, and CD8^+^ T cells in the peripheral blood increased by approximately 2- to 3-fold from baseline with peak levels at 8 h and decreased back to placebo levels at 48 h (Figure 3A), while CD19^+^ B cells increased 3-fold at 3 h and decreased back to baseline levels at 8 h after first dose (day 1). Interestingly, CD3^+^CD4^+^Foxp3^+^ Tregs increased 3-, 5-, and 10-fold at 1, 3, and 8 h and remained at higher levels (3-fold) at 24 h after the first mid dose, while CD3^+^CD8^+^Foxp3^+^ Tregs increased 10- and 30-fold at 1 and 3 h and decreased back to baseline levels at 24 h. In the high-dose group (0.02 mL/kg), the numbers of CD3^+^, CD4^+^, and CD8^+^ T cells in peripheral blood increased by approximately 2- to 3-fold and CD19^+^ B cells increased by 4- to 6-fold at 1 and 3 h, and decreased back to baseline levels at 8 h after the first high dose SC. However, neither CD4^+^Foxp3^+^ nor CD8^+^Foxp3^+^ Tregs were increased in the high-dose group.

At day 5 (0 h, before the third dose), the numbers of CD3^+^, CD4^+^, and CD8^+^ T cells and CD4^+^Foxp3^+^ Tregs were significantly lower in MRG-001 groups compared with the placebo group (Figure 3B). The third dose of MRG-001 increased CD3^+^, CD4^+^, and CD8^+^ T cells to levels that were similar to the placebo group at 1 h, but the levels of these cells in blood were not significantly higher in MRG-001 treatment groups than the placebo group at different time points (p > 0.05). There was no significant difference in CD19^+^ B cells at day 5 (0 h), and the mid-dose but not high-dose MRG-001 significantly increased CD19^+^ B cells (3-fold) at 3 h after the third dose. Consistently, CD3^+^CD4^+^Foxp3^+^ Tregs increased 2- and 5-fold at 1 and 3 h and decreased to the placebo levels at 8 h, while CD3^+^CD8^+^Foxp3^+^ Tregs increased approximately 12-, 112-, and 80-fold at 1, 3, and 8 h and decreased to the placebo levels at 24 h after the third of mid-dose MRG-001. High-dose MRG-001 did not increase Foxp3^+^ Tregs.

To better reflect the immune balance during mobilization, the ratio between Tregs and T cells was calculated (Figure S1). In the control group, the ratios of Tregs in CD4^+^ T cell population were 5.3% ± 1.0% (day 1) and 5.8% ± 0.5% (day 5). The highest ratios of Tregs in CD4^+^ T cells among all groups was from the mid-dose group (0.01 mL/kg) at 10.2% ± 2.5% (day 1) and 16% ± 0.8% (day 5). However, no significant increase in the ratios of Tregs in CD4^+^ T cells was observed in the high dose group (7.5% ± 6.5% on day 1 and 5.0% ± 0.8% on day 5). Similarly, in comparison to the control group (0.3% ± 0.5% on day 1 and 0.5% ± 1.0% on day 5), the ratios of Tregs in CD8^+^ T cells at the peak of mobilization were dramatically increased to 4.5% ± 1.3% (day 1) and 14.3% ± 2.2% (day 5) in the mid-dose dose group (0.01 mL/kg), but there was no increase in the high-dose group (undetectable on day 1 and 0.3% ± 0.5% on day 5). These results indicate that the mid-dose of MRG-001 significantly increased the ratios of Tregs in both CD4^+^ and CD8^+^ T cell populations.

The mid-dose of MRG-001 consistently increased CD4^+^Foxp3^+^ and CD8^+^Foxp3^+^ Tregs on day 1 and day 5, suggesting its potential for immunoregulation.

#### Mobilization of bone marrow stem cells and progenitor cells to the peripheral blood

**Figure 4:**
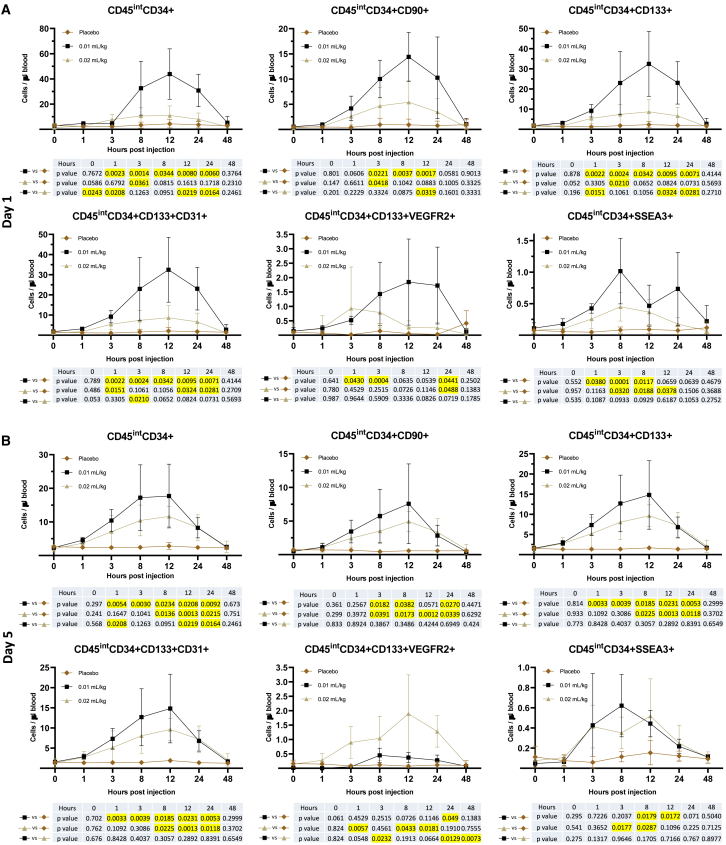
Stem cell mobilization with MRG-001 Subjects received SC injections every other day of saline (placebo control), mid-dose (0.01 mL/kg), or high-dose (0.02 mL/kg) MRG-001 (n = 4/group). Venous blood was collected and PBMCs were isolated at different time intervals on day 1 after a single dose SC injection of saline or MRG-001 and on day 5 after the third dose. Stem cell populations (CD45^Int^CD34^+^, CD45^Int^CD34^+^CD90^+^, CD45^Int^CD34^+^CD133^+^, CD45^Int^CD34^+^CD133^+^CD31^+^, CD45^Int^CD34^+^CD133^+^VEGFR2^+^, CD45^Int^CD34^+^SSEA3^+^) in PBMC were analyzed by flow cytometry. At each blood draw timepoint, absolute number of circulating stem cells, such as CD45^Int^CD34^+^ HSCs, was calculated by multiplication of CD45^Int^CD34^+^ cell fraction of PBMC by the total circulating PBMC count. (A) Stem cells in peripheral blood on day 1 after a single dose SC injection of saline or MRG-001. (B) Stem cells in peripheral blood on day 5 after the third dose. Each value represents the mean ± SD. Yellow marked cells represent significant difference with p < 0.05.

In the mid-dose group (0.01 mL/kg), after administration of MRG-001, CD45^Int^CD34^+^, and CD45^Int^CD34^+^CD133^+^ HSCs and CD45^Int^CD34^+^CD133^+^CD31^+^ EPCs in peripheral blood increased at 1 h, reached peak levels at 12 h, remained at higher levels at 24 h, and decreased back to placebo levels at 48 h (Figure 4A). At 12 h, these stem/progenitor cells increased approximately 15- to 17-fold from baseline levels (pre-dose) and the peak CD45^Int^CD34^+^ HSCs were 43.86/μL blood. CD45^Int^CD34^+^CD90^+^ multipotent HSCs with long-term multilineage engraftment potential^15^ increased approximately 7-, 18-, and 26-fold from baseline levels at 3, 8, and 12 h. Compared with the placebo group, CD45^Int^CD34^+^CD133^+^VEGFR2^+^ early EPCs increased approximately 3-, 28-, and 60-fold at 1, 3, and 24 h, while SSEA3-expressing HSCs (CD45^Int^CD34^+^SSEA3^+^) also increased 3-, 8-, and 12-fold at 1, 3, and 8 h. In the high-dose group (0.02 mL/kg), stem cell populations were increased in a similar pattern but to a moderate degree, and the peak CD45^Int^CD34^+^ HSCs were 11/μL blood.

After administration of the third dose at day 5, MRG-001 consistently mobilized stem cells and progenitor cells in a similar pattern (Figure 4B), but the numbers of mobilized stem cells and progenitor cells were lower compared with day 1 and the peak CD45^Int^CD34^+^ HSCs were 17.7/μL in the mid-dose group. More stem cells and progenitor cells were mobilized in the mid-dose group than in the high-dose group except for CD45^Int^CD34^+^CD133^+^VEGFR2^+^ early EPCs.

### MRG-001 down-regulates pathways associated with inflammation and allograft rejection

**Figure 5:**
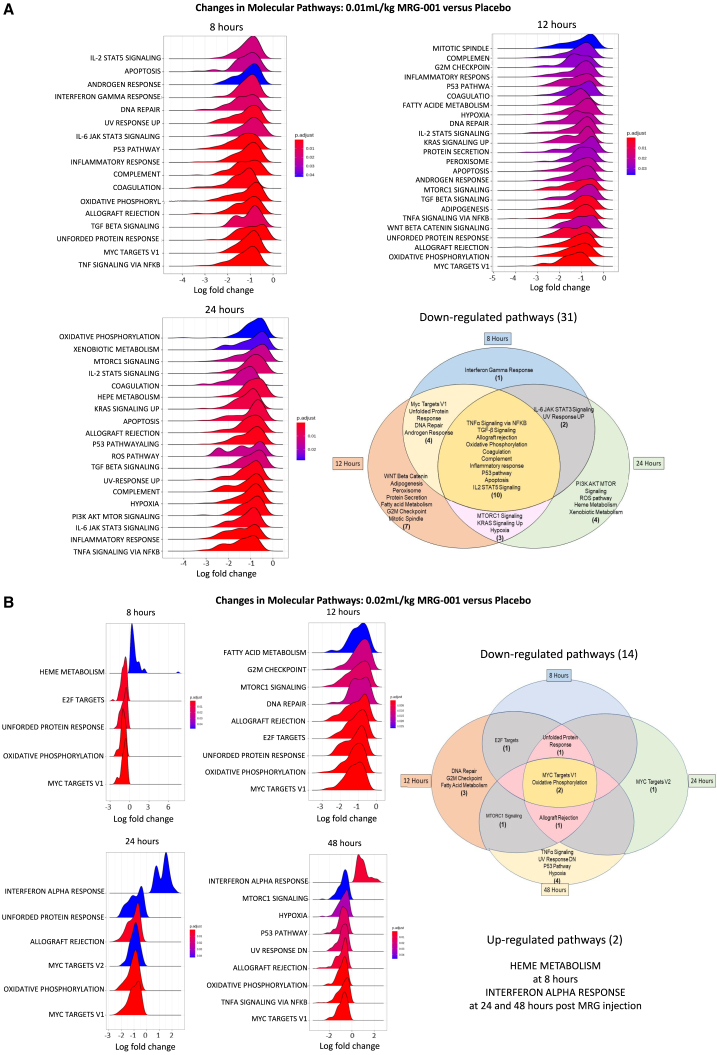
Regulated molecular pathways by MRG-001 RNA sequencing transcriptome studies of PBMCs were performed at different time points following a single dose SC injection of saline (placebo) or MRG-001. (A) GSEA for interpreting gene expression profiles revealed 17, 24 and 19 down-regulated pathways at 8, 12, and 24 h after administration of 0.01 mL/kg MRG-001. A total of 31 down-regulated pathways were recognized and summarized in a Venn diagram. (B) GSEA showed 4, 9, 5, and 8 down-regulated pathways at 8, 12, 24, and 48 h, and 1 up-regulated pathway at 8, 24, and 48 h after administration of 0.02 mL/kg MRG-001. A total of 14 down-regulated and 2 up-regulated pathways were recognized, and down-regulated pathways were summarized in a Venn diagram.

RNA sequencing transcriptome studies of peripheral blood mononuclear cells (PBMC) showed no change in gene expression at different time points in the placebo treatment group over the measured 48-h period, which spans one injection cycle. Significant differences in gene expression between placebo and 0.01 mL/kg MRG-001 or 0.02 mL/kg MRG-001 injection were observed (Table S3). Significant changes in gene expression occurred in the group treated with the mid-dose of MRG-001 (0.01 mL/kg) including 850 down-regulated genes and 1,049 up-regulated genes at 8 h, 1,474 down-regulated genes and 825 up-regulated genes at 12 h, and 270 down-regulated genes and 18 up-regulated genes at 24 h after injection. In contrast with the middle dose group, a high dose of MRG-001 treatment (0.02 mL/kg) resulted in fewer changes in gene expression, including 66 down-regulated genes and 25 up-regulated genes at 1 h, 1 up-regulated gene at 3 h, 7 down-regulated genes and 32 up-regulated genes at 8 h, 484 down-regulated genes and 202 up-regulated genes at 12 h, 202 down-regulated genes and 55 up-regulated genes at 24 h, and 70 down-regulated genes and 8 up-regulated genes at 48 h. To discover the biological pathways affected by different dosages of MRG-001, gene set enrichment analysis (GSEA) was used. We specifically studied the time point where the most genetic changes occurred, which was at 8, 12, 24, and 48 h. Compared with the placebo group, 17, 24, and 19 down-regulated pathways were identified at 8, 12, and 24 h in the mid-dose group (Figure 5A), while 4, 9, 5, and 8 down-regulated pathways at 8, 12, 24, and 48 h and 1 up-regulated pathway at 8, 24, and 48 h were identified in the high-dose group (Figure 5B). Totally, the GSEA of RNA sequencing data identified 31 down-regulated pathways in the mid-dose group and 14 down-regulated pathways and 1 up-regulated pathway in the high-dose group. Ten down-regulated pathways including tumor necrosis factor (TNF)-alpha signaling via nuclear factor (NF)-κB, transforming growth factor (TGF)-beta signaling, allograft rejection, oxidative phosphorylation, coagulation, complement, inflammatory response, P53 pathway, apoptosis, and IL-2 STAT5 signaling were consistently present at 8, 12, and 24 h in the mid-dose group, while four pathways, including MYC target V1, oxidative phosphorylation, allograft rejection, and unfolded protein response, were consistently down-regulated at 8, 12, 24, and/or 48 h at the high dose.

Considering the diversity of testing subjects, we have also compared gene expression at different time points to pre-dose gene expression. Compared with the pre-dose, 654 down-regulated genes and 1,038 up-regulated genes at 8 h, 630 down-regulated genes and 225 up-regulated genes at 12 h, 199 down-regulated genes and 9 up-regulated genes at 24 h, and 1 up-regulated gene at 48 h were identified in the mid-dose group (Table S4). Fifteen down-regulated genes and 29 up-regulated genes at 8 h, 132 down-regulated genes and 72 up-regulated genes at 12 h, and 78 down-regulated genes and 43 up-regulated genes at 24 h were identified in the high-dose group (Table S4). Totally, the GSEA of RNA sequencing data identified 1 up-regulated pathway (Interferon alpha response) and 29 down-regulated pathways in the middle dose group (Figure S2A), and 5 down-regulated pathways in the high-dose group (Figure S2B). When comparing the common down-regulated pathways between the intermediate (0.01 mL/kg) and the high dose (0.02 mL/kg), only six pathways are commonly down-regulated all at 24 h (Table S5).

Venn diagram analysis of common down-regulated pathways between MRG-001 versus placebo and MRG-001 versus pre-dose revealed 27 common down-regulated pathways in the mid-dose group and 4 down-regulated pathways in the high-dose group (Figure S3). Most down-regulated pathways (27/31) in the mid-dose group compared with the placebo group were also observed in the mid-dose group comparison with the pre-dose gene expression (27/29).

## Discussion

In this first-in-human study, MRG-001 was safe and well tolerated at doses of up to 0.02 mL/kg administered every other day subcutaneously SC in the three multiple ascending dose cohorts in healthy subjects. There were no deaths, serious adverse events, or severe TEAE. Most TEAE were mild, and there was a dose-dependent increase in TEAE frequency; 68% of TEAEs (26/38) including four moderate TEAEs were observed in the high-dose cohort. These events were temporary and all were resolved within 24–48 h. No clinically significant laboratory changes were observed besides the expected mobilization of different lineages of WBCs.

After multiple dose administration, plasma plerixafor and tacrolimus C_max_ and AUC generally increased in an apparent dose-proportional manner across the dose range studied. MRG-001 PK parameters were comparable for single and repeated SC injection, suggesting no time-dependent changes in PK in this study. The PK of both plerixafor and tacrolimus was dose proportional and never reached toxic or immunosuppressive thresholds. The 0.01 mL/kg dose group demonstrated the desired tacrolimus range based on previous preclinical studies^1^^,^^2^^,^^4^^,^^5^^,^^10^^,^^11^^,^^12^^,^^13^ and blood trough levels were less than 2 ng/mL on day 1 or 3 ng/mL on day 5 after injection, suggesting no immunosuppression. Tacrolimus levels of less than 2 ng/mL are generally below the LLQ in clinical laboratories and at or greater than 5 ng/mL are considered immunosuppressive, as also stated by the tacrolimus package insert.^16^^,^^17^

The action of MRG-001 in animal models of human diseases is to mobilize bone marrow stem cells and immunoregulatory cells to the peripheral blood and recruit mobilized stem cells and immunoregulatory cells into the injured sites.^1^^,^^2^^,^^4^^,^^5^^,^^10^^,^^11^^,^^12^^,^^13^ In this study, MRG-001 is effective in mobilizing a variety of stem/progenitor cells and immune cells including CD4^+^Foxp3^+^ and CD8^+^Foxp3^+^ Tregs, which is consistent with the findings in animal models. Interestingly, stem cell and immune cell mobilization was not dose proportional in MRG-001-treated subjects. Mid-dose MRG-001 dramatically mobilized bone marrow stem cells such as HSCs and CD4^+^ and CD8^+^ Tregs, and peak CD45^Int^CD34^+^ HSCs were 43.86/μL blood (Figure 4A) that is greater than reported results from a plerixafor trial.^18^ The normal dose of plerixafor (0.24 mg/kg) mobilizes 27.8 CD34^+^HSCs/μL at peak levels, while high-dose plerixafor (0.48 mg/kg) mobilizes greater numbers of CD34^+^ HSCs (peak CD34^+^, 32.2 HSCs/μL). However, high-dose MRG-001 (plerixafor 0.48 mg/kg) mobilized stem cells moderately (peak CD34^+^HSCs, 10.99/μL) without increasing Tregs. Animal studies demonstrated that low-dose, but not high-dose, FK506 reacted synergistically with AMD3100 (plerixafor) in mobilizing stem cells and Tregs^1^^,^^2^ through activation of BMP signaling.^14^ Increasing FK506 levels may impact the synergism with AMD3100 and, therefore, decrease the efficacy of high-dose MRG-001 in mobilizing stem cells and Tregs.

All cell populations returned to baseline levels before the next injection, consistent with there being no accumulative effect. As expected, control subjects who received placebo showed no evidence of diurnal variation of leukocyte subset levels across the measured timepoints. In animal models of human diseases, mobilized stem cells and immunoregulatory cells are recruited into the injured organ or tissues and promoted tissue repair and regeneration.^1^^,^^2^^,^^4^^,^^5^^,^^10^^,^^11^^,^^12^^,^^13^ However, in healthy subjects, these mobilized cells stay in circulation for up to 24 h and likely return to the bone marrow reservoir.

Foxp3^+^ Tregs have a critical role in the maintenance of immune homeostasis, prevention of autoimmunity, and induction of allograft tolerance.^19^ Expanding and/or infusion of Tregs can effectively prevent allograft rejection and cure autoimmune diseases without significant side effects.^20^ Indeed, their functions extend beyond immune surveillance to tissue homeostasis, including regulation of local and systemic metabolism, promotion of tissue repair and regeneration, and control of the proliferation, differentiation, and fate of non-lymphoid cell progenitors.^21^ MRG-001 given at the mid-dose dramatically increased circulating CD4^+^Foxp3^+^ and CD8+Foxp3^+^ Tregs suggesting its immunomodulatory properties and that MRG-001 could be used as an immunoregulatory therapy in a variety of human diseases including autoimmune diseases and transplant rejection.

Circulating hematopoietic stem and progenitor cells (HSPCs) including CD34^+^ and CD34^+^CD133^+^ are rare, but changes in circulating HSPCs were reported to relate to the outcomes of several diseases in the cardiovascular system, lung, kidney and liver. Low CD34^+^ and CD34^+^CD133^+^ cell levels significantly and independently predicted the development or worsening of microangiopathy in diabetic patients^22^ and a reduced CD34^+^ and CD34^+^CD133^+^ cell count independently predicted future events in patients with type 2 diabetes.^23^ Several studies have reported that patients with low levels of circulating HSPCs were at a significantly higher risk for future cardiovascular disease than were patients with higher cell levels,^24^^,^^25^^,^^26^^,^^27^ and a decrease in circulating HSPC counts during exercise is associated with worse prognosis and is a stronger factor in outcomes than the presence of stress-induced myocardial ischemia in patients with coronary artery disease.^28^ Similarly, circulating progenitor cells are decreased in patients with severe lung disease^29^ and lower CD34^+^ HSPCs were associated with a more than 3-fold higher risk of adverse outcome in patients with coronavirus disease 2019 (COVID-19). Reduction of HSPCs was a significant mediator of the admission of hyperglycemia on COVID-19 outcome, being responsible for 28% of its prognostic effect.^30^ Conversely, a higher number of CD34^+^ and CD34^+^ CD133^+^ HSPCs was inversely associated with all-cause and cardiovascular mortality.^31^ Infusion of CD34^+^ cells in patients with chronic kidney disease improved 1-year outcome^32^ and CD34^+^ and CD133^+^ stem cells infusion has been used as a supportive treatment for end-stage liver disease with satisfactory tolerability.^33^ However, mobilization of HSPCs with plerixafor did not promote the healing of ischemic wounds and might exert adverse effects on wound healing in diabetic patients,^34^ and a similar result was reported in a mouse model of surgical wounds,^2^ suggesting the necessity of activating other pathways.^10^^,^^14^ Activation of BMP signaling by FKBP12 ligands (low-dose tacrolimus) in combination with plerixafor exhibited a synergy in mobilizing and recruitment of stem cells into the injured sites and promoted diabetic wound healing.^14^ MRG-001 consistently increased circulating stem cells following multiple-ascending dose treatment (Figure 4) indicating the potent stem cell mobilizing activity of MRG-001 and its potential as a regenerative therapy in patients with organ/tissue injury.

Notably, mobilizing stem cells and immune Tregs are associated with changes in gene expression of PBMCs. RNA sequencing of PBMCs and the GSEA revealed 27 common down-regulated pathways in the mid-dose cohort and 4 common down-regulated pathways in the high-dose cohort, compared with the placebo group or pre-dose expression profiles (Figure S3). Release from the bone marrow may cause stem cell and immune activation; however, the multitude of genes and pathways influenced by MRG-001 was unexpected. Most of these 27 pathways in PBMC are related to proinflammatory and inflammatory response, complement and coagulation activation, T and B cell activation and function, metabolic reprogramming in T cells, oxidative stress, cell death, and secreting inflammatory cytokines. Down-regulating 27 pathways suggest a number of potential activities of MRG-001 including anti-inflammation/allograft rejection (*TNFalpha signaling* via *NFκB*, *inflammatory response*, *allograft rejection*, *IL-2 STAT5 signaling*, *IL-6 JAK/STAT3 signaling*, *oxidative phosphorylation*, *etc*.), anti-ischemia/reperfusion injury (*hypoxia*, *ROS pathway*, *P53 pathway*, *mTORC1 signaling pathway*, *apoptosis*, *etc*.), anti-thrombosis (*coagulation and complement pathways*), anti-fibrosis (*WNT beta-catenin signaling*, *TGF-beta signaling*) and anti-aging (*mTORC1 signaling*, *IL-6 JAK/STAT3 signaling*, *inflammatory response*, *ROS pathway*, *unfolded protein response*, *etc*.). Indeed, animal studies have demonstrated some of these activities in a variety of animal models of human diseases,^1^^,^^2^^,^^4^^,^^5^^,^^10^^,^^11^^,^^12^^,^^13^ especially anti-allograft rejection.^1^^,^^4^^,^^5^ It is worth mentioning that several pathways (i.e., *MYC target V1*, *Kras signaling up*, *androgen response*, *peroxisome*, *unfolded protein response*) down-regulated by MRG-001 are also related to cancer and decreasing expression of these pathways such as MYC have been considered as novel therapeutic strategies for cancer treatment.^35^

Higher numbers of mobilized stem cells and immunoregulatory T cells in peripheral blood are correlated with more down-regulated pathways in PBMCs. However, the kinetics of gene expression changes did not correspond to kinetic changes of stem cells and/or immunoregulatory T cells. For example, circulating immune cells and stem cells fell back to placebo levels at 48 h in the high-dose cohort, but six down-regulated pathways remained. The mid-dose, not high-dose, mobilized more stem cells and immunoregulatory T cells and down-regulated more pathways. The peak levels of tacrolimus (2–3 ng/mL) in the middle dose cohort (Figure 1C) are within the desired range based on preclinical studies. Down-regulating 27 pathways by the middle dose MRG-001 may be caused by the synergy of plerixafor and low-dose tacrolimus. The mechanisms causing changes in particular gene expression by MRG-001 clearly warrant further investigation.

More than a decade of animal studies led to the development of MRG-001. Because of its immunoregulatory and regenerative therapeutic properties discovered from animal studies and its safety in healthy subjects, MRG-001 is being tested in a phase II trial for efficacy and safety in severely, critically ill, COVID-19 patients who suffer from acute respiratory distress syndrome (NCT04646603). A second IIa study will soon be initiated to study the safety and efficacy of MRG-001 in wound healing in pre-abdominoplasty surgical excisions (NCT05844527).

In summary, multiple doses of MRG-001 up to 0.02 mL/kg (every other day, SC injection) were safe and well tolerated in healthy participants in this study. The 0.01 mL/kg MRG-001 dose may be a promising and safe dosage for mobilizing bone marrow stem cells, immune Tregs, and down-regulating pathways related to inflammation and other diseases. Further studies in phase II clinical trials are warranted to investigate the efficacy and safety of MRG-001 in patients with inflammatory disorders, wounds, and organ transplantation.

### Limitations of the study

This study has a few limitations. First, the limitations of this trial are related to the design features of a phase I trial, namely the relatively small number of subjects in each cohort that may limit generalizability. The study did not include elderly subjects, the majority of the subjects were younger than 40 years. Second, there was significant loss of biological material of the lowest dose group (0.005 mL/kg MRG-001) because of shipping delays caused by a winter storm in Texas. This made it impossible to analyze flow cytometric and some of the RNA sequencing outcomes of this group, limiting our ability to draw a definitive conclusion on the pharmacodynamics of the lowest dose group.

## STAR★Methods

### Key resources table

**Table:** 

REAGENT or RESOURCE	SOURCE	IDENTIFIER
**Antibodies**		

Mouse Anti-Human CD4; APC -H7	BD	Cat # 560158
Mouse Anti-Human CD8; BUV805	BD	Cat # 749366
Mouse Anti-Human CD19; BUV737	BD	Cat # 741829
Mouse Anti-Human CD56; BV785	Biolegend	Cat # 362550
Mouse Anti-Human CD16; PE	BD	Cat # 555407
Mouse Anti-Human CD45RA; BB515	BD	Cat # 564552
Rat Anti-Human CCR7; BV605	BD	Cat # 563711
Mouse Anti-Human CD25; BV510	BD	Cat # 563351
Mouse Anti-Human CD127; AF647	BD	Cat # 560905
Mouse Anti-Human CD3; BV711	BD	Cat # 563724
Rat Anti-Human FOXP3; eFluor450	Thermofisher	Cat # 48-5773-82
Live/Dead; Fixable Red	Thermofisher	Cat # L23102
Mouse Anti-Human CD45; PE/Dazzle 594	Biolegend	Cat # 982308
Mouse Anti-Human CD133; PE/Cyanine 7	Biolegend	Cat # 372810
Mouse Anti-Human CD31; BV605	BD	Cat # 745119
Mouse Anti-Human CD34; APC	BD	Cat # 345804
Mouse Anti-Human VEGFR2; PE	Biolegend	Cat # 359904
Rat Anti-Human SSEA3; PerCP-Cy5.5	BD	Cat # 561564
Mouse Anti-Human CD38; BV711	BD	Cat # 563965
Mouse Anti-Human CD45RA; BB515	BD	Cat # 564552
Mouse Anti-Human CD90; BV510	BD	Cat # 563070
Live/Dead; Fixable Blue	Thermofisher	Cat # L23105
Counting Beads; N/A	Bangs Laboratories	Cat # 580

**Software and algorithms**		

Graphpad Prism	Version 9.0	Graphphad Software
Flowjo	Version 10.7.1	BD
Watson Laboratory Information Management System™	Version 7.2.0.03	Thermo Fisher
Phoenix WinNonlin®	Version 8.0.0.3716	Certara
SAS Grid	Version 9.4	SAS Institute

**Deposited data**		

mRNA Sequencing (PBMCs)	GEO	GSE237965

### Resource availability

#### Lead contact

Further information and requests for recourses and reagents should be directed to the corresponding author, Ali R. Ahmadi (ahmadi@medregenco.com).

#### Materials availability

This study did not generate new unique reagents.

### Experimental model and study participant details

The study was conducted in accordance with Good Clinical Practice as defined by the International Conference on Harmonization and in line with the ethical principles of the Declaration of Helsinki, European Union Directive 2001/20/EC and the US Code of Federal Regulations, Title 21, Part 50. The clinical protocol, amendments, and informed consent forms were approved by the US FDA and by an independent Ethics Committee, IntegReview IRB (Austin, TX, USA), before study initiation and throughout the study. All participants provided written informed consent and could withdraw from the study at will.

#### Study design

This first-in-human, single-center, randomized, double-blind, placebo-controlled, multiple-ascending dose (MAD), phase I study (NCT04646603) in healthy volunteers was performed at the San Antonio Clinical Research Unit, ICON plc, in San Antonio, Texas, in the United States of America between February 2021 and May 2021.

#### Study population

Male and female participants between 18 and 45 years of all ethnicities with a body mass index of 18.8–32 kg/m^2^, and healthy with no clinically significant abnormalities as determined by medical history, physical examination, 12-lead electrocardiogram (ECG), and clinical laboratory evaluations, were eligible for inclusion. All participants were required to have a negative SARS-CoV-2 test by real-time PCR within the previous 96 h before admission.

Non-pregnant, non-lactating females of childbearing potential who agreed to use medically acceptable forms of birth control from the screening visit until the end of the study visit were eligible. Females were required to have a negative serum pregnancy test before admission. Male participants were eligible if they agreed to use effective contraception methods from the signing of the ICF until at least 8 weeks after the last dose of the study drug.

Exclusion criteria were related to a medical history of cardiovascular, respiratory, hepatic, renal, gastrointestinal, endocrine, neurological, immunological, psychiatric disorder(s) and laboratory findings of HIV, hepatitis B, or C. Subjects were excluded if they received concomitant medication that could affect the pharmacokinetics of the investigational product (IP). Other exclusion criteria included subjects unwilling to avoid the use of alcohol within 48 h before screening and for the duration of the study and to abstain from nicotine use from screening until the end of the study. The complete list of inclusion and exclusion criteria is listed in the clinical protocol (Methods S1). A total of 18 subjects were planned to be included in the study.

#### Investigational treatments and dose regimen

MRG-001 is a combination product of Plerixafor (AMD3100) and Tacrolimus (FK506) with excipients for subcutaneous injection. Each 1 mL vial of MRG-001 contains 24 mg/mL Plerixafor and 0.5 mg/mL Tacrolimus. Sterile 0.9% sodium chloride solution for subcutaneous injection served as placebo.

The dose selection was based on established clinical experience with each active pharmaceutical ingredient (API), effective dosages in animal models as well as its safety profile in nonclinical toxicological studies in two species. Subjects were enrolled in 3 sequential cohorts (cohort 1: 0.005 mL/kg, cohort 2: 0.01 mL/kg, cohort 3: 0.02 mL/kg MRG-001 or 0.9% saline placebo) of 6 subjects each, of which 4 subjects were randomized to the MRG-001 group and 2 subjects to the placebo group. Subjects received subcutaneous injections in the abdominal area on days 1, 3 and 5 and were required to fast 1-h pre-dose and 1-h post-dose. Follow-up visits were conducted on days 6 and 7 and subjects were discharged and returned on day 12 for the end-of-study visit.

Each cohort included a sentinel dosing group of 2 subjects (1 MRG-001 and 1 placebo) dosed at the same time, with dosing of the remaining cohort following review of 24 h safety results of both sentinel subjects. Dose escalation to the next cohort occurred after review of all available safety, tolerability and pharmacokinetic data by the data safety review committee consisting of the principal investigator, medical monitor, pharmacokinetics expert and the study sponsor’s physician.

##### Randomization, allocation and masking

A randomization list was generated by SAS software (version 9.4, SAS Institute Inc., USA) with a 1:1 allocation. Prior to dosing, subjects will be assigned a randomization number in accordance with the randomization code. The randomization code was maintained in a room with restricted access to pharmacy personnel only. The randomization code included 3- digit subject numbers starting with 101.Once a randomization number is allocated to one subject, it may not be assigned to another subject. If subjects withdraw prematurely from the study and are replaced, then a replacement randomization number will be assigned. A replacement randomization code will be generated such that replacement subjects are assigned to the same treatment as the discontinued subjects. The replacement randomization code will differ only in randomization numbers, which will be 4-digit numbers starting with a leading 1. In this trial, subjects and the follow-up staff were kept blind to the treatment allocation. The blind was broken after database lock.

#### Sample size determination

Formal sample size calculations were not performed however with a total sample size of 18 healthy volunteers and a given incidence of a specific common AE of 1% in the general population the study would be able to detect an additional incidence of such AE caused by the use of the new drug of 11% with a power of 80%. Given the stage of development and the objective to determine the safety, PK and PD profiles of MRG-001, 12 subjects were considered adequate to assess the safety and PK/PD profiles in this initial part of development.

### Method details

#### Safety assessment

Safety assessments included adverse events (AE) monitoring, vital signs monitoring (systolic and diastolic blood pressure, pulse rate, oral body temperature and respiratory rate), physical examination, concomitant medication assessment, 12-lead electrocardiography and laboratory evaluations (clinical chemistry, hematology, coagulation and urinalysis). The injection site was assessed for erythema, pain, swelling and numerous other parameters according to a standardized local injection site reaction score every 1, 6, 12 and 24 h after injection. The safety of the subjects was assessed until discharge and during the end-of-study visit on day 12.

#### Pharmacokinetic assessment

##### Plerixafor

Blood samples were obtained from all subjects at the following time points: before dosing, at 1, 3, 8, 12, 24 and 48 h after injection. Blood samples were also obtained daily until discharge on day 7. Plasma from each sample was isolated and stored in −80°C condition until shipment. The analysis of plasma for the quantitation of plerixafor using a validated method by liquid chromatography with tandem mass spectrometry (LC-MS/MS) was performed by NorthEast Biolab (Hamden, CT, USA) in compliance with principles of Good Laboratory Practice (GLP) Standards as outlined the US FDA Title 21 CFR Part 58; Good Clinical Practice (US FDA & ICH GCP Guidance) Standards, the Declaration of Helsinki, and the US FDA Guidance for COVID-19 Studies. The data were acquired using Phoenix WinNonlin, Version 8.0.0.3716, Certara, St. Louis, MO, software and the following pharmacokinetic parameters were calculated: peak plasma concentration (C_max_), time to peak plasma concentration (T_max_), terminal half-life (t_1/2_), trough concentration (C_trough_) and area under the plasma concentration-time curve over dosing interval (AUC_tau_).

##### Tacrolimus

Blood samples were obtained from all subjects in parallel as for plerixafor. Whole blood from each sample was stored in −80°C condition until shipment. The analysis of K2EDTA whole blood for the quantitation of tacrolimus using a validated method by LC-MS-MS was performed by Worldwide Clinical Trials, Bioanalytical Services (Austin, TX, USA) in compliance with principles of GLP standards as referenced above.

Study data were collected and similar pharmacokinetic parameters as above were calculated using Phoenix WinNonlin, Version 8.0.0.3716, Certara, St. Louis, MO, software and evaluated with Watson Laboratory Information Management System (LIMS; Version 7.2.0.03, Thermo Fisher Scientific) software.

#### Pharmacodynamics assessment

Blood samples were obtained from certain subjects at pre-dose and at 1, 3, 8, 12, 24 and 48 h for flow cytometry. A stem cell panel and an immune cell panel were developed for this study. Briefly, whole blood was lysed and then washed with PBS. Cells were then stained with a viability dye, washed, and blocked with human Fc block. Surface staining was performed with an extracellular antibody cocktail. For the stem cell panel, after surface staining, the cells were washed and fixed. For the immune cell panel, after surface staining, cells were washed, fixed, and permeabilized. Permeabilized cells were stained with an intracellular antibody cocktail containing FoxP3 antibodies. After intracellular staining, cells were washed and then fixed with stabilizing fixative before acquisition. Counting beads were added to be able to count the absolute number of cells. A list of the antibodies can be found in the key resource table. Data were acquired using a BD LSR Fortessa (BD, San Jose, CA) and analyzed with FlowJo version 10.7.1 and Graphpad Prism version 9.0. At each blood draw timepoint, absolute circulating immune cell or stem cell count, such as CD45^dim^CD34+, was calculated by multiplication of CD45^dim^CD34+ cell fraction of PBMC ((lymphocyte and monocytes in complete blood count (CBC)) by the total circulating PBMC count. Unfortunately, multiple PBMC samples from the 0.005 mL/kg MRG-001 cohort were degraded and unusable for both FACS analysis and RNA sequencing due to logistical delays (winter storm), thus we were unable to include a low-dose cohort for immune and stem cell analysis.

#### RNA purification & next generation sequencing

To understand the molecular mechanisms of MRG-001, next-generation sequencing on RNA isolated from PBMCs was performed and gene expression changes were quantified. Sequencing occurred at multiple time points after injection to define the dynamics of gene expression changes between the different dose groups. Gene expression changes were pooled per timepoint per dose MRG group and compared to the pooled pre-dose gene expression of that specific dose group and to the placebo-treated group.

##### RNA extraction

Samples (snap-frozen white blood cell pellets) were received on dry ice and stored at −80°C until processing commenced. Samples were randomized before extraction. In total, samples from 15 subjects (5 from the placebo group, 2 from 0.005 mL/kg MRG-001, 4 from 0.01 mL/kg MRG-001 and 4 from the 0.02 mL/kg MRG-001 dose group) were sequenced. The Lexogen 008 split RNA extraction kit was used to extract RNA. Samples were characterized by UV-Vis spectrophotometry (Nanodrop2000c, Thermo Fisher), the RNA integrity was assessed on a Fragment Analyzer System using the DNF-471 RNA Kit (15nt) (Agilent).

##### Library preparation

Sequencing-ready libraries were produced after randomization using a QuantSeq 3′ mRNA-Seq Library Prep Kit FWD for Illumina (015UG009V0260) with Globin Block Module following procedures for degraded RNA, as outlined by the manufacturer’s instructions (Lexogen QuantSeQ_Illumina). Indexed library preparation was performed to allow for multiplexed sequencing. For library preparation, 100 ng of extracted RNA samples were used as an input. Prepared libraries were quality controlled and quantified on a Fragment Analyzer using HS NGS Fragment Kit(1-6000bp). A sequencing-ready pool of indexed libraries was prepared according to these quantifications.

##### Sequencing

Sequencing was performed on an Illumina NextSeq2000 with a 100cyc P2 flow cell sequencing kit at Lexogen GmbH.

##### Sequencing quality control and adapter trimming

Using cutadapt version 1.18, the reads of the sequencing run were scanned for adapter contaminations, continuous polyA sequences and continuous polyG sequences at the 3′ end and had the contaminations removed if they were found. The reads of the samples prior to adapter trimming and after adapter trimming were analyzed with FastQC version v0.11.7.

##### Alignment and read quantification

The reads were aligned to the spike-in complemented Ensembl release 94 of the Homo sapiens assembly GRCh38 from the Genome Reference Consortium. The alignment was performed with the splice-aware aligner STAR version 2.6.1a. The alignments were quantified based on the annotations of Ensembl GRCh38.94 and the spike-in-specific annotations of Lexogen with the featureCounts software program version 1.6.4 of the subread analysis package.

#### Differential gene expression analysis

A differential gene expression analysis was conducted using DESeq2 (version v1.18.1). The analysis used the counts of unique alignments. Significance was determined at adjusted p < 0.1.

##### Functional enrichment analysis

For gene set enrichment testing the hallmark dataset of the molecular signature database has been used. The data used has been retrieved from the CRAN R package msigdbr 7.5.1. This is visualized in a ridgeline plot, where the density of these significantly enriched gene sets is plotted against their log-fold changes.

### Quantification and statistical analysis

All statistical tabulations and analyses were done using SAS Grid/SAS Linux: SAS 9.4. Formal statistical tests were conducted at a 2-sided 5% significance level.

Disposition, demographics, baseline characteristics and all safety parameters were summarized by cohort and treatment. Placebo was pooled across cohorts for summary and analysis. The PK full set includes all subjects who received at least 1 dose of study treatment and have at least 1 plasma concentration data point. The PD set will include those subjects in the safety analysis set who have pre-dose and at least 1 of the post-dose PD parameter concentrations and have no events or deviations that would affect PD variables.

All descriptions of Materials and Methods should be included in the main paper. The Materials and Methods should be broken up into sections, each with a short subheading. Please include a study design paragraph at the start of the Materials and Methods and a statistical paragraph at the end of the Materials and Methods in the main text. If the Materials and Methods make the paper exceed the length limitations, less important sections of the Materials and Methods can be moved to the Supplementary Materials.

### Additional resources

The study was registered on clinicaltrials.gov (NCT04646603) as part of a combined Phase I/IIa protocol for the treatment of severely and critically ill COVID-19 patients. Details of Part A of the study can be viewed in the accompanied clinical protocol (Methods S1).

## Data Availability

• The mRNA sequencing data supporting the findings of this study from all of the samples have been deposited in the Gene Expression Omnibus (GEO) database (http://www.ncbi.nlm.nih.gov/geo) and are publicly available at the date of publication (GSE237965). Accession numbers are listed in the key resources table. • This paper does not report original code. • Any additional information required to reanalyze the data reported in this paper is available from the lead contact upon request.

## Supplemental information

Document S1. Tables S1–S5, Figures S1–S3, and Methods S1

Document S2. Article plus supplemental information
