# Supplementary material for: A phase I, first-in-human study to evaluate the safety and tolerability, pharmacokinetics, and pharmacodynamics of MRG-001 in healthy subjects
Source: Cell Rep Med. 2023 Aug 25;4(9):101169. doi: 10.1016/j.xcrm.2023.101169 (PMC10518600; doi:10.1016/j.xcrm.2023.101169)
Supplement: Document S1. Tables S1–S5, Figures S1–S3, and Methods S1 [file mmc1.pdf]

Cell Reports Medicine, Volume *4*

Supplemental information

A phase I, first-in-human study to evaluate the safety and tolerability, pharmacokinetics, and pharmacodynamics of MRG-001 in healthy subjects

Ali R. Ahmadi, George Atiee, Bart Chapman, Laurie Reynolds, John Sun, Andrew M. Cameron, Russell N. Wesson, James F. Burdick, and Zhaoli Sun

Table S1. Summary of Plerixafor Pharmacokinetic Parameters on Days 1 and 5. Related to Figure 1.

Parameter (unit)	Statistic	Cohort 1 MRG-001 0.005 mL/kg		Cohort 2 MRG-001 0.01 mL/kg		Cohort 3 MRG-001 0.02 mL/kg	
		Day 1 (N = 4)	Day 5 (N = 4)	Day 1 (N = 5)	Day 5 (N = 4)	Day 1 (N = 4)	Day 5 (N = 4)
C_{max} (ng/mL)	n	4	4	4	4	4	4
	GM	274	302	601	613	947	1115
	GeoCV%	15.3	11.0	11.9	21.0	8.65	7.49
T_{max} (h)	n	4	4	4	4	4	64
	Median	2.00	1.00	3.00	1.00	2.00	1.00
	Min, Max	1.00, 3.00	1.00, 3.00	1.00, 3.00	2.01, 3.05	1.00, 3.00	1.00, 3.00
AUC_{tau} (ng*h/mL)	n	2	3	4	4	4	4
	GM	1598	1846	4171	4336	7325	8916
	GeoCV%	1.69	14.9	5.48	13.5	19.1	22.9
$t_{1/2}$ (h)	n	2	3	4	4	4	4
	GM	3.29	4.56	4.26	4.56	5.01	6.06
	GeoCV%	1.16	3.38	13.3	3.38	17.8	28.8

Abbreviations: AUC = area under the plasma concentration time curve; AUC_{tau} = AUC over 1 dosing interval; C_{max} = maximum observed plasma concentration; GeoCV% = geometric percent coefficient of variation; GM = geometric mean; max = maximum; min = minimum; n = number of non-missing observations; N = number of subjects in respective category; PK = pharmacokinetic; $t_{1/2}$ = terminal elimination half-life; T_{max} = time of maximum observed plasma concentration.

Table S2. Summary of Tacrolimus Pharmacokinetic Parameters on Days 1 and 5. Related to Figure 1.

Parameter (unit)	Statistic	Cohort 1 MRG-001 0.005 mL/kg		Cohort 2 MRG-001 0.01 mL/kg		Cohort 3 MRG-001 0.02 mL/kg	
		**Day 1 (N = 4)**	**Day 5 (N = 4)**	**Day 1 (N = 5)**	**Day 5 (N = 4)**	**Day 1 (N = 4)**	**Day 5 (N = 4)**
C_{max} (ng/mL)	n	4	4	5	4	4	4
	GM	1.47	2.09	3.15	5.37	4.49	7.91
	GeoCV%	34.7	11.3	18.0	11.2	28.8	14.2
T_{max} (h)	n	4	4	5	4	4	64
	Median	7.50	10.00	12.00	5.50	8.01	2.00
	Min, Max	3.00, 12.00	1.00, 12.03	1.00, 12.02	1.00, 12.00	3.00, 24.00	1.00, 24.00
AUC_{tau} (ng*h/mL)	n	4	4	5	4	4	4
	GM	46.4	70.1	105	171	152	237
	GeoCV%	10.8	13.1	14.7	19.7	23.0	28.0
$t_{1/2}$ (h)	n	2	2	2	3	3	3
	GM	23.8	24.9	37.8	29.5	36.4	30.6
	GeoCV%	25.2	6.11	69.2	43.9	33.8	22.5
C_{trough} (ng/mL)	n	4	4	4	4	4	4
	GM	0.57	0.88	1.45	1.89	2.12	2.94
	GeoCV%	26.07	26.19	26.80	41.56	38.77	47.25

Abbreviations: AUC = area under the plasma concentration time curve; AUC_{tau} = AUC over 1 dosing interval; C_{max} = maximum observed plasma concentration; GeoCV% = geometric percent coefficient of variation; GM = geometric mean; max = maximum; min = minimum; n = number of non-missing observations; N = number of subjects in respective category; PK = pharmacokinetic; $t_{1/2}$ = terminal elimination half-life; T_{max} = time of maximum observed plasma concentration; C_{trough} = through concentration.

Table S3. Changes in Gene Expression and Molecular Pathways – MRG-001 versus Placebo. Related to Figure 5.

	Down-regulated genes						Up-regulated genes					
Dosage	1h	3h	8h	12h	24h	48h	1h	3h	8h	12h	24h	48h
Placebo	0	0	0	0	0	0	0	0	0	0	0	0
0.005mL/kg	5	0	0	0	2	0	1	1	1	0	0	0
0.01mL/kg	0	0	850	1474	270	0	0	0	1494	825	18	0
0.02mL/kg	66	0	7	484	202	70	25	1	32	202	55	8
	Down-regulated pathways						Up-regulated pathways					
Dosage	1h	3h	8h	12h	24h	48h	1h	3h	8h	12h	24h	48h
Placebo	0	0	0	0	0	0	0	0	0	0	0	0
0.005mL/kg	0	0	0	0	0	0	0	0	0	0	0	0
0.01mL/kg	0	0	17	24	19	0	0	0	0	0	0	0
0.02mL/kg	0	0	4	9	5	8	0	0	1	0	1	1

Table S4. Changes in Gene expression and Molecular Pathways – MRG-001 versus Pre-dose. Related to Figure 5.

	Down-regulated genes						***Up-regulated genes***					
Dosage	1h	3h	8h	12h	24h	48h	1h	3h	8h	12h	24h	48h
Placebo	0	0	0	0	0	0	0	0	0	0	0	0
0.005mL/kg	5	0	0	0	2	0	1	0	0	0	0	0
0.01mL/kg	0	0	654	630	199	0	0	2	1038	225	9	1
0.02mL/kg	0	0	15	132	78	0	0	0	29	72	43	0
	Down-regulated pathways						***Up-regulated pathways***					
Dosage	1h	3h	8h	12h	24h	48h	1h	3h	8h	12h	24h	48h
Placebo	0	0	0	0	0	0	0	0	0	0	0	0
0.005mL/kg	0	0	0	0	0	0	0	0	0	0	0	0
0.01mL/kg	0	0	20	19	19	0	0	0	0	1	0	0
0.02mL/kg	0	0	0	4	4	0	0	0	0	0	0	0

Table S5. Differential Regulated Common Pathways by MRG-001: high dose (0.02mL/kg) versus intermediate dose (0.01mL/kg). Related to Figure 5.

	Hours post MRG injection			
0.02mL/kg versus 0.01mL/kg	8	12	24	48
Down regulated pathways (2)				
MYC Targets V1			yes	
MYC Targets V2			yes	
Up regulated pathways (5)				
TNFA Signaling via NFKB			yes	
Inflammatory response			yes	
Interferon Gamma Response			yes	
Hypoxia			yes	
Complement			yes	

Figure S1. Regulatory T-cell/ T-cell Ratios. Related to Figure 3.

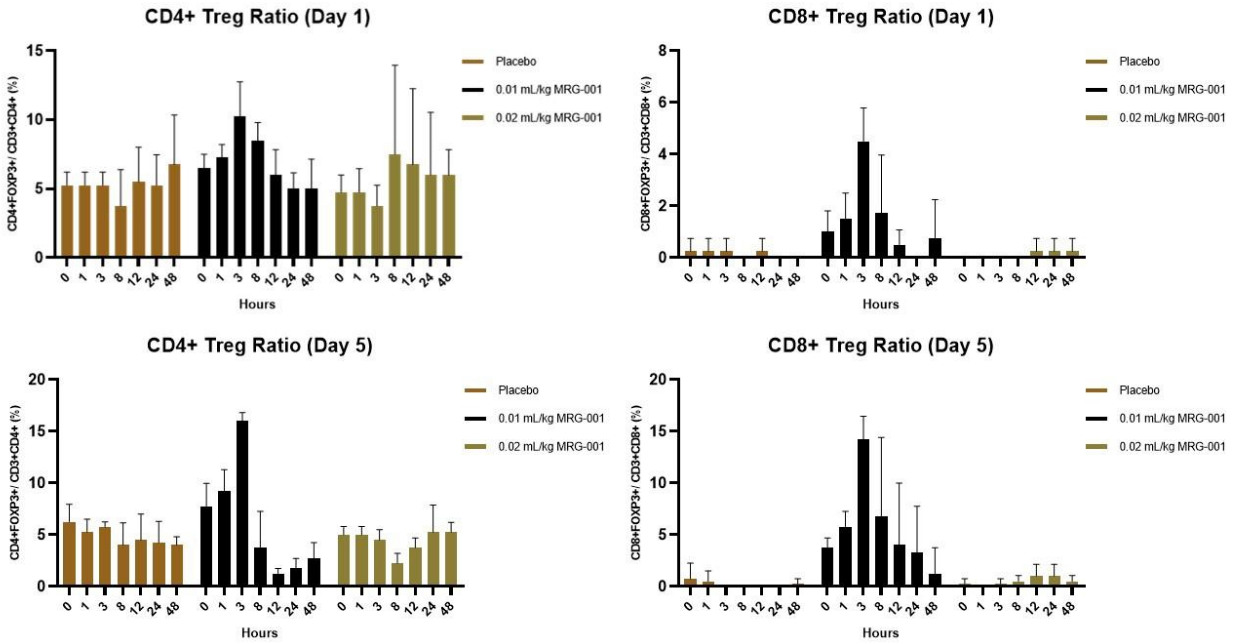

The ratios between regulatory T-cells/ T-cells are calculated for the different groups during the course of mobilization for day 1 and day 5. Especially the ratios between CD4FOXP3+ versus CD4+ cells are calculated and the ratios between CD8+FOXP3+ versus CD8+ cells. The most significant changes in the increase of the ratio are noticed in the intermediate (0.01 mL/kg) dose group.

Figure S2. Venn diagram analysis of molecular pathways down-regulated by MRG-001 at 8, 12 and 24 hours post injection compared to pre-dose. Related to Figure 5.

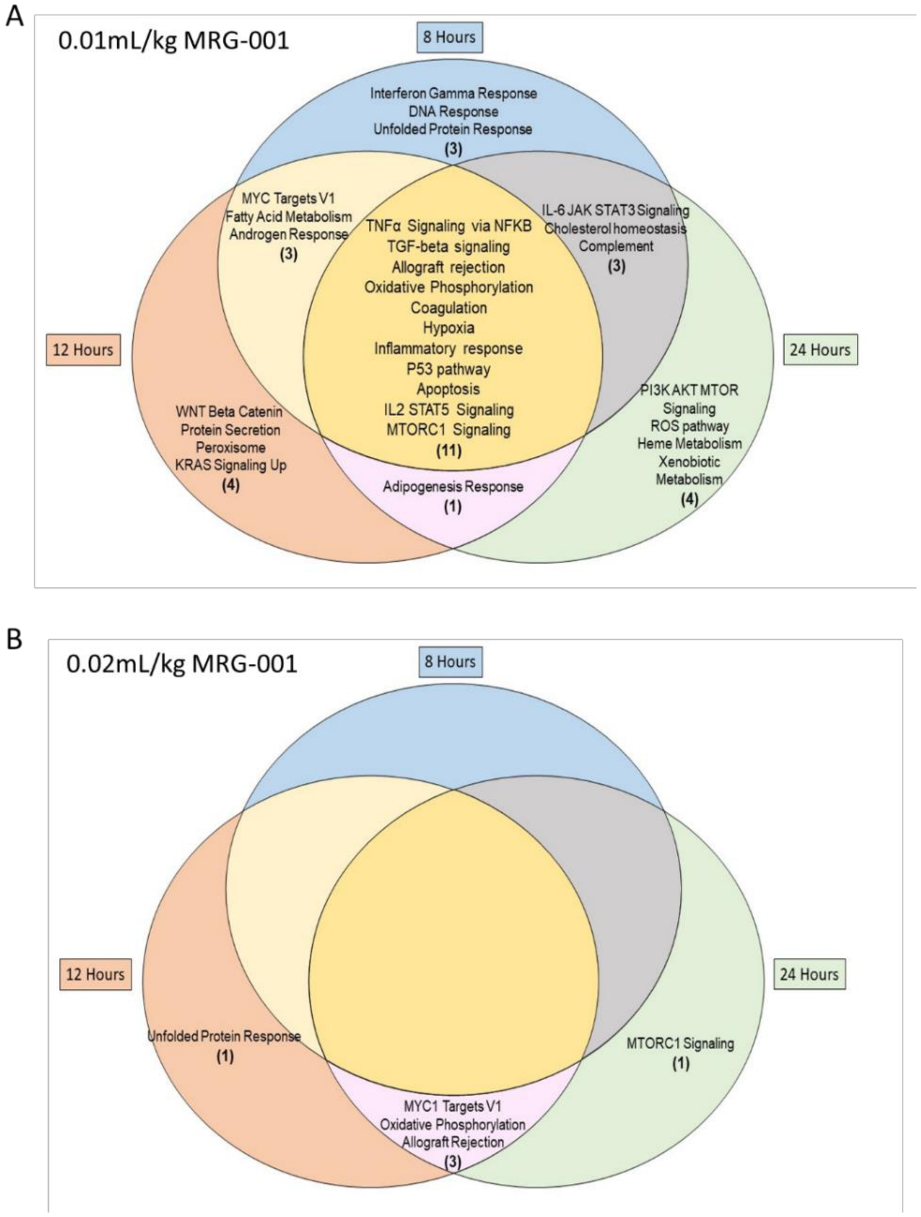

(**A**) 0.01mL/kg MRG-001. Compared to pre-dose, down-regulated pathways at 8 hours, 12 hours and 24 hours are 20, 19 and 19, respectively. Total of 29 pathways are down-regulated. The shared down-regulated pathways at different time points were analyzed using the Venn diagram analysis. As illustrated in each panel, the common down-regulated pathways at different time points are 11. (**B**) 0.02mL/kg MRG-001. Compared to pre-dose, down-regulated pathways at 8, 12 and 24 hours are 0, 4 and 4, respectively. Total 5 pathways are down regulated, and shared down-regulated pathways at 12 and 24 hours are 3.

Figure S3. Venn diagram analysis of common down-regulated pathways between MRG-001 vs Placebo and MRG-001 vs Pre-dose. Related to Figure 5.

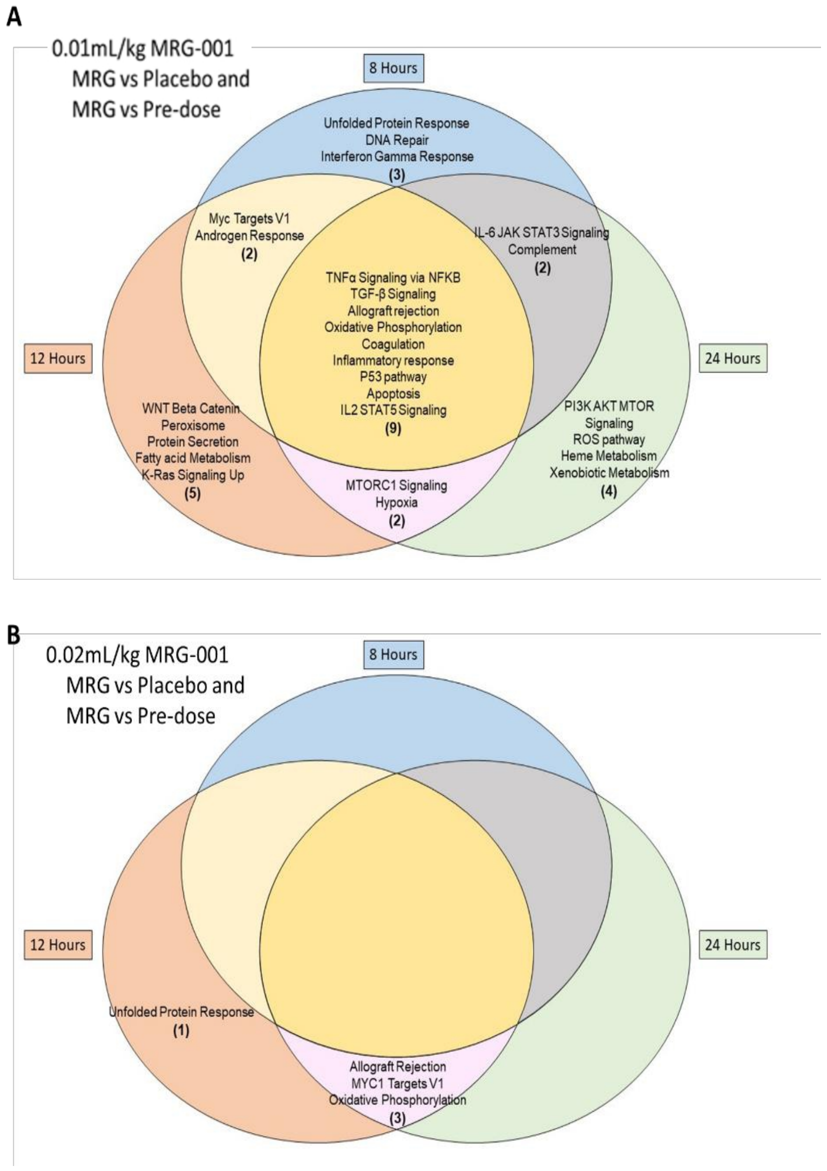

(**A**) 0.01mL/kg MRG-001. Common down-regulated pathways between 0.01mL/kg MRG-001 versus Placebo and 0.01mL/kg MRG-001 versus Pre-dose at 8, 12 and 24 hours are 16, 18 and 17, respectively. Total common down-regulated pathways are 27. As illustrated in each panel, the shared common down-regulated pathways at different time points are 9. (**B**) 0.02mL/kg MRG-001. Common down-regulated pathways between 0.02mL/kg MRG-001 versus Placebo and 0.02mL/kg MRG-001 versus Pre-dose at 8, 12 and 24 hours are 0, 4 and 3, respectively. Total common down-regulated pathways are 4. As illustrated in each panel, the shared common down-regulated pathways at 12 and 24 hours are 3.

Methods S1. Clinical Trial Protocol

Clinical Study Protocol

A Combined Phase I Double-Blind Randomized Placebo-controlled Study in Healthy Subjects/Phase IIa, Randomized, Double-blind, Placebo-controlled, Multi-center Study in Subjects Infected with SARS-CoV-2 to Assess the Safety, Pharmacokinetics, Pharmacodynamics and Efficacy of MRG-001 (Plerixafor Plus Low-dose Tacrolimus)

Sponsor:	Medregen, LLC<br>855 N Wolfe St., Suite 623.3<br>Baltimore, MD 21205, USA
Contract Research Organization:	ICON Early Phase Services<br>8307 Gault LN<br>San Antonio, TX, 78209, USA<br>[redacted]
Principal Investigator:	George J. Atiee, MD
Sponsor Study Number:	MRG2020
PIND	[redacted]
IMP Name:	MRG-001
Development Phase:	1/2, safety and pharmacokinetics
Version (Date) of Final Protocol:	29 January 2021

This clinical study will be conducted in accordance with the International Council for Harmonisation Tripartite Guideline for Good Clinical Practice (GCP) E6 (R2), the protocol and with other applicable regulatory requirements.

Confidentiality Statement

This document contains confidential information of Medregen, LLC. Do not copy or distribute without written permission from the Sponsor.

SIGNATURE PAGE

Declaration of Sponsor or Responsible Medical Expert

Protocol Title: A Combined Phase I Double-blind Randomized Placebo-controlled Study in Healthy Subjects/Phase IIa, Randomized, Double-blind, Placebo-controlled, Multi-center Study in Subjects Infected with SARS-CoV-2 to Assess the Safety, Pharmacokinetics, Pharmacodynamics and Efficacy of MRG-001 (Plerixafor Plus Low-dose Tacrolimus)

This clinical study protocol was subjected to critical review. The information it contains is consistent with current knowledge of the risks and benefits of the investigational medicinal product (IMP), as well as with the moral, ethical and scientific principles governing clinical research as set out in the guidelines on GCP applicable to this clinical study.

Sponsor Signatory/Responsible Medical Expert

[redacted]

SIGNATURE PAGE

Declaration of the Principal Investigator

Protocol Title: A Combined Phase I Double-blind Randomized Placebo-controlled Study in Healthy Subjects/Phase IIa, Randomized, Double-blind, Placebo Controlled, Multi-center Study in Subjects Infected with SARS-CoV-2 to Assess the Safety, Pharmacokinetics, Pharmacodynamics and Efficacy of MRG-001 (Plerixafor Plus Low-dose Tacrolimus)

This clinical study protocol was subjected to critical review and has been released by the Sponsor. The information it contains is consistent with current risk and benefit evaluation of the IMP, as well as with the moral, ethical and scientific principles governing clinical research as set out in the guidelines on GCP applicable to this clinical study.

Principal Investigator

Dr George J. Atiee, MD<br>Medical Director<br>ICON Early Phase Services	05Feb2021<br>Date

LIST OF STUDY STAFF

**Sponsor**	Medregen, LLC<br>855 N Wolfe St., Suite 623.3<br>Baltimore, MD 21205, USA<br>Telephone number: +1 800 674 8175<br>Fax number: +1 877-674-1877<br>Email: info@medregenco.com
**Principal Investigator**	George J. Atiee, MD
**Contract Research Organization (Regulatory services and data management)**	ICON Clinical Research, LLC<br>820 West Diamond Avenue, Suite 100<br>Gaithersburg, MD 20878<br>[redacted]
**Study Site**	ICON Early Phase Services<br>8307 Gault LN<br>San Antonio, TX, 78209, USA<br>[redacted]
**Adverse Event Reporting**	Medical Assistance and Serious Adverse Event Reporting<br>ICON Clinical Research, LLC<br>Fax number: +1 215 616 3096<br>Email: icon-mads@iconplc.com
**Medical Monitor**	Dr Bart Chapman, MD<br>[redacted]
**Clinical Laboratory**	ICON Early Phase Services<br>8307 Gault Lane | San Antonio, Texas, 78209, USA<br>[redacted]
**Bioanalytical Laboratory (Plerixafor)**	[redacted]
**Bioanalytical Laboratory (Tacrolimus)**	[redacted]
**Flow Cytometry Laboratory**	[redacted]

**Biostatistician**	ICON Clinical Research, LLC<br>Gaithersburg, MD 20878
**Medical Writer**	[redacted]<br>Parexel International, UK

TABLE OF CONTENTS

SIGNATURE PAGE ... 2
SIGNATURE PAGE ... 3
LIST OF STUDY STAFF ... 4
TABLE OF CONTENTS ... 6
List of Tables ... 9
List of Figures ... 9
LIST OF ABBREVIATIONS AND DEFINITION OF TERMS ... 10
1. PROTOCOL SUMMARY ... 13
1.1. Protocol Synopsis ... 13
1.2. Schema ... 19
1.2.1. Schedule of Assessments, Time Points and Window Allowance ... 19
2. INTRODUCTION ... 28
2.1. Background ... 28
2.1. Pathophysiology of severe acute respiratory syndrome-coronavirus-2 infection ... 28
2.1.1. Lymphopenia and cytokine storm ... 29
2.1.2. Pulmonary injury and fibrosis ... 29
2.1.3. Endothelial cell injury ... 30
2.1.4. Other Organ Pathologies ... 30
2.2. Clinical management of severe acute respiratory syndrome-coronavirus-2 ... 31
2.3. Summary of Findings from Non-clinical Studies with Potential Clinical Relevance ... 33
2.3.1. MRG-001 in a Mouse Model of *Strep. Pneumoniae* Induced ARDS ... 34
2.3.2. MRG-001 in Other Animal Models of Organ Injury ... 35
2.4. Summary of Findings from Previous Clinical Studies ... 36
2.5. Rationale for the Clinical Study ... 36
2.6. Risk-benefit Assessment ... 36
3. STUDY OBJECTIVES AND ENDPOINTS ... 39
3.1. Part A ... 39
3.1.1. Primary Objective ... 39
3.2. Part B ... 39
3.2.1. Primary Objective ... 39
3.3. Endpoints ... 39
3.3.1. Part A ... 39
3.3.2. Part B ... 40
4. STUDY DESIGN ... 43
4.1. Overview ... 43
4.2. Scientific Rationale for the Study Design ... 47

4.3. Justification for Dose 47
4.4. Order of Assessments 50
4.5. Study Duration 50
4.6. Study Completion 51
4.7. Early Termination 51
5. STUDY POPULATION 52
5.1. Number of Subjects 52
5.2. Inclusion Criteria 53
5.3. Exclusion Criteria 54
5.4. Restrictions 57
5.4.1. Activity 57
5.4.2. Dietary and Fluid Restrictions 57
5.4.3. Lifestyle Considerations 58
5.4.4. Medication 58
5.4.5. Contraception Rules 58
6. INVESTIGATIONAL MEDICINAL PRODUCT 62
6.1. Identity of the Investigational Medicinal Products 62
6.2. Supply, Packaging, Labeling and Storage 62
6.3. Drug Accountability, Dispensing and Destruction 62
6.4. Subject Identification and Randomization 63
6.4.1. Screening Numbers 63
6.4.2. Randomization numbers 63
6.5. Administration of Investigational Medicinal Products 63
6.5.1. Part A 63
6.5.2. Part B 64
6.6. Compliance 65
6.7. Blinding and Breaking the Blind 65
6.8. Treatment of Overdose 66
7. DISCONTINUATION 67
7.1. Dose Escalation and Study Stopping Rules 67
7.1.1. Data Safety Review Committee 67
7.1.2. Stopping Rules 68
7.2. Subject Withdrawal and Replacement 72
7.3. Eligibility Screening 73
7.4. Medical History, Demographic and Other Baseline Information 75
7.5. Safety Variables 75
7.5.1. Adverse Events 75
7.5.2. Clinical Laboratory Assessments 82
7.5.3. Vital Signs 84
7.5.4. Standard 12-lead Electrocardiograms 84
7.5.5. Physical Examinations 84

7.5.6. Injection Site Monitoring ... 85
7.6. Pharmacokinetics Variables ... 85
7.6.1. Blood Sample Collection ... 85
7.7. Pharmacodynamics Variables ... 85
7.8. Total Amount of Blood ... 86
8. STATISTICAL CONSIDERATIONS ... 87
8.1. Sample Size Considerations ... 87
8.2. Study Population ... 88
8.2.1. Disposition of Subjects ... 88
8.2.2. Protocol Deviations ... 88
8.2.3. Analysis Populations ... 88
8.3. General Considerations ... 89
8.4. Protocol Deviations ... 89
8.5. Subject Disposition ... 89
8.6. Demographic and Anthropometric Information and Baseline Characteristics... 89
8.7. Prior and Concomitant Medication and Drug Administration ... 90
8.8. Exposure ... 90
8.9. Efficacy Analyses ... 90
8.10. Safety Analyses ... 91
8.10.1. Adverse Events ... 91
8.10.2 Clinical Laboratory Tests ... 91
8.10.3 Vital Signs ... 91
8.10.4 Standard 12-lead Electrocardiogram ... 92
8.10.5 Physical Examination ... 92
8.11. Pharmacokinetics Analyses ... 92
8.12. Pharmacodynamics Analyses ... 92
8.13. Interim Analyses ... 92
9 ETHICAL, LEGAL AND ADMINISTRATIVE ASPECTS ... 93
9.10 Data Quality Assurance ... 93
9.11 Data collection and Access to Source Data/Documents ... 93
9.12 Archiving Study Documents ... 93
9.13 Good Clinical Practice ... 94
9.14 Informed Consent ... 94
9.15 Insurance and Compensation for Injury ... 95
9.16 Protocol Approval and Amendment(s) ... 95
9.17 Confidentiality Data Protection ... 95
9.18 Publication Policy ... 96
10. REFERENCE LIST ... 97
11 APPENDICES ... 102
Appendix 5 Local Injection Site Reaction Score ... 107

List of Tables

Table 1 Part A Schema 19
Table 2 Part B Schema 19
Table 3 Schedule of Assessments (Part A) 20
Table 4 Schedule of Assessments (Part B) 24
Table 5 Study Design 43
Table 6 Estimated Safety Margins in Rats and Mini-Pigs Employed in the Repeat-Dose Toxicology Study 49
Table 7 Identity of Investigational Products 62
Table 8 Assessment of Relationship of Adverse Events to Investigational Product 79
Table 9 Clinical Laboratory Assessments 83
Table 10 Part A: Approximate Total Amount of Blood for Each Subject 86
Table 11 Part B: Approximate Total Amount of Blood for Each Subject 86
Table 12 Sample Sizes for Different Treatment Effects with Power 80%, One-sided Alpha=0.025 for Time to Improvement from Randomization to Day 28 by Two Points on the 8-point World Health Organization Ordinal scale 87
Table 13 National Institutes of Health Clinical and Physiological Characteristics of COVID-19 Severity 106

List of Figures

Figure 1 Study Flow Chart (Part A) 45
Figure 2 Study Flow Chart (Part B) 46
Figure 3 Standardized Injection Sites for Study Drug Injection 64

LIST OF ABBREVIATIONS AND DEFINITION OF TERMS

Abbreviation	Definition
ACE2	Angiotensin-converting enzyme 2
AE	Adverse event
ALT	Alanine aminotransferase
ALP	Alkaline Phosphatase
AMD3100	Plerixafor
API	Active pharmaceutical ingredient
ARDS	Acute respiratory distress syndrome
AST	Aspartate aminotransferase
BiPAP	Bilevel positive airway pressure
BMP	Bone morphogenetic protein
BUN	Blood urea nitrogen
CBC	Complete blood count
CD133+	Cluster of differentiation 133
CK	Creatinine Kinase
COPD	Chronic obstructive pulmonary disease
COVID-19	Corona virus disease 2019
CMP	Comprehensive metabolic panel
CPAP	Continuous positive airway pressure
CrCl	Creatinine clearance
CRP	C-reactive protein
CT	Computed tomography
CXCR4	C-X-C motif chemokine receptor 4
ECG	Electrocardiogram
ECMO	Extracorporeal membrane oxygenation
eGFR	Estimated Glomerular Filtration Rate
FDA	Food and Drug Administration
FiO_2	Inspired oxygen fraction
FK506	Tacrolimus
FKBP	FK506 binding protein
FKBP1A	FKBP prolyl isomerase 1A
FKBP1B	FKBP prolyl isomerase 1B

Foxp3+	Forkhead box P3
eGFR	Estimated glomerular filtration rate
GFP	Green fluorescent protein
GLP	Good laboratory practice
HCA	Hospital Corporation of America
HCoV-229E	Human coronavirus 229E
HCoV-NL63	Human coronavirus NL63
HR	Heart rate
IBD	Inflammatory bowel disease
ICU	Intensive care unit
IRB	Institutional review board
ITT	Intent-to-Treat
IV	Intravenous
LDH	Lactate dehydrogenase
LPS	Lipopolysaccharide
mITT	Modified Intent-to Treat
MRG-001	Plerixafor and low-dose tacrolimus
mSOFA	Modified sequential organ failure assessment score
MV	Mechanical ventilation
NIH	National Institutes of Health
NEWS	National Early Warning Score
PAH	Pulmonary artery hypertension
PCR	Polymerase chain reaction
PK	Pharmacokinetics
PP	Per Protocol
QOD	Every other day
RR	Respiratory rate
RT-PCR	Reverse transcriptase – polymerase chain reaction
SARS-CoV-2	Severe acute respiratory syndrome-related coronavirus
SAE	Serious adverse event
SC	Subcutaneous
SDF1	Stromal cell derived factor 1
sIL-6R	Soluble interleukin 6 receptor

SpO_2	Peripheral capillary oxygen saturation
TBili	Total bilirubin
TEAEs	Treatment emergent adverse events
TNF	Tumor necrosis factor

1. PROTOCOL SUMMARY

1.1. Protocol Synopsis

**Protocol Title**	A Combined Phase I Double-blind Randomized Placebo-controlled Study in Healthy Subjects/Phase IIa, Randomized, Double-blind, Placebo-controlled, Multi-center Study to Assess the Safety, Pharmacokinetics, Pharmacodynamics and Efficacy of MRG-001 (Plerixafor Plus Low-dose Tacrolimus) in Subjects Infected with SARS-CoV-2.
**Study Numbers**	Sponsor Protocol No.: MRG2020
**Development Phase**	I/IIa,
**Sponsor**	Medregen, LLC
**Principal Investigator**	George J. Atiee, MD
**Study Center**	Part A of the study will be conducted at a single center in the United States (US); Part B of the study will be conducted at 2 sites in 2 centers in the US.
**Study Objectives**	The study will consist of 2 parts:<br>*Part A*<br>*Primary Objective:*<br>To determine the safety and tolerability, pharmacokinetics (PK) and pharmacodynamics (PD) profiles of MRG-001 in healthy subjects.<br>*Part B*<br>[redacted].
**Study Design**	This study is a randomized, double-blind, placebo controlled, Phase I initial safety study in adult male and female subjects in a single center followed by a randomized, double-blind, placebo-controlled, multi-center study Phase IIa study in male and female subjects with moderate-to-severe COVID-19.<br>The study will consist of 2 parts:<br>Part A in 18 healthy subjects: 3 cohorts of 6 subjects (4 active and 2 placebo) each;<br>[redacted].<br>Part A:<br>Part A will consist of a Screening Visit (Day -14 to -1), a Treatment Period (Day 1 to 5), follow-up visits (Days 6 and 7), and End-of-Study (EOS) Visits at Day 12.<br>Three dose levels will be planned for subjects in Part A: 0.005 mL/kg, 0.01 mL/kg and 0.02 mL/kg MRG-001. Subjects will be enrolled in 3 sequential cohorts (Cohort 1, Cohort 2 and Cohort 3) each consisting of 6 healthy subjects for a total of 18 subjects. Subjects will visit the clinical unit (CU) on Day 1 to receive the study drug (MRG-001 or placebo) subcutaneously (SC). Subjects will remain at the CU for 24 hours for observation and assessment. Subjects will return to the CU to repeat the same procedures every other day (QAD) for 5 days. The initial dose level will be 0.005 mL/kg based on nonclinical study results. The first cohort will include a sentinel group (Cohort 1a: first 2 subjects with 1 per treatment) dosed at the same time. Dose administration

	for the remainder of the first cohort (Cohort 1b) will occur only at least 24 hours after the 2 sentinel subjects received study drug (MRG-001 or placebo) and will be contingent upon acceptable safety results through 24 hours for the sentinel subjects per Investigator in consultation with the Sponsor and with explicit agreement from the Sponsor.<br>Progression to and the dose level for the next cohort (Cohort 2) will be dependent upon safety data through Day 5 from the previous cohort.<br>The second cohort will receive 0.01 mL/kg MRG-001 or placebo. The cohort will include a sentinel group (Cohort 2a: first 2 subjects with 1 MRG-001 and 1 placebo) dosed at the same time. Dose administration for the remainder of the second cohort (Cohort 2b) will occur only at least 24 hours after the 2 sentinel subjects received study drug and will be contingent upon acceptable safety results through 24 hours for the sentinel subjects per Investigator in consultation with the Sponsor and with explicit agreement from the Sponsor.<br>Progression to the dose level for the next cohort (Cohort 3) will be dependent upon safety data through Day 5 from the previous cohort.<br>The third cohort will receive 0.02 mL/kg MRG-001 or placebo. The cohort will include a sentinel group (Cohort 3a: 2 subjects; 1 MRG-001 and 1 placebo) dosed at the same time. Dose administration for the remainder of the subjects in the second cohort (Cohort 3b) will occur only at least 24 hours after the 2 sentinel subjects received study drug and will be contingent upon acceptable safety results through 24 hours for the sentinel subjects per Investigator in consultation with the Sponsor and with explicit agreement from the Sponsor.<br>Subjects will return to the clinical unit and receive additional doses of MRG-001 or placebo on Days 3 and 5. Follow-up assessments for safety and PK will be scheduled on Days 6 and 7 and an end of study safety assessment on Day 12.<br>The Data Safety Review Committee (DSRC) will review all available safety, tolerability and if feasible and available PK and PD data prior to making a decision to escalate to the next cohort.<br>The estimated duration of study participation (Screening through EOS Visit [Day 12]) for an individual subject in Part A will be approximately four weeks.<br>[redacted]

	[redacted]
**Investigational Medicinal Product**	MRG-001 or placebo, multiple doses (total of 3 doses), SC, QAD for up to 5 days in healthy adult subjects [redacted]
**Number of Subjects**	[redacted]: 18 healthy volunteer subjects [redacted] will be enrolled in the clinical study according to the inclusion/exclusion criteria.
**Diagnosis and Main Criteria for Inclusion and Exclusion:**	Part A: healthy subjects, 18 to 45 years of age with a body mass index (BMI) between 18.8 and 32.0 kg/m^2, are planned for enrollment.<br><br>Nonsmokers (or other nicotine use) whose clinical status is healthy with no limitation in activities and a negative laboratory-confirmation of SARS-CoV-2 by real time transcription polymerase chain reaction in the respiratory tract (nasopharyngeal [NP] swab) within the previous 96 hours.<br><br>Subjects were to be in good health with no clinically significant abnormalities as determined by medical history, physical examination, 12-lead ECG and clinical laboratory tests prior to admission.<br><br>[redacted]<br><br>In both Parts A and B, subjects will be excluded if they received concurrent treatment with other agents with actual or possible direct acting immunomodulatory activity against ARDS in COVID-19 <72 hours prior to study drug dosing, had splenomegaly (spleen weighing >750 g), had a history of cancer or thrombocytopenia (platelet count <100,000/µL) or thrombocythemia (platelet count >500,000/µL), known family history of long

	QT syndrome (Torsades de Pointes) or currently taking medication that prolongs QT interval, Serum ALP or BIL 1.5 x ULN or ALT or AST > ULN at either screening or admission, a creatinine clearance <50 mL/min using the Cockcroft-Gault formula, a history of hypersensitivity to MRG-001 (plerixafor [AMD3100, 24 mg/mL]) and tacrolimus [FK506, 0.5 mg/mL]) or any of the excipients or to medicinal products with similar chemical structures.
**Criteria for Evaluation**	Part A:<br>*Primary Endpoint*<br>• Change from baseline in the proportion of subjects experiencing any treatment emergent adverse event (TEAE) associated with MRG-001 to Day 12.<br>*Secondary Endpoints:*<br>• Change in percentages from baseline in circulating stem cell and lymphocyte subpopulations concentrations.<br>Stem cell panel: CD3, CD19/20, CD33, CD14, CD56, CD16) / CD45 / CD45RA/ CD38/ CD90 / CD133 / CD31 / CD34 / VEGFR2 / SSEA3 / Live-dead<br>Immune cell panel: CD3 / CD4 / CD8 / CD19 / CD56 / CD16 / CD45RA / CCR7 / CD25 / CD127 / FOXP3 / ICOS / CTLA4 / Live-dead<br>• Change from baseline in white blood cell count, hemoglobin, platelet count to Day 12.<br>• Change from baseline in ALT, AST, tBili, LDH, CK, creatinine, BUN, eGFR to Day 12.<br>*Pharmacokinetics Endpoints*<br>The following PK parameters for MRG-001 (plerixafor and tacrolimus) will be determined, as appropriate:<br>• C_{min}: Minimum plasma MRG-001 concentration determined directly from the concentration-time profile.<br>• C_{max}: Maximum plasma MRG-001 concentration determined directly from the concentration-time profile.<br>• $C_{maxs,s:}$ Maximum MRG-001 concentration at steady state determined directly from the concentration-time profile.<br>• C_{trough}: Concentration of MRG-00 at the end of the dosing interval.<br>• T_{max}: Time of maximum plasma MRG-001 (plerixafor and tacrolimus) concentration determined directly from the concentration-time profile.<br>• AUC_{last}: Area under the concentration-time curve from pre-dose (time 0) to the time of the last quantifiable concentration (t_{last}) calculated using the linear-log trapezoidal rule.<br>• $AUC_{tau,ss}$: Area under the plasma concentration-time curve over a dosing interval at steady state<br>• AUC_{inf}: Area under the c.oncentration-time curve from pre-dose (time 0) extrapolated to infinite time (AUC_{last} + C_{last}/λ_z) calculated using the linear-log trapezoidal rule.<br>• $AUC_{\%extrap}$: Percentage of AUC_{inf} that is due to extrapolation beyond t_{last}

	• λ_z: The terminal elimination rate constant determined by selection of at least three data points on the terminal phase of the concentration-time curve.<br>• $t_{½}$: Terminal elimination half-life calculated as: $\ln 2/\lambda_z$<br>• CL/F: Total body clearance calculated as: Dose/AUC_{inf} .<br>• Vz/F: Apparent volume of distribution calculated as: Dose/(AUC_{inf} * λ_z)<br>• CLr: Renal clearance calculated as: Ae/AUC.<br><br>[redacted]<br><br>[redacted]<br>[redacted]<br>[redacted]<br>[redacted]<br>[redacted].<br>• Percentage change from baseline in biomarkers of inflammation and [redacted]<br>[redacted]

	▪ [redacted]<br>▪ [redacted]<br>▪ [redacted]<br>▪ [redacted]<br>*Safety Endpoints (Part A and Part B):*<br>• Twelve-lead ECG.<br>• Vital signs (supine blood pressure [BP], pulse rate, oral body temperature and respiratory rate [RR]).<br>• Change in clinical laboratory parameters (hematology, clinical chemistry, coagulation,).<br>• Adverse event assessments.<br>• Concomitant medication assessments.<br>• Physical examination.<br>• Injection site complications
**Statistical Methods**	*Sample Size Considerations*<br>In Part A, formal sample size calculations were not performed. Given the stage of development and the nature of these exploratory investigations, the total number of subjects enrolled in the study was based on feasibility and not on statistical power calculations. These sample sizes were considered adequate to meet the study objectives based on clinical and PK considerations and to allow for assessment of the tolerability of the drug and the PK parameters.<br>[redacted]<br>*Data Presentation/Descriptive Statistics*<br>All demographic, safety, efficacy, PK, and PD data will be listed and summarized in tabular format by descriptive statistics as appropriate. Pharmacokinetics data will also be displayed graphically as appropriate.<br>For the primary endpoint in Part B a log-rank test will be used to test the time to improvement from randomization to day 28 by at least 2 levels on the 8-point ordinal scale between MRG-001 and placebo. A Cox proportional hazards model including treatment as a variable and severity of disease, age gender and presence of risk factors as covariates will be used to calculate the hazard ratio and its 95% confidence interval. Kaplan-Meier survival curves for both treatment arms will be presented and the median time to event as well as 95% confidence intervals presented.<br>A statistical analysis plan will provide details of the statistical methodology used for the analysis of Part A and Part B.

1.2. Schema

Table 1 Part A Schema

Cohort	Dose	
1a (sentinel group)	Single SC dose of 0.005 mL/kg MRG-001 (24 mg plerixafor plus 0.5 mg tacrolimus per mL) (n=1)	Placebo (n=1)
1b (remainder of cohort)	Single SC dose of 0.005 mL/kg MRG-001, (24 mg plerixafor plus 0.5 mg tacrolimus per mL) (n=3)	Placebo (n=1)
2a (sentinel group)	Single SC dose of 0.01 mL/kg MRG-001, (24 mg plerixafor plus 0.5 mg tacrolimus per mL) (n=1)	Placebo (n=1)
2b (remainder of cohort)	Single SC dose of 0.01 mL/kg MRG-001, (24 mg plerixafor plus 0.5 mg tacrolimus per mL) (n=3)	Placebo (n=1)
3a (sentinel group)	Single SC dose of 0.02 mL/kg MRG-001, (24 mg plerixafor plus 0.5 mg tacrolimus per mL) (n=1)	Placebo (n=1)
3b (remainder of cohort)	Single SC dose of 0.02 mL/kg MRG-001, (24 mg plerixafor plus 0.5 mg tacrolimus per mL) (n=3)	Placebo (n=1)

Table 2 Part B Schema

[redacted]	[redacted]	
[redacted]	[redacted]	[redacted]
[redacted]	[redacted]	[redacted]
[redacted]	[redacted]	[redacted]
[redacted]	[redacted]	[redacted]

1.2.1. Schedule of Assessments, Time Points and Window Allowance

Details on procedures and timing of assessments for Part A are presented in Table 3 and for Part B in Table 4.

Table 3 Schedule of Assessments (Part A)

Evaluation	Screening	Days											EOS
	-14 to -1	1	2	3	4	5	6	7	8	9	10	11	12
Informed consent	X												
Medical history	X												
Demographics	X												
Inclusion/exclusion criteria	X	X											
Drugs, Alcohol & Cotinine test (Urine)	X	X		X		X		X					
SARS-CoV-2 test (w/in 96 hours)	X												
Assessment of clinical status and disease progression		X	X	X	X	X	X	X					X
Physical examination	X		X		X			X					
Body weight, height, BMI	X	X (weight only)											X (weight only)
Symptom-directed physical examination	X	X	X	X	X	X	X	X					X
Serum pregnancy test (females of CBP)[a]	X	X											
Urine pregnancy test (females of CBP)				X		X		X					X
Viral serology (HBsAG, anti-HCV, anti HIV)	X												
Tuberculosis Gold Test	X												
Prior/concomitant medications	X												
**Safety and tolerability assessments**													
Adverse event monitoring	◄					X							►
Injection site reaction		X	X	X	X	X	X	X					X
Chest X-ray	X												
12-lead ECG	X	X						X					
Troponin[b]	X	X						X					X
Vital signs[c]	X	Xh	X	Xh	X	Xh	X	X					X

Evaluation	Screening	Days											EOS
	-14 to -1	1	2	3	4	5	6	7	8	9	10	11	12
Clinical laboratory tests (clinical chemistry)[d]	X	X (prior to injection)	X (24 hours post injection	X (prior to injection)		X (prior to injection)		X (48 hours after first injection on Day 5)					X

Table 3 Schedule of Assessments (Part A) (Continued)

**Evaluation**	**Screening**	**Days**											**EOS**
	-14 to -1	1	2	3	4	5	6	7	8	9	10	11	12
**Pharmacokinetics**													
Clinical laboratory tests (hematology)[d]	X	X (prior to injection, 1, 3, 8 , 12 h)	X 24 h post Day 1 dose	X 48 h post Day 1 dose and 12 h post Day 3 dose	X 24 h post Day 3 dose	X 48 h post Day 3 dose and 1, 3, 8, 12 h post Day 5 dose	X 24 h post Day 5 dose	X 48 h post Day 5 dose					X
**IMP administration (MRG-001/placebo)**													
Randomization		X											
Dose administration		X		X		X							
PK blood sampling[e]		X prior to injection, 1, 3, 8, 12 h post first dose	X 24 h post Day 1 dose	X 48 h post Day 1 dose and 12 h post Day 3 dose	X 24 h post Day 3 dose	X 48 h post Day 3 dose and 1, 3, 8, 12 h post Day 5 dose	X 24 h post Day 5 dose	X 48 h post Day 5 dose					
**Pharmacodynamics**													
Stems cells[f]		X prior to injection, 1, 3, 8, 12 h post first dose	X 24 h post Day 1 dose	X 48 h post Day 1 dose and 12 h post Day 3 dose	X 24 h post Day 3 dose	X 48 h post Day 3 dose and 1, 3, 8, 12 h post Day 5 dose	X 24 h post Day 5 dose	X 48 h post Day 5 dose					
Immune cells[g]		X prior to injection, 1, 3, 8, 12 h post first dose	X 24 h post Day 1 dose	X 48 h post Day 1 dose and 12 h post Day 3 dose	X 24 h post Day 3 dose	X 48 h post Day 3 dose and 1, 3, 8, 12 h post Day 5 dose	X 24 h post Day 5 dose	X 48 h post Day 5 dose					

AE = adverse event; ALP = alkaline phosphatase; ALT = alanine transaminase; AST = aspartate transaminase; BMI = body mass index; BUN = blood urea nitrogen; CRP = cross reactive protein; ECG = electrocardiogram; EOS = end of study; γGT, = gamma-glutamyltransferase; h = hour; HBsAG = hepatitis B surface antigen;

HCV = hepatitis C virus; HIV = human immunodeficiency virus; IMP = investigational medicinal product; INR = international normalized ratio; LDH = lactate dehydrogenase; PK = pharmacokinetics; TB = tuberculin test.

a. A serum pregnancy test will be conducted at the Screening and Day 1 Visit. Urine pregnancy test will be conducted at all other timepoints. Additional urine pregnancy tests may be performed at the Investigator's discretion.
b. Cardiac injury markers: Troponin I and Troponin T.
c. Xh: Vital signs will be assessed twice on a drug injection day, prior to injection and 4 hours after injection.
d. Clinical laboratory safety evaluations include: Hematology (white blood cell count and differential, hemoglobin, hematocrit, reticulocytes, and platelets) and blood chemistry (ALT, AST, total bilirubin, γGT, ALP, albumin, total protein, LDH, INR, creatinine kinase, sodium, potassium, CO_2, chloride, creatinine, CRP, BUN, Troponin I, Troponin T, and glucose). Coagulation labs PT, APTT are only collected at pre-screening, pre-dose day 1, day 7 and 12.
e. Blood sampling for PK analysis of Tacrolimus and Plerixafor.
f. Stem cell assessments include: CD3, CD19/20, CD33, CD14, CD56, CD16) / CD45 /CD45RA/CD38/CD90/ CD133 / CD31 / CD34 / VEGFR2 / SSEA3 / Live-dead.
g. Immune cell assessments include: CD3 / CD4 / CD8 / CD19 / CD56 / CD16 / CD45RA / CCR7 / CD25 / CD127 /FoxP3 / ICOS / CTLA4 / Live-dead.
h. pre-dose and 4 hours post dose

Table 4 Schedule of Assessments (Part B)

[redacted]	[redacted]							[redacted]								[redacted]
		[redacted]	[redacted]	[redacted]	[redacted]	[redacted]	[redacted]	[redacted]	[redacted]	[redacted]	[redacted]	[redacted]	[redacted]	[redacted]	[redacted]	[redacted]
[redacted]		[redacted]														
															[redacted]	[redacted]
	[redacted]														[redacted]	[redacted]
	[redacted]	[redacted]	[redacted]	[redacted]	[redacted]	[redacted]	[redacted]	[redacted]	[redacted]	[redacted]	[redacted]	[redacted]			[redacted]	[redacted]
	[redacted]	[redacted]			[redacted]										[redacted]	
		[redacted]														[redacted]
[redacted]	[redacted]	[redacted]	[redacted]	[redacted]	[redacted]	[redacted]	[redacted]	[redacted]	[redacted]	[redacted]	[redacted]	[redacted]			[redacted]	[redacted]
	[redacted]	[redacted]														
	[redacted]															
		[redacted]	[redacted]	[redacted]	[redacted]	[redacted]	[redacted]	[redacted]	[redacted]	[redacted]	[redacted]	[redacted]			[redacted]	[redacted]
	[redacted]			[redacted]		[redacted]		[redacted]		[redacted]						

[redacted]	[redacted]	[redacted]	[redacted]	[redacted]		[redacted]				[redacted]	[redacted]	[redacted]			[redacted]	[redacted]

Table 4 Schedule of Assessments (Part B) (Continued)

[redacted]	[redacted]							[redacted]								[redacted]
	[redacted]	[redacted]	[redacted]	[redacted]	[redacted]	[redacted]	[redacted]	[redacted]	[redacted]	[redacted]	[redacted]	[redacted]	[redacted]	[redacted]	[redacted]	[redacted]
[redacted]	[redacted]	[redacted]		[redacted]		[redacted]		[redacted]		[redacted]		[redacted]			[redacted]	[redacted]
[redacted]	[redacted]	[redacted]										[redacted]				
[redacted]	[redacted]														[redacted]	
[redacted]	[redacted]	[redacted]						[redacted]				[redacted]				
[redacted]	[redacted]	[redacted]						[redacted]				[redacted]				
[redacted]	[redacted]	[redacted]						[redacted]				[redacted]				
[redacted]	[redacted]	[redacted]	[redacted]	[redacted]	[redacted]	[redacted]	[redacted]	[redacted]	[redacted]	[redacted]	[redacted]	[redacted]			[redacted]	[redacted]
[redacted]		[redacted]	[redacted]	[redacted]	[redacted]	[redacted]	[redacted]	[redacted]	[redacted]	[redacted]	[redacted]	[redacted]			[redacted]	[redacted]
[redacted]								[redacted]							[redacted]	[redacted]
[redacted]	[redacted]		[redacted]	[redacted]	[redacted]	[redacted]	[redacted]	[redacted]	[redacted]	[redacted]	[redacted]	[redacted]			[redacted]	[redacted]
[redacted]			[redacted]	[redacted]	[redacted]	[redacted]	[redacted]	[redacted]	[redacted]	[redacted]	[redacted]	[redacted]			[redacted]	[redacted]
[redacted]																
[redacted]		[redacted]														
[redacted]		[redacted]		[redacted]		[redacted]		[redacted]		[redacted]						
[redacted]																
		[redacted]	[redacted]	[redacted]		[redacted]		[redacted]		[redacted]	[redacted]	[redacted]				
[redacted]																
[redacted]		[redacted]	[redacted]	[redacted]		[redacted]				[redacted]	[redacted]	[redacted]				

Table 4 Schedule of Assessments (Part B) (Continued)

[redacted]	[redacted]	[redacted]														[redacted]
		[redacted]	[redacted]	[redacted]		[redacted]				[redacted]	[redacted]	[redacted]				

[redacted]

2. INTRODUCTION

MRG-001 (plerixafor or AMD3100, 24 mg/mL) and (tacrolimus or FK506, 0.5 mg/mL) is under development by Medregen, LLC, for the disease caused by severe acute respiratory syndrome-coronavirus-2 (SARS-CoV-2).

There is no previous human experience with MRG-001 as this fixed-dose combination product. This study will be conducted in compliance with the protocol, Good Clinical Practice (GCP), and all applicable regulatory requirements.

2.1. Background

[redacted]

[redacted]

[redacted]

2.1. Pathophysiology of severe acute respiratory syndrome-coronavirus-2 infection

The pathophysiology of the disease is multifactorial and is associated with innate immune response, a hypercoagulability state, lung tissue damage, neurological and/or gastrointestinal tract involvement, and a monocytic/macrophage activation syndrome.

2.1.1. Lymphopenia and cytokine storm

[redacted]

[redacted]

[redacted]

[redacted]

[redacted] [redacted]

[redacted]

[redacted]

[redacted]

[redacted]

[redacted]

[redacted]

[redacted]

[redacted]

[redacted]

[redacted]

[redacted] [redacted]

[redacted]

[redacted] [redacted]

[redacted]

[redacted]

[redacted]

[redacted]

[redacted]

[redacted]

[redacted]

[redacted]

[redacted]

[redacted]

[redacted] [redacted]

[redacted]

[redacted]

[redacted]

[redacted]

[redacted]

[redacted] [redacted]

[redacted] [redacted]

[redacted]

[redacted] [redacted]

[redacted]

[redacted]

[redacted]

[redacted] [redacted]

[redacted]

[redacted]

[redacted] [redacted]

[redacted]

2.4. Summary of Findings from Previous Clinical Studies

No clinical studies have been performed to date with the MRG-001. This is a first-in-human (FIH) clinical study with the study drug.

2.5. Rationale for the Clinical Study

MRG-001 is likely to target multiple aspects of the COVID-19. MRG-001 exhibits immunoregulatory and regenerative properties in animals with a wide variety of diseases. Repairing damaged tissues in the lung and other organs, restoring the anti-virus immune system and modulating the inflammation are obvious therapeutic targets for COVID-19. Thus, MRG-001 may be an ideal candidate drug for treatment of COVID-19. Promoting tissue regeneration by recruiting bone marrow stem cells, modulating the inflammation through activation of regulatory immune cells, restoring antiviral CD8+ T cells and NK cells by mobilizing immune cells (lymphocytes) stored in the bone marrow, MRG-001 has promising great therapeutic potential to improve survival, promote recovery and prevent pulmonary fibrosis in COVID-19 patients, allowing their immune system to clear the virus.

[redacted] [redacted]

[redacted]

[redacted]

[redacted]

[redacted]

[redacted]

[redacted]

[redacted]

- [redacted]
- [redacted]
- [redacted]

- [redacted]
- [redacted]

[redacted]

3. STUDY OBJECTIVES AND ENDPOINTS

3.1. Part A

3.1.1. Primary Objective

- To determine the safety and tolerability, PK and PD (Phase I) of MRG-001 in healthy subjects.

[redacted]

3.3. Endpoints

3.3.1. Part A

3.3.1.1. Primary Endpoint

- Proportion of subjects experiencing any treatment emergent adverse events (TEAEs) associated with MRG-001 from baseline to Day 12.

3.3.1.2. Secondary Endpoints

- Change from baseline in percentages in circulating stem cell and lymphocyte subpopulations concentrations. Stem cell panel: CD3, CD19/20, CD33, CD14, CD56, CD16) / CD45 /CD45RA/CD38/CD90/ CD133 / CD31 / CD34 / VEGFR2 / SSEA3 / Live-dead. Immune cell panel: CD3 / CD4 / CD8 / CD19 / CD56 / CD16 / CD45RA / CCR7 / CD25 / CD127 / FOXP3 / ICOS / CTLA4 / Live-dead to Day 7.
- Change from baseline in white blood cell count, hemoglobin, platelet count to Day 12.
- Change baseline in ALT, AST, total bilirubin, lactate dehydrogenase (LDH), creatinine kinase (CK), creatinine, blood urea nitrogen (BUN), estimated glomerular filtration rate (eGFR) to Day 12.

3.3.1.3. Pharmacokinetic Endpoints

The following PK parameters for MRG-001 (Plerixafor and Tacrolimus) will be determined, as appropriate:

- C_{min}: Minimum plasma MRG-001 concentration determined directly from the concentration-time profile
- C_{max}: Maximum plasma MRG-001 concentration determined directly from the concentration-time profile
- $C_{maxs,s:}$ Maximum MRG-001 concentration at steady state determined directly from the concentration-time profile
- C_{trough}: Concentration of MRG-001 at the end of the dosing interval.
- T_{max}: Time of maximum plasma MRG-001 concentration determined directly from the concentration-time profile
- AUC_{last}: Area under the concentration-time curve from pre-dose (time 0) to the time of the last quantifiable concentration (t_{last}) calculated using the linear-log trapezoidal rule
- AUC_{tau}: Area under the plasma concentration-time curve over a dosing interval at steady state.
- AUC_{inf}: Area under the concentration-time curve from pre-dose (time 0) extrapolated to infinite time ($AUC_{last} + C_{last}/\lambda_z$) calculated using the linear-log trapezoidal rule
- $AUC_{\%extrap}$: Percentage of AUC_{inf} that is due to extrapolation beyond t_{last}
- λ_z: The terminal elimination rate constant determined by selection of at least three data points on the terminal phase of the concentration-time curve.
- $t_{½}$: Terminal elimination half-life calculated as: $\ln2/\lambda_z$
- CL/F: Total body clearance calculated as: Dose/AUC_{inf}
- Vz/F: Apparent volume of distribution calculated as: Dose/(AUC_{inf} * λ_z)
- CLr: Renal clearance calculated as: Ae/AUC

[redacted]

[redacted]

[redacted]

[redacted] [redacted]

- [redacted]
- [redacted]
    - [redacted]
    - [redacted]
- [redacted]
- [redacted]
- [redacted]
- [redacted]
- [redacted]

[redacted] [redacted]

- [redacted]
    - [redacted]
    - [redacted]
    - [redacted]
    - [redacted]
        - [redacted]
        - [redacted]
- [redacted]
- [redacted]

- [redacted]
- [redacted]

3.3.2.4. Safety Endpoints (Parts A and B)

- Twelve-lead ECG.
- Vital signs (supine blood pressure [BP], pulse rate, oral body temperature and respiratory rate [RR]).
- Change in clinical laboratory parameters (hematology [white blood cell count, hemoglobin, platelet count], clinical chemistry [ALT, AST, TBili, LDH, creatinine, creatinine kinase, BUN, eGFR], coagulation [D-dimer- only part B]).
- Adverse event assessments.
- Concomitant medication assessments.
- Physical examinations including site injection assessment

4. STUDY DESIGN

4.1. Overview

This study is a FIH, randomized, double blind, placebo-controlled, 2-part study designed to assess the safety, efficacy, tolerability, PK, and PD of MRG-001 administered SC, QAD for 5 days in 18 healthy subjects, [redacted] see Table 5.

The overall study design is commonly used for Phase 1/2 safety and PK studies. Incorporation of sentinel subjects in any cohort, especially the first cohort, is designed to limit the number of subjects exposed to a potentially unsafe drug or unsafe dose.

Table 5 Study Design

Study Part	Study Population	Design	Planned Number of Cohorts	Subjects Per Cohort	Total # of Subjects
Part A	Healthy adult subjects	Randomized placebo controlled	3	6 (4 active and 2 placebo)	18
[redacted]	[redacted]	[redacted]	[redacted]	[redacted]	[redacted]

Part A will be conducted in a single center and Part B will be conducted in multiple centers in the US.

Part A

Part A will consist of a Screening Visit (Day -14 to -1), a Treatment Period (Day 1 to 5), follow-up visits (Days 6 and 7), and End-of-Study (EOS) Visit at Day 12.

Three dose levels were planned for subjects in Part A: 0,005 mL/kg, 0.01 mL/kg and 0.02 mL/kg. A total of 18 subjects will be enrolled in 3 sequential cohorts (Cohort 1, Cohort 2 and Cohort 3) of 6 healthy volunteer subjects (see Table 1). Subjects will visit the clinical unit on Day 1 to receive the study drug (MRG-001 or placebo) subcutaneously (SC). Subjects will remain at the clinical unit for 24 hours after receiving the study drug so that clinical assessments can be carried out. Subjects will return to the clinical unit to repeat the same procedures QAD for 5 days. The initial dose level will be 0.005 mL/kg based on nonclinical study results. The first cohort will include a sentinel group (Cohort 1a: first 2 subjects with 1 per treatment) dosed at the same time. Dose administration for the remainder of the first cohort (Cohort 1b) will occur only at least 24 hours after the 2 sentinel subjects received study drug (MRG-001 or placebo) and will be contingent upon acceptable safety results through 24 hours for the sentinel subjects per Investigator in consultation with the Sponsor and with explicit agreement from the Sponsor.

Progression to to Cohort 2 and Cohort 3 will be dependent upon safety data through Day 5 from the previous cohort.

The second cohort will receive 0.01 mL/kg MRG-001 or placebo. The cohort will include a sentinel group (Cohort 2a: first 2 subjects with 1 per treatment) dosed at the same time. Dose administration for the remainder of the second cohort (Cohort 2b) will occur only at least 24 hours after the 2 sentinel subjects received study drug (MRG-001 or placebo) and will be contingent upon acceptable safety results through 24 hours for the sentinel subjects per Investigator in consultation with the Sponsor and with explicit agreement from the Sponsor.

Subjects will receive additional doses of MRG-001 or placebo on Days 3 and 5. Follow-up assessments for safety and PK will be scheduled on Days 6 and 7 and an end of study safety assessment on Day 12.

The third cohort will receive 0.02 mL/kg MRG-001 or placebo. The cohort will include a sentinel group (Cohort 3a: 2 subjects; 1 subject receiving MRG-001 and 1 subject receiving placebo) who will be dosed at the same time. Dose administration for the remainder of subjects in the third cohort (Cohort 3b) will occur at least 24 hours after the 2 sentinel subjects received study drug (MRG-001 or placebo) and will be contingent upon acceptable safety results through 24 hours for the sentinel subjects per Investigator in consultation with the Sponsor and with explicit agreement from the Sponsor.

Subjects will receive additional doses of MRG-001 or placebo on Days 3 and 5. Follow-up assessments for safety and PK will be scheduled on Days 6 and 7 and an end of study safety assessment on Day 12.

The Data Safety Review Committee (DSRC) will review all available safety, tolerability, and if feasible and available PK and PD data prior to making a decision to escalate to the next cohort.

The estimated duration of study participation (Screening through EOS Visit [Day 12]) for an individual subject in Part A will be approximately 4 weeks.

[redacted]

[redacted]

[redacted].

[redacted]

[redacted]

[redacted]

[redacted]

[redacted]

The Schedules of Assessments for Parts A and B are provided in Table 3 and Table 4.

Figure 1 Study Flow Chart (Part A)

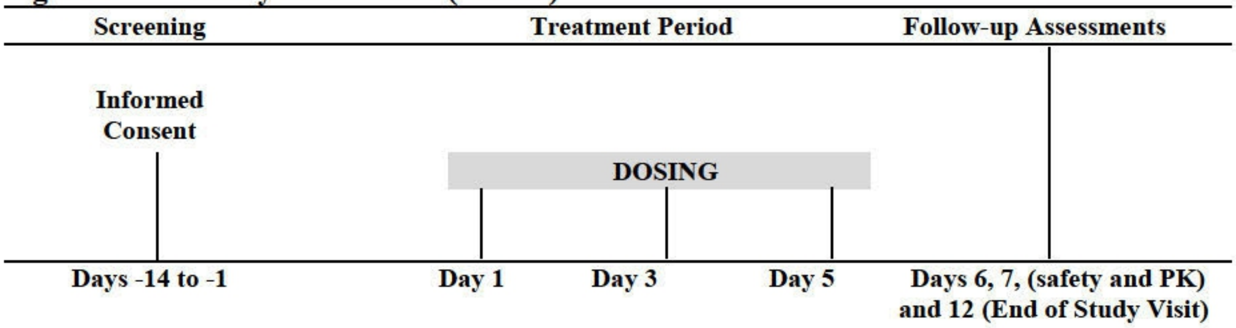

[redacted]

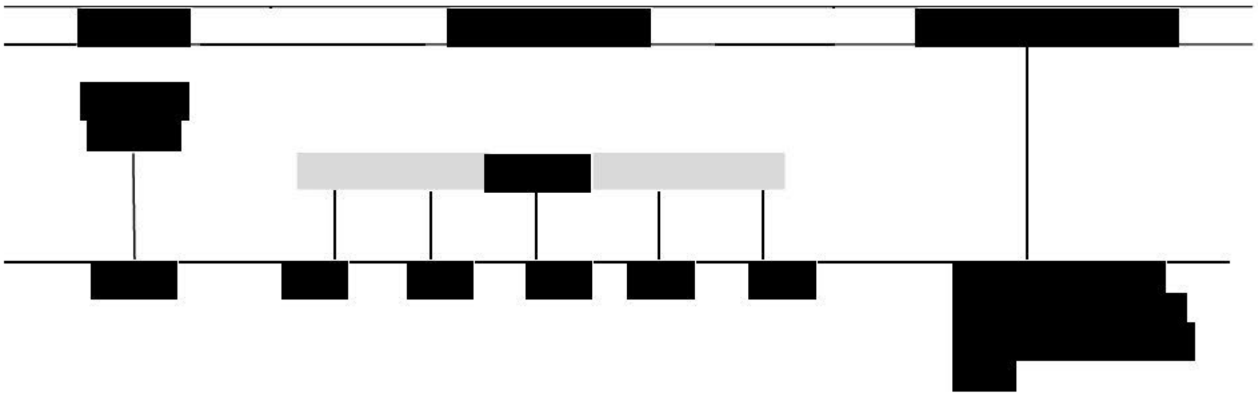

4.2. Scientific Rationale for the Study Design

[redacted]

This study evaluates the safety, tolerability, PK, and PD of MRG-001 administered SC, QAD for 5 days in part A [redacted]. The design is considered appropriate to meet the objectives of the study.

[redacted]

The Principal Investigator, the Sponsor and the Medical Monitor will review available blinded safety, tolerability and if available PK data after each cohort and progression to and the dose level for the next cohort (Cohort 2) will be dependent upon safety data through Day 5 from the previous cohort.

The safety assessments for the study are accepted measures for ensuring safety of subjects during a clinical trial. The PK sampling schedule is considered appropriate given the information available. The rationale for dose selection is discussed in Section 4.3.

4.3. Justification for Dose

[redacted]

[redacted]

[redacted]

[redacted]

[redacted]

[redacted].

[redacted]

[redacted] [redacted]

	[redacted]			[redacted]		
	[redacted]	[redacted]	[redacted]	[redacted]	[redacted]	[redacted]
[redacted]	[redacted]	[redacted]	[redacted]	[redacted]	[redacted]	[redacted]

[redacted]

[redacted]

4.4. Order of Assessments

For both part of the study, the following priority order will be in effect when more than one assessment is required at a pre-dose and post-dose time point, with PK blood sampling being performed nearest to the specified time:

1. 12-lead ECG.
2. Vital signs (supine BP, pulse rate, oral body temperature, RR).
3. PK and PD blood sampling.
4. Blood sampling for safety assessments (hematology, clinical chemistry, coagulation).

4.5. Study Duration

Part A

The duration of participation for each subject will be approximately 3 months. The estimated study duration includes:

1. The screening period: Up to 14 days (Day -14 to -1).
2. Treatment period: Up to Day 5 visit.
3. Follow-up Visits: Days 6 and 7.
4. EOS visit: Day 12.

[redacted]

[redacted]

[redacted] [redacted]

[redacted]

4.6. Study Completion

For the entire study, study completion is defined as the last visit of the last subject for any protocol related activity (last subject, last visit). For individual subjects, study completion is defined as the time of the subject's last data collection.

4.7. Early Termination

For an individual subject, study completion is defined as completing the Treatment Period and the EOS Visit.

If a subject withdraws prematurely after dosing, subjects will undergo the procedures for the EOS Visit described in Table 3 and Table 4.

The reason for discontinuation must be recorded in the case report form (CRF).

5. STUDY POPULATION

[redacted]

Subjects participating in Part A must be able to provide written informed consent before commencing the study. Subjects in Part B must be able to provide written informed consent or have a legal representative who can provide informed consent or who can be enrolled under International Conference on Harmonization (ICH) E6 (R2) 4.8.15 emergency use provision. Subjects must meet all the inclusion criteria and none of the exclusion criteria.

5.1. Number of Subjects

[redacted]

Part A

Part A will consist of 3 dose groups of healthy subjects with 6 subjects per dose group (18 subjects). Within each dose group, 4 subjects will be randomized to receive a single SC injection of MRG-001 (0.005 mL/kg, 0.01 mL/kg or 0.02 mL/kg and 2 subjects will be randomized to receive a single SC injection of matching placebo.

[redacted]

[redacted]

5.2. Inclusion Criteria

Part A

Subjects who meet the following criteria will be considered eligible to participate in the clinical study:

1. Subject voluntarily agrees to participate in this study and signs an Institutional Review Board (IRB)-approved informed consent prior to performing any of the Screening Visit procedures.
2. Males and females between 18 to 45 years of age, inclusive, at the time of signing the ICF.
3. Subjects who test negative for SARS-CoV-2 by real time transcription polymerase chain reaction in the respiratory tract (nasopharyngeal [NP] swab) within the previous 96 hours.
4. Nonsmokers (or other nicotine use) as determined by history (no nicotine use over the past 6 months) and by urine cotinine concentration (< 200 ng/mL) at the Screening Visit and prior to admission.
5. Generally, in good health with no clinically significant abnormalities as determined by medical history, physical examination, 12-lead ECG and clinical laboratory tests.
6. The following applies to female subjects:
   - Non-pregnant, non-lactating females of childbearing potential who agree to use medically acceptable forms of birth control (hormonal contraception, abstinence, diaphragm with spermicide, condom with spermicide or intrauterine device) from the Screening Visit until the End-of-study Visit.
7. Body mass index (BMI) between 18.8 and 32.0 kg/m^2, inclusive, at the Screening Visit.
8. A fasting blood glucose level ≤125 mg/dL (6.9 mmol/L), at the Screening Visit.

[redacted]

[redacted]

[redacted] [redacted]

[redacted]

[redacted] [redacted]

[redacted] [redacted]

[redacted] [redacted]

[redacted] [redacted]

[redacted] [redacted]

[redacted] [redacted]

[redacted] [redacted]

[redacted] [redacted]

7. A fasting blood glucose level ≤140 mg/dL (7.8 mmol/L), at the Screening Visit.

5.3. Exclusion Criteria

Subjects who meet one or more of the following criteria will not be considered eligible to participate in the clinical study:

Part A and Part B Subjects:

1. Participation in any other clinical trial of an experimental treatment for COVID-19 (remdesivir and convalescent plasma use is permitted).

2. Subject has clinically significant history or evidence of cardiovascular, respiratory, hepatic, renal, gastrointestinal, endocrine, neurological, immunological or psychiatric disorder(s) as determined by the Principal Investigator or designee.
3. Concurrent treatment with other agents with actual or possible direct acting immunomodulatory activity against ARDS in COVID-19 is prohibited <72 hours prior to study drug dosing [IL-6 inhibitors such as sarilumab and tocilizumab; IL-1β blocker; and the JAK1/JAK2 inhibitor ruxolitinib, barcitinib and tofacitinib; complement inhibitor ravulizumab-cwvz; Bruton's tyrosine kinase inhibitor acalabrutinib, and macrophage migration inhibitor ibudilast].
4. History of splenomegaly (spleen weighing >750 g).
5. History of cancer or thrombocytopenia (platelet count <100,000/µL) or thrombocythemia (platelet count >500,000/µL).
6. Known family history of long QT syndrome (Torsades de Pointes) or currently taking medication that prolongs QT interval.
7. Currently taking immunomodulating biologics (e.g, interferons, interleukin).
8. Female subjects who are pregnant or breastfeeding or planning to breastfeed at any time through 90 days after last dose of IP.
9. Any disorder that would interfere with the absorption, distribution, metabolism or excretion of drugs.
10. Received a vaccination (including influenza) administered 30 days or less prior to first treatment/randomization or has any planned vaccinations during the treatment period.
11. Creatinine clearance <50 mL/min using the Cockcroft-Gault formula.
12. Has the following liver function levels:

    Serum ALP or BIL >1.5 ULN or ALT or AST >ULN (Part A);

    Serum ALP or BIL >3.0 ULN or ALT or AST >5.0x ULN (Part B);

    at either screening or admission. Only 1 repeat assessment is allowed on each occasion.
13. History of alcohol and/or illicit drug abuse within 2 years of entry.
14. Positive test for Hepatitis B surface antigen (HBsAg), Hepatitis C antibody or human immunodeficiency virus (HIV) antibody.

15. Has a positive urine test for ethanol at the Screening Visit or admission.
16. Has a positive urine drug test (e.g., cocaine, amphetamines, barbiturates, opiates, benzodiazepines, cannabinoids) at the Screening Visit or admission.
17. Has donated blood (>500 mL) or blood products within 2 months (56 days) prior to admission.
18. Has used an investigational drug within 30 days prior to Screening.
19. History of hypersensitivity to MRG-001 (plerixafor [AMD3100, 24 mg/mL]) and tacrolimus [FK506, 0.5 mg/mL]) or any of the excipients or to medicinal products with similar chemical structures.
20. Unable to understand the protocol requirements, instructions and study related restrictions, the nature, scope and possible consequences of the clinical study.
21. Unlikely to comply with the protocol requirements, instructions and study related restrictions; e.g., uncooperative attitude, inability to return for follow-up visits and improbability of completing the clinical study.
22. Previously been enrolled in this clinical study.
23. Vulnerable subjects defined as individuals whose willingness to volunteer in a clinical study may be unduly influenced by the expectation, whether justified or not, of benefits associated with participation, or of a retaliatory response from senior members of a hierarchy in case of refusal to participate (e.g., persons in detention, minors and those incapable of giving consent).

Part A Subjects Only:

1. Laboratory-confirmation of positive SARS-CoV-2 by real time polymerase chain reaction in the respiratory tract (NP swab, tracheal aspirate, BAL) ≤96 hours prior to randomization.
2. Is unwilling to avoid use of alcohol or alcohol-containing foods, medications or beverages, within 48 hours prior to screening and for the duration of the study.
3. Is unable to abstain from smoking (or other nicotine use) from screening and for the duration of the study.
4. Has any concurrent disease or condition that, in the opinion of the Principal Investigator, would make the subject unsuitable for participation in the clinical study such as (Part A only):
    a. Skin condition or disease (e.g., Stevens-Johnson syndrome).

b. Hypertension defined as >140 mmHg systolic blood pressure and >95 mmHg diastolic blood pressure.

c. High blood potassium (hyperkalemia) defined baseline serum potassium >5.0 to 5.5 mEq/L (milliequivalent).

d. Torsades de Pointes or currently taking medication that prolongs QT interval.

e. Hematologic disorder (e.g. anemia or leukemia).

f. Type I or Type 2 diabetes mellitus defined as a fasting blood glucose level >126 mg/dL (7.0 mmol/L).

[redacted]

[redacted] [redacted]

[redacted] [redacted]

[redacted] [redacted]

5.4. Restrictions

5.4.1. Activity

Vasovagal reactions, orthostatic hypotension, and/or syncope can occur following SC injections of plerixafor. The majority of these events are known to occur within 1 hour of plerixafor administration. Because of the potential for these reactions, Part A participants were required to lie down with limited movement (e.g., using the restroom, sitting up to eat, movements required for the protocol required assessments) and were to be dosed in the supine position.

5.4.2. Dietary and Fluid Restrictions

Part A only

Fasting

Part A subjects, will be required to fast 1 hour pre-dose and 1 hour post-dose. On non-dosing days subjects are required to fast 8 hours prior to scheduled chemistry blood collection as outlined in the schedule of assessments (Part A).

Alcohol

For Part A subjects, consumption of alcohol and alcohol-containing foods, medications or beverages must be avoided from 48 hours before any study visit and for the entire duration of the study.

5.4.3. Lifestyle Considerations

Part A only

Drugs of abuse

Subjects must refrain from use of recreational drugs for the duration of the study.

Nicotine

No smoking or use of other nicotine-containing products (snuff, chewing tobacco, cigars, pipes or nicotine-replacement products such as nicotine chewing gum and nicotine plasters) will be allowed during the study.

5.4.4. Medication

Medication restrictions applicable before dosing are described in Section 5.3 (exclusion criteria). Prior medication will be recorded.

Any medicinal product, prescribed or OTC, taken by a subject other than the IMP, is considered concomitant medication. Use of concomitant medication will be recorded and reported.

5.4.5. Contraception Rules

Contraception requirements for male and female subjects are described below.

Definitions

Woman of Childbearing Potential

A woman is considered fertile following menarche and until becoming post-menopausal unless permanently sterile (see below).

Women in the following categories are not considered to be woman of childbearing potential:

1. Premenarchal

2. Premenopausal female with one of the following:
   - Documented hysterectomy.
   - Documented bilateral salpingectomy.
   - Documented bilateral oophorectomy.

Note: Documentation can come from the site personnel's review of the subject's medical records, medical examination, or medical history interview.

3. Post-menopausal female
   - A post-menopausal state is defined as no menses for 12 months without an alternative medical cause. A high FSH level in the post-menopausal range may be used to confirm a post-menopausal state in women not using hormonal contraception or hormonal replacement therapy (HRT). However, in the absence of 12 months of amenorrhea, a single FSH measurement is insufficient.
   - Females on HRT whose menopausal status is in doubt will be required to use one of the non-estrogen highly effective contraception methods if they wish to continue their HRT during the study. Otherwise, they must discontinue HRT to allow confirmation of post-menopausal status before study enrollment.

Contraception Guidance:

Female Subjects:

Female subjects are allowed to participate in the study if they are:

a) **Not of childbearing potential** _or_

b) Non-pregnant, non-lactating with negative pregnancy test at all visits and who use at least one of the following highly effective contraception options:

   i. Stable hormonal contraceptive for ≥ 90 days prior to screening and for at least 7 days after final dose. If < 90 days prior to the study, additional use of one other effective contraception method until 90 days are reached is required, _or_

   ii. Placement of an intrauterine device or intrauterine hormone-releasing system, _or_

   iii. Use of double barrier methods of contraception (e.g., male condom with diaphragm, male condom with cervical cap), _or_

iv. Successful male sterilization of the sole partner (subject must verbally confirm that appropriate post-vasectomy documentation of the absence of sperm in the ejaculate was provided after the procedure), *or*

v. True abstinence, when in line with the preferred and usual lifestyle of the subject.

Male Subjects:

Male subjects are eligible to participate if they agree to use effective contraception with female partners of childbearing potential from the time of signing the informed consent form (ICF) until at least 8 weeks after their last dose of study drug. Highly effective methods of contraception are:

- Double contraception with barrier and a highly effective hormonal method of contraception (oral, intravaginal or transdermal combined estrogen and progestogen hormonal contraception associated with inhibition of ovulation, oral, injectable or implantable progestogen-only hormonal contraception associated with inhibition of ovulation or intrauterine hormone-releasing system). The hormonal contraception must be started at least one month prior to inclusion.
- Double-barrier birth control (e.g., male condom, female condom, diaphragm sponge, or cervical cap together with spermicidal foam/gel/film/suppository) starting at the Screening visit, throughout the study, and for 8 weeks after the last dose of study drug.
- Documented bilateral vasectomy

Note: Documentation can come from the site personnel's review of the subject's medical records, medical examination, or medical history interview.

Note: A female condom and a male condom should not be used together as friction between the 2 can result in either or both products failing.

- Intrauterine contraception/device starting at the Screening visit, throughout the study, and for 8 weeks after the last dose of study drug.
- Total abstinence from sexual intercourse (only acceptable if it is the preferred and usual lifestyle of the subject) for at least 1 complete menstrual cycle prior to the Screening visit, throughout the study, and for 8 weeks after the last dose of study drug.

Note: Periodic abstinence (calendar, symptothermal, postovulation methods), withdrawal (coitus interruptus), spermicides only, and lactational amenorrhea method are not acceptable methods of contraception.

In addition, male subjects must refrain from donating sperm for the duration of the study and for 8 weeks after the last dose of study drug.

Male subjects with a pregnant or breastfeeding partner must agree to remain abstinent from penile-vaginal intercourse or use a male condom during each episode of penile penetration from the time of signing the ICF until at least 8 weeks after their last dose of study drug.

Collection of Pregnancy Information:

Male Subjects with Female Partners Who Become Pregnant

- The Principal Investigator will attempt to collect pregnancy information on any male subject's female partner who becomes pregnant while the male subject is in this study. This applies only to male subjects who receive the study drug.
- After obtaining the necessary signed informed consent from the pregnant female partner directly, the PI will record pregnancy information on the appropriate form and submit it to the Sponsor within 24 hours of learning of the partner's pregnancy. The female partner will also be followed to determine the outcome of the pregnancy. Information on the status of the mother and child will be forwarded to the Sponsor. Generally, the follow-up will not be required after birth of the child or elective termination of pregnancy. Any termination of the pregnancy will be reported regardless of fetal status (presence or absence of anomalies) or indication for the procedure.

6. INVESTIGATIONAL MEDICINAL PRODUCT

6.1. Identity of the Investigational Medicinal Products

The products that will be used in this study are outlined in Table 7.

Table 7 Identity of Investigational Products

Drug Name	Formulation	Strength, Dosage		Route	Manufacturer
MRG-001	Sterile Solution	Strength:<br>Dose:<br>mL/kg of	AMD3100, 24 mg/mL and tacrolimus (FK506, 0.5 mg/mL)<br>0.005 mL/kg, 0.01 mL/kg, 0.02 subject bodyweight	SC	Sponsor
Saline	Sterile Solution	0.9%		SC	Hospital pharmacy

SC = subcutaneous

Data source: Investigator Brochure, Version 1.0.

6.2. Supply, Packaging, Labeling and Storage

[redacted]

[redacted]

[redacted]

[redacted]

[redacted]

[redacted]

[redacted]

6.4. Subject Identification and Randomization

6.4.1. Screening Numbers

All screened subjects are assigned a unique subject identification (SID) number. The SID numbers are random, 4- to 5-digit numbers that identify subjects from time of Screening until time of randomization. Enrolled subjects who drop out of the clinical study before randomization will retain their SID number.

6.4.2. Randomization numbers

Prior to dosing on Day 1, subjects will be assigned a randomization number in accordance with the randomization code generated by ICON. The randomization code will be maintained in a room with restricted access to pharmacy personnel only. The randomization code will include 3-digit subject numbers starting with 101.

Once a randomization number has been allocated to one subject, it may not be assigned to another subject.

If subjects withdraw prematurely from the study and are replaced under the direction of the Sponsor, then a replacement randomization number will be assigned. A replacement randomization code will be generated such that replacement subjects are assigned to the same treatment as the discontinued subjects. The replacement randomization code will differ only in randomization numbers, which will be 4-digit numbers starting with a leading 1. For example, if Subject 202 withdraws and is replaced, then the randomization number for the replacement subject will be 1202.

6.5. Administration of Investigational Medicinal Products

6.5.1. Part A

Subjects will be enrolled in 3 sequential cohorts (Cohort 1, Cohort 2 and Cohort 3: MRG-001 0.005 mL/kg, 0.01 mL/kg or 0.02 mL/kg of subject body weight, respectively) of 6 subjects each. MRG-001 will be administered through the skin of the abdominal wall SC every other day (QAD) for 5 days. Cohort 1a and Cohort 2a will consist of a sentinel group (two subjects: one subject

receiving active treatment and the other subject receiving matching placebo) dosed at least 10 mins apart (see Table 1). Dose administration for the next subjects in each cohort (Cohort 1b and Cohort 2b and Cohort 3b) will occur approximately 24 hours after the two sentinel subjects received study medication. The decision to proceed to dosing these subjects will be made by the PI based on all available safety, tolerability, and if available PK data from the sentinel subjects up to 24 hours post-dose. For high dosed (0.02 mL/kg) subjects with a body weight > 75kg, the dose may be administered over 2 injection sites per dose to reduce volume/irritation at the injection site.

The injections will be performed in a prescribed, standardized fashion to facilitate site injection review for any AE (Figure 3). Injection site reaction including pain, swelling, rash, bleeding, itching or redness will be closely monitored.

Vasovagal reactions, orthostatic hypotension, and/or syncope can occur following SC injections of plerixafor often within 1 hour of administration. Because of the potential for these reactions, appropriate precautions will be taken to minimize any potential adverse reactions, i.e. dosing in supine position.

Figure 3 Standardized Injection Sites for Study Drug Injection

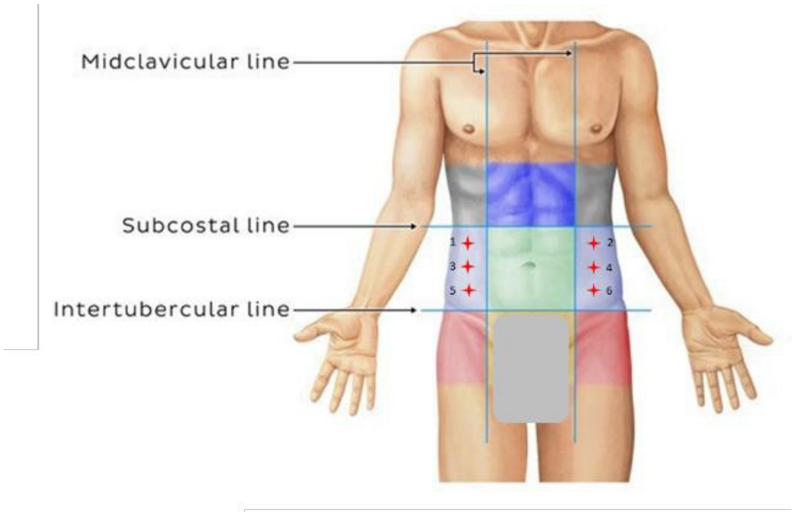

The estimated duration of study participation (Screening through EOS Visit) for an individual subject will be approximately four weeks.

The Schedule of Assessments for Part A is provided in Table 3.

[redacted] [redacted]

[redacted]

[redacted]

6.6. Compliance

Dosing will be performed by trained, qualified personnel designated by the Principal Investigator. No formal assessment of treatment compliance will be required. The date and time of dosing will be documented on each dosing day. The date and time of breakfast will also be documented. Comments will be recorded if there are any deviations from the planned dosing procedures.

6.7. Blinding and Breaking the Blind

The clinical study will be performed in a double-blind manner.

The study blind should not be broken except in a medical emergency (where knowledge of the study drug administered would affect the treatment of the emergency). The decision to break the

blind will be made on a case-by-case basis, at the discretion of the Principal Investigator in collaboration with the Sponsor and/or Medical Monitor. The applicable SOP will be followed for blind breaking procedures.

After database lock, the overall randomization code will be broken only for reporting purposes.

Suspected unexpected serious adverse reactions (SUSARs), that are subject to expedited reporting, should be unblinded by the Sponsor before submission to the regulatory authority and the IRB.

6.8. Treatment of Overdose

Standard symptomatic support measures should be used in the case of excessive pharmacological effects or overdose. No antidotes are available.

7. DISCONTINUATION

7.1. Dose Escalation and Study Stopping Rules

The stopping rules described in this section of the clinical study protocol are applicable to staggered dosing in a cohort, dose escalation to a next cohort and to stopping the study. Dosing may be halted temporarily to investigate before the entire study is terminated.

Measures to ensure data integrity and safety of research subjects will be detailed in the Data and Safety Monitoring Plan (DSMP), which will be signed between the Sponsor and CRO. In summary, these measures include:

- Clearly defined inclusion and exclusion criteria
- Clear individual and study stopping rules
- DSRC for dosing decision-making; dose escalation criteria
- ICH-compliant AE monitoring, reporting and follow-up

If the Principal Investigator, the Medical Monitor or the Sponsor becomes aware of conditions or events that suggest a possible hazard to subjects if the clinical study continues, then the clinical study may be terminated after appropriate consultation among the involved parties. The clinical study may be terminated at the Sponsor's discretion also in the absence of such a finding.

Should the study be terminated, and/or the study centre closed for whatever reason, all documentation pertaining to the study and study drug must be returned to the Sponsor. Any actions of the contract research organisation (CRO) required for assessing or maintaining subject safety will continue as required, despite termination of the study by the Sponsor.

7.1.1. Data Safety Review Committee

A DSRC consists at a minimum of the PI or designee (e.g., a medically qualified sub-investigator, an independent medical monitor, the Sponsor representative, the pharmacokineticist, and the CRO Project Manager who will meet and review the blinded safety, if feasible and available PK and PD data from each cohort in each part of the study on a regular basis. Study progression to the next cohort (within each part of the study) or to the next part of the study will occur only after thorough review of the blinded safety, tolerability, and if feasible and available PK and PD data.

Safety data to be assessed prior to each dose escalation decision within a part of the study include but are not limited to the following:

- AEs including local tolerability assessments
- Concomitant medication assessments.
- Worsening of clinical status.
- Clinical safety laboratory tests (hematology, biochemistry, coagulation).
- Vital signs (supine BP, pulse rate, oral body temperature and respiratory rate).
- Physical examination.
- 12-lead ECGs.

In addition, PK data for MRG-001 will be considered during all parts of the study when available.

At each dose escalation decision in Parts A and B, the DSRC will recommend proceeding or not proceeding to the next dose level.

7.1.2. Stopping Rules

7.1.2.1. Safety Criteria

If any of the following scenarios occur within a cohort with reasonable possibility of a causal relationship with study drug, dosing will be stopped, and further dosing will not be initiated.

For subjects in Part A and Part B:

- One or more subjects report an SAE considered by the Principal Investigator or designee to be at least possibly related to the study drug.
- Two or more subjects in the same cohort with severe non-serious adverse reactions (i.e., severe AEs considered at least possibly related to the IMP administration, independent of within or not within the same system organ class).

For subjects in Part A:

    - One or more subjects experience increased alanine aminotransferase (ALT) or aspartate aminotransferase (AST) > 3 x ULN and total bilirubin (TBL) > 2 x ULN (Hy's Law)
    - Increased ALT > 5 x ULN accompanied by alkaline phosphatase (ALP) > 1.5 x ULN.

- One or more subjects have an increase of more than 60 ms in QTcF compared to the baseline QTcF (baseline QTcF = value obtained on Day 1 at pre-dose in the treatment period) or QTcF > 500 ms confirmed by the average of 2 additional ECG recordings (Repeat ECG number 1 will be performed approximately 10 minutes after the scheduled ECG and Repeat ECG number 2 will be performed approximately 10 minutes after Repeat ECG number 1) or other clinically significant conduction disturbance or arrhythmia. All ECGs for this determination must be performed under strict resting conditions.
- Two or more subjects, who receive IMP, have tachycardia defined as resting supine HR > 125 beats per minute (bpm) persisting for at least 10 minutes.
- Two or more subjects, who receive IMP, have symptomatic bradycardia defined as resting supine HR < 40 bpm while awake persisting for at least 10 minutes.
- Two or more subjects, who receive IMP, develop hypertension defined as an increase in resting supine systolic BP > 40 mmHg to above a reading of 180 mmHg and persisting for at least 10 minutes.
- A subjects develops severe injection site related adverse event without improvement in 24 hours.

[redacted]

- [redacted]
- [redacted]
  - [redacted]
  - [redacted]

- [redacted]
- [redacted]

- [redacted]
  - [redacted]
  - [redacted]
- [redacted]
- [redacted].

[redacted].

7.1.2.2. Pharmacokinetic Criteria

[redacted]

[redacted]

- [redacted]
- [redacted]

[redacted]

- [redacted]
- [redacted]

[redacted]

[redacted]

[redacted]

[redacted]

[redacted]

[redacted]

7.2. Subject Withdrawal and Replacement

In addition to the stopping rules described in Section 7.1.1, a subject will be withdrawn by the Principal Investigator or designee from the study and not be allowed to continue with the study if any of the following criteria are fulfilled:

- Withdrawal of consent by the subject
- Withdrawal of subject by the Principal Investigator or designee if inter-current illnesses occur that may invalidate the study data, if the subject was enrolled in violation of the study protocol, or if a significant study protocol violation occurred, at the discretion of the Principal Investigator or designee
- If discovered that the subject has entered the study in violation of the inclusion/exclusion criteria stated in the protocol
- Critical protocol violation occurs during the study

At the discretion of the Principal Investigator or designee, the subject may continue with study assessments after discontinuation until the final follow-up visit procedures were performed. A reasonable effort will be made to determine the reason(s) why a subject fails to return for the necessary visits or is discontinued from the study. If the subject is unreachable by telephone, a registered letter, at the minimum, should be sent to the subject requesting him/her to contact the study centre. Investigator or the Sponsor stops the study, for any reason (e.g., suspension or discontinuation of study drug development)

Subjects who withdraw or are withdrawn will be replaced, except those withdrawn due to AEs or for safety reasons.

The Principal Investigator or the Sponsor may stop the study, for any reason (e.g., suspension or discontinuation of study drug development).

7.3. Eligibility Screening

Subjects who meet all of the following criteria at the screening and admission visits will be considered eligible to participate in the clinical study:

Part A

- Male and female adults, aged 18 to 45 years of age, inclusive.
- Subjects who test negative for SARS-CoV-2 by real time transcription polymerase chain reaction in the respiratory tract (NP swab) within the previous 96 hours prior to admission.
- Subjects in good health with no clinically significant abnormalities as determined by medical history, physical examination, 12-lead ECG and clinical laboratory tests.
- Females of non-childbearing potential (surgically sterile [hysterectomy or oophorectomy] or postmenopausal (amenorrhea for more than 12 months with FSH in postmenopausal range confirmed by an FSH test).
- Males must be unable to procreate (defined as surgically sterile [i.e., had a vasectomy ≥6 months prior to screening]) or must agree to use a highly effective form of birth control from screening through 8 weeks after study completion.
- Nonsmokers (or other nicotine use) as determined by history (no nicotine use over the past 6 months) and by urine cotinine concentration (< 200 ng/mL).
- Body mass index between 18.8 and 32.0 kg/m^2
- A fasting blood glucose level ≤125 mg/dL (6.9 mmol/L), at the Screening Visit.
- Subject has negative test for HBsAg, Hepatitis C antibody, HIV antibody, or TB test.
- Subject has a negative urine test for alcohol and cotinine.
- Subject has a negative urine drug test (e.g., cocaine, amphetamines, methamphetamines, barbiturates, opiates, benzodiazepines, cannabinoids).
- Subject has not used an investigational drug within 30 days prior to Screening.
- Subject with ALP or BIL <1.5 ULN or ALT or AST > ULN at either screening or admission.

[redacted]

[redacted]

- [redacted]
- [redacted]
- [redacted]
    - [redacted]
    - [redacted]
- [redacted]
- [redacted]
- [redacted]
- [redacted]
- [redacted]
- [redacted]
- [redacted]
- [redacted]
- [redacted]
- [redacted]
- [redacted]

Clinical laboratory tests are listed in Table 9.

7.4. Medical History, Demographic and Other Baseline Information

The medical history comprises:

- General medical history
- Medication history
- Reproductive history

The following demographic information will be recorded:

- Age
- Ethnic origin (Hispanic/Latino or not Hispanic/not Latino)
- Race (White, American Indian/Alaska Native, Asian, Native Hawaiian or other Pacific Islander, Black/African American)
- Height, without shoes (cm)
- Body weight, without shoes (kg)
- Body mass index (BMI) (kg/m^2)

Other baseline characteristics will be recorded as follows:

- History of drug abuse
- History of alcohol abuse
- Smoking history
- History of caffeine use (or other stimulating beverages)
- History of blood or plasma donation

7.5. Safety Variables

7.5.1. Adverse Events

Adverse event reporting will begin for each subject from the date the informed consent form (ICF) is signed and will continue until the End-of-study Visit.

7.5.1.1. Definitions

7.5.1.1.1. Definition of Adverse Event

Any untoward medical occurrence in a patient or clinical investigation subject administered a pharmaceutical product and which does not necessarily have a causal relationship with this treatment. An adverse event (AE) can therefore be any unfavorable and unintended sign (including an abnormal laboratory finding), symptom, or disease temporally associated with the use of a medicinal (investigational) product, whether or not related to the medicinal (investigational) product.

Other untoward events occurring in the framework of a clinical study will be recorded as AEs, e.g. those occurring during treatment-free periods (including Screening or post-treatment follow-up periods), in association with study-related procedures and assessments, or under placebo. For study drugs, lack of efficacy may be an expected potential outcome and should not be reported as an AE unless the event is unusual in some way, e.g., greater in severity.

Concomitant illnesses, which existed prior to entry into the clinical study, will not be considered AEs unless they worsen during the treatment period. Pre-existing conditions will be recorded as part of the subject's medical history.

8.12 Definition of a Treatment-related Adverse Event

A treatment-emergent adverse event or serious adverse event (TEAE or TESAE) is defined as any event not present prior to the initiation of the drug treatment or any event already present that worsens in either intensity or frequency following exposure to the drug treatment.

7.5.1.1.2. Definition of Serious Adverse Event

An SAE is defined as any untoward medical occurrence that at any dose:

- Results in death
- Is life-threatening; this means that the subject was at risk of death at the time of the event; it does not mean that the event hypothetically might have caused death if it were more severe
- Requires inpatient hospitalization or prolongation in existing hospitalization
- Results in persistent or significant disability/incapacity or substantial disruption of the ability to conduct normal life functions
- Is a congenital anomaly/birth defect, or

- Is another important medical event (see below)

Important medical events that do not result in death, are not life-threatening or do not require hospitalization may be considered SAEs when, based on appropriate medical judgment, they may jeopardize the subject and may require medical or surgical intervention to prevent one of the outcomes listed above. Examples of such medical events include allergic bronchospasm requiring intensive treatment in an emergency room or in a physician's office, blood dyscrasias or seizures that do not result in in-patient hospitalization, and the development of drug dependency or drug abuse.

A distinction should be drawn between serious and severe AEs. Severity is a measure of intensity whereas seriousness is defined by the criteria above. For example, a mild degree of gastrointestinal bleeding requiring an overnight hospitalization for monitoring purposes would be considered an SAE but is not necessarily severe. Similarly, an AE that is severe in intensity is not necessarily an SAE. For example, alopecia may be assessed as severe in intensity but would not be considered an SAE.

Medical and scientific judgment should be exercised in deciding if an AE is serious and if expedited reporting is appropriate.

7.5.1.2. Recording of Adverse Events

Adverse events should be collected and recorded for each subject from the date the ICF is signed until the end of their participation in the study, i.e. the subject has discontinued or completed the study.

Adverse events may be volunteered spontaneously by the subject, or discovered by the study staff during physical examinations or by asking an open, non-leading question such as 'How have you been feeling since you were last asked?' All AEs and any required remedial action will be recorded. The nature of AE, date (and time, if known) of AE onset, date (and time, if known) of AE outcome to date, severity and action taken of the AE will be documented together with the Principal Investigator's assessment of the seriousness of the AE and causal relationship to study drug and/or study procedure.

All AEs should be recorded individually in the subject's own words (verbatim) unless, in the opinion of the Principal Investigator, the AEs constitute components of a recognized condition, disease or syndrome. In the latter case, the condition, disease or syndrome should be named rather

than each individual symptom. The AEs will subsequently be coded using the Medical Dictionary for Regulatory Activities (MedDRA).

All AE's and SAE's will be recorded from the time of subject consent until study completion. All AEs/SAE's reported following subject discharge from the study and for 30 days following last dose will be collected and reported.

7.5.1.3. Assessment of Adverse Events

Each AE will be assessed by the Principal Investigator about the categories discussed in the following sections.

7.5.1.3.1. Intensity

The Principal Investigator will assess all AEs for severity in accordance with the following standard ratings.

- Mild: Ordinarily transient symptoms, does not influence performance of subject's daily activities. Treatment is not ordinarily indicated.
- Moderate: Marked symptoms, sufficient to make the subject uncomfortable. Moderate influence on performance of subject's daily activities. Treatment may be necessary.
- Severe: Symptoms cause considerable discomfort. Substantial influence on subject's daily activities. May be unable to continue in the study and treatment may be necessary.

When changes in the intensity of an AE occur more frequently than once a day, the maximum intensity for the event should be noted for that day. Any change in severity of signs and symptoms over a number of days will be captured by recording a new AE, with the amended severity grade, and the date (and time, if known) of the change.

7.5.1.3.2. Causality

The Principal Investigator will assess the causality/relationship between the study drug and the AE. One of the categories described in Table 8 should be selected based on medical judgment, considering the definitions below and all contributing factors.

Table 8 Assessment of Relationship of Adverse Events to Investigational Product

Related	A clinical event, including laboratory test abnormality, occurs in a plausible time relationship to treatment administration, and which concurrent disease or other drugs or chemicals cannot explain. The response to withdrawal of the treatment (dechallenge*) should be clinically plausible. The event must be definitive pharmacologically or phenomenologically, using a satisfactory rechallenge† procedure if necessary.
Probably related	A clinical event, including laboratory test abnormality, with a reasonable time sequence to administration of the treatment, unlikely to be attributed to concurrent disease or other drugs or chemicals, and which follows a clinically reasonable response on withdrawal (dechallenge). Rechallenge information is not required to fulfil this definition.
Possibly related	A clinical event, including laboratory test abnormality, with a reasonable time sequence to administration of the treatment, but which could also be explained by concurrent disease or other drugs or chemicals. Information on treatment withdrawal may be lacking or unclear.
Unlikely to be related	A clinical event, including laboratory test abnormality, with a temporal relationship to treatment administration which makes a causal relationship improbable, and in which other drugs, chemicals or underlying disease provide plausible explanations.
Unrelated	A clinical event, including laboratory test abnormality, with little or no temporal relationship with treatment administration. May have negative dechallenge and rechallenge information. Typically explained by extraneous factors (e.g., concomitant disease, environmental factors or other drugs or chemicals).

*Dechallenge is when a drug suspected of causing an AE is discontinued. If the symptoms of the AE disappear partially or completely, within a reasonable time from drug discontinuation, this is termed a positive dechallenge. If the symptoms continue despite withdrawal of the drug, this is termed a negative dechallenge. Note that there are exceptions when an AE does not disappear upon discontinuation of the drug, yet drug-relatedness clearly exists (for example, as in bone marrow suppression, fixed drug eruptions, or tardive dyskinesia).

†Rechallenge is when a drug suspected of causing an AE in a specific subject in the past is readministered to that subject. If the AE recurs upon exposure, this is termed a positive rechallenge. If the AE does not recur, this is termed a negative rechallenge.

7.5.1.3.3. Outcome

The investigator must assess the outcome of each AE as:

- Unresolved.
- Resolving.
- Resolved.
- Resolved with sequelae.
- Death.
- Unknown.
- Lost to follow-up.

Every effort should be made to determine the outcome of any AE that occurs at any point in the study.

7.5.1.3.4. Action

The PI must assess the action taken with regard to each AE as:

- None.
- Dose reduced.
- Use of concomitant medication.
- Discontinuation of the IMP.
- Withdrawal from the study.

7.5.1.4. Reporting of Serious Adverse Events

The Principal Investigator will review each SAE and evaluate the intensity and the causal relationship of the event to study drug. All SAEs will be recorded from signing of the ICF until the EOS Visit. Serious AEs occurring after the EOS Visit and coming to the attention of the Principal Investigator must be reported only if there is (in the opinion of the Principal Investigator) reasonable causal relationship with the study drug.

The Principal Investigator is responsible for providing notification to the Sponsor of any SAE, whether deemed IMP-related or not, that a subject reports during their participation in study within 24 hours of becoming aware of the event.

As a minimum requirement, the initial notification should provide the following information:

- Study number
- Subject number
- Sex
- Date of birth
- Name of Principal Investigator and full clinical site address
- Details of SAE
- Criterion for classification as ‘serious’

- Study drug name, or code if unblinded, and treatment start date
- Date of SAE onset
- Causality assessment (if sufficient information is available to make this classification)

The Sponsor will request clarification of omitted or discrepant information from the initial notification. The PI or an authorized delegate is responsible for faxing a FDA Form 3500A (MEDWATCH Form) to the study sponsor within 24 hours. The investigator shall maintain a copy of the MEDWATCH Form on file at the study site.

As a follow-up to the initial report, within the following 48 hours of awareness of the event, the PI shall provide further information, as applicable, on the unanticipated device event or the unanticipated problem in the form of a written narrative. This should include a copy of the completed Unanticipated Problem form, and any other diagnostic information that will assist the understanding of the event. Significant new information on ongoing unanticipated adverse device effects shall be provided promptly to the Sponsor.

Information on suspected unexpected serious adverse reactions (SUSARs) will be collected and reported to the regulatory authority. If the SUSAR is fatal or life-threatening, associated with the use of the IMP, and unexpected, the regulatory authority and the IRB will be notified within 7 calendar days after being made aware of the case. Additional follow-up (cause of death, autopsy report, hospital report) information should be reported within an additional 8 days (15 days in total). SUSARs which are not fatal and not life-threatening are to be reported within 15 days.

The Sponsor will notify the PI of relevant information about SUSARs that could adversely affect the safety of subjects in a timely fashion. Follow-up information may be submitted if necessary. The Sponsor will also provide annual safety reports for submission to the regulatory authority and the IRB responsible for the clinical study. These updates will include information on SUSARs and other relevant safety findings.

**SERIOUS ADVERSE EVENT REPORTING INSTRUCTIONS**<br>**Send the SAE Report Form and any supporting documentation**<br>**via email or fax within 24 hours of becoming aware of the event.**<br>[redacted]

7.5.1.5. Follow-up of Adverse Events

All AEs experienced by a subject, irrespective of the suspected causality, will be monitored until the event has resolved, until any abnormal laboratory values have returned to baseline or stabilized at a level acceptable to the Principal Investigator and Medical Monitor, until there is a satisfactory explanation for the changes observed or until the subject is lost to follow-up.

7.5.1.6. Pregnancy

The Sponsor has a responsibility to monitor the outcome of all pregnancies reported during the clinical study.

Pregnancy alone is not regarded as an AE unless there is a suspicion that the study drug may have interfered with the effectiveness of a contraceptive medication. Elective abortions without complications should not be regarded as AEs, unless they were therapeutic abortions (see below). Hospitalization for normal delivery of a healthy newborn should not be considered an SAE.

Each pregnancy must be reported by the Principal Investigator to the Sponsor within 2 days (after becoming aware of the pregnancy. The Principal Investigator must follow-up and document the course and the outcome of all pregnancies even if the subject was withdrawn from the clinical study or if the clinical study has finished.

All outcomes of pregnancy must be reported by the Principal Investigator to the Sponsor within 2 days after he/she has gained knowledge of the normal delivery or elective abortion.

Any SAE that occurs during pregnancy must be recorded on the SAE report form (e.g., maternal serious complications, therapeutic abortion, ectopic pregnancy, stillbirth, neonatal death, congenital anomaly, birth defect) and reported within 24 hours in accordance with the procedure for reporting SAEs.

7.5.2. Clinical Laboratory Assessments

Samples for clinical laboratory assessments will be collected at the time points detailed in the Schedule of Assessments (Table 3 and Table 4). Clinical laboratory tests will be performed by the laboratories mentioned in the List of Study Staff of this clinical study protocol. Samples will be collected in appropriate tubes and handled according to standard procedures of the applicable laboratory.

Clinical laboratory variables will be determined as outlined in Table 9.

Table 9 Clinical Laboratory Assessments

**Hematology**	
White blood cell (WBC) count	Mean corpuscular hemoglobin concentration (MCHC)
Red blood cell (RBC) count	Neutrophils (percentage and absolute count)
Reticulocytes	Lymphocytes (percentage and absolute count)
Hemoglobin (Hb)	Platelet count
Hematocrit (HCT)	Monocytes (percentage and absolute count)
Mean corpuscular volume (MCV)	Eosinophils (percentage and absolute count)
Mean corpuscular hemoglobin (MCH)	Basophils (percentage and absolute count)
**Coagulation**	
Activated partial thromboplastin time (aPTT)	International Normalized Ratio (INR)
D-dimer (Part B)	Prothrombin time (PT)
**Clinical Chemistry**	
Alanine aminotransferase (ALT)	Glucose
Albumin	Lactate Dehydrogenase
Alkaline phosphatase (ALP)	Phosphorus
Aspartate aminotransferase (AST)	Potassium
Blood urea nitrogen (BUN)	Sodium
Calcium	Total bilirubin
Chloride	Total protein
Cholesterol	Triglycerides
Creatinine	Troponin T
Creatine kinase (CK)	Troponin I
CRP	Uric acid
Gamma glutamyl transferase (GGT)	Follicle Stimulating Hormone (FSH)
**Viral Serology**	
Hepatitis B core antibody (anti-HBc)	Human immunodeficiency virus (HIV) (Types 1 and 2) antibodies
IgM antibody to anti-HBc (IgM anti-HBc)	
Hepatitis B surface antigen (HBsAg)	Hepatitis C virus antibody (anti-HCV)
	Tuberculin test
**Urine Drug Screening and Cotinine Test**	
Amphetamines	Cocaine
Barbiturates	Opiates
Benzodiazepines	Phencyclidine
Cannabinoids	Cotinine
Urinary creatinine (to exclude dilution effect)	
**Pregnancy Testing**	
Serum/urine human beta chorionic gonadotrophin (women of childbearing potential only)	

Abnormal laboratory results should be recorded as AEs (e.g., Principal Investigator judgment or outside the specific reference range).

Any value outside the normal range will be flagged for the attention of the PI or designee at the site. The PI or designee will indicate whether the value is of clinical significance. If the result of any test (or repeat test, if done) from the samples taken during the screening period is indicated as clinically significant, the subject will not be allowed into the study without permission of the Medical Monitor. Additional testing during the study may be done if medically indicated. If a clinically significant abnormality is found in the samples taken after dosing, during the study, and/or at the End-of-study Visit, it should be recorded as an AE and the subject will be followed until the test(s) has (have) normalized or stabilized, at the discretion of the Principal Investigator.

7.5.3. Vital Signs

Vital signs will be assessed at the time points detailed in the Schedule of Assessments (Table 3 and Table 4). The following vital signs will be measured:

- Blood pressure (systolic and diastolic [mmHg])
- Pulse (beats per minute [bpm])
- Oral body temperature (°C)
- Respiratory rate (breaths per minute)

Supine BP and pulse recordings will be made after the subject has been recumbent and at rest ≥5 minutes.

7.5.4. Standard 12-lead Electrocardiograms

Standard safety 12-lead ECGs will be performed at the time points detailed in the Schedule of Assessments (Table 3 and Table 4).

The 12-lead ECGs will be performed after the subject has been resting supine for ≥5 minutes. The ECG will include all 12 standard leads and a Lead II rhythm strip on the bottom of the tracing. The ECG will be recorded at a paper speed of 25 mm/sec. The following ECG parameters will be collected: PR interval, QRS interval, RR interval, QT interval and QTc interval (QTcB and QTcF).

All ECGs must be evaluated by a qualified physician for the presence of abnormalities.

7.5.5. Physical Examinations

Physical examinations will be performed at the time points detailed in the Schedule of Assessments (Table 3 and Table 4).

Full physical examination:

An assessment of general appearance and a review of systems (dermatologic, head, eyes, ears, nose, mouth/throat/neck, thyroid, lymph nodes, respiratory, cardiovascular, gastrointestinal, extremities, musculoskeletal, neurologic and psychiatric systems).

Anthropometric characteristics (height, weight, and BMI) will be recorded at the time points detailed in the Schedule of Assessments (Table 3 and Table 4).

7.5.6. Injection Site Monitoring

The investigator will evaluate the injection site at 1 hour, 6 hours, and 12 hours, 24 hours. 48 hours after every SC injection and will collect information on the presence of rash, swelling, bleeding, redness, induration and necrosis as demonstrated by the Local Injection Site Reaction Score (Appendix 5). If any severe injection related side effects occur, a dermatologist may be consulted to assess the degree of skin injury and to provide necessary therapy.

7.6. Pharmacokinetics Variables

7.6.1. Blood Sample Collection

Blood for the analysis of MRG-001 will be collected at the time points detailed in the Schedule of Assessments (Table 3 and Table 4).

Blood sample collection, processing and shipping details will be outlined in a separate laboratory manual. In brief, blood will be processed and plasma analyzed using a validated assay.

7.7. Pharmacodynamics Variables

Blood samples for the analysis of the following PD variables in both Part A and Part B will be investigated at the time points detailed in the Schedule of Assessments (Table 3 and Table 4).

- Stem cells panel: CD3, CD19/20, CD33, CD14, CD56, CD16) / CD45 / CD45RA/ CD38/ CD90/ CD133 / CD31 / CD34 / VEGFR2 / SSEA3 / Live-dead
- Immune cells panel: CD3 / CD4 / CD8 / CD19 / CD56 / CD16 / CD45RA / CCR7 / CD25 / CD127 / FOXP3 / ICOS / CTLA4 / Live-dead

7.8. Total Amount of Blood

Blood samples for the analysis of the PK and PD variables will be investigated at the time points detailed in the Schedule of Assessments Table 3 and Table 4. The approximate volumes of blood taken per subject for Part A and Part B are shown in Table 10 and Table 11.

[redacted] [redacted]

[redacted]	[redacted]	[redacted]	[redacted]
[redacted]			
[redacted]	[redacted]	[redacted]	[redacted]
[redacted]	[redacted]	[redacted]	[redacted]
[redacted]			
[redacted]	[redacted]	[redacted]	[redacted]
[redacted]			
[redacted]	[redacted]	[redacted]	[redacted]
[redacted]	[redacted]	[redacted]	[redacted]
[redacted]	[redacted]	[redacted]	[redacted]
[redacted]	[redacted]		[redacted]
[redacted]			[redacted]

[redacted] [redacted]

[redacted]	[redacted]	[redacted]	[redacted]
[redacted]			
[redacted]	[redacted]	[redacted]	[redacted]
[redacted]	[redacted]	[redacted]	[redacted]
[redacted]			
[redacted]	[redacted]	[redacted]	[redacted]
[redacted]			
[redacted]	[redacted]	[redacted]	[redacted]
[redacted]	[redacted]	[redacted]	[redacted]
[redacted]	[redacted]	[redacted]	[redacted]
[redacted]	[redacted]	[redacted]	[redacted]
[redacted]			[redacted]

8. STATISTICAL CONSIDERATIONS

Before database lock, a statistical analysis plan (SAP) will be issued as a separate document, providing detailed methods for the analyses outlined below. Any deviations from the planned analyses will be described and justified in the clinical study report (CSR).

8.1. Sample Size Considerations

For Part A formal sample size calculations were not performed, however with a total sample size of 18 healthy volunteers and a given incidence of a specific common adverse event of 1% in the general population the study would be able to detect an additional incidence of such adverse event caused by the use of the new drug of 11% with a power of 80%. Given the stage of development and the objective of Part A to determine the safety, pharmacokinetics (PK) and pharmacodynamics (PD) profiles of MRG-001 in asymptomatic SARS-CoV-2 infected subjects, 12 subjects were considered adequate to assess the safety and PK/PD profiles in this initial part of development.

[redacted]

[redacted]

[redacted] [redacted]

[redacted]	[redacted]	[redacted]	[redacted]
[redacted]	[redacted]	[redacted]	[redacted]
[redacted]	[redacted]	[redacted]	[redacted]
[redacted]	[redacted]	[redacted]	[redacted]
[redacted]	[redacted]	[redacted]	[redacted]
[redacted]	[redacted]	[redacted]	[redacted]
[redacted]	[redacted]	[redacted]	[redacted]

8.2. Study Population

8.2.1. Disposition of Subjects

The number and percentage of subjects screened, enrolled, randomized and completing the clinical study will be presented by treatment. Early discontinuations from treatment and study and reasons for discontinuation will be presented.

8.2.2. Protocol Deviations

Protocol deviations will be listed by subject.

8.2.3. Analysis Populations

Safety population: All randomized subjects who received at least one dose of study drug. Subjects will be included in the analysis according to the dose and study drug received.

The PK summaries and analyses will be based on the PK Population. If a subject is allocated the incorrect study treatment as per the study randomization list, subjects will be summarized and analyzed 'as treated' i.e. by actual treatment received. The subjects must complete the study without any major protocol deviation thought to interfere with the absorption, distribution, metabolism and excretion of MRG-001.

For the Part B the following analysis populations will be defined: Intent-to-Treat (ITT), modified ITT (mITT), per protocol (PP) and safety population:

ITT: All randomized subjects

mITT: All randomized subjects who received at least one dose of study drug and, for whom postbaseline assessment are available to assess the primary endpoint. Subjects will be included in the analysis according to the dose of study drug received. This population will be considered the primary analysis population for the efficacy endpoints and the other populations will be used as supportive analyses populations.

PP: All randomized subjects in the mITT population without major protocol deviation affecting the efficacy outcome.

Safety: all randomized subjects who received at least one dose of study drug. Subjects will be included in the analysis according to the actual dose and study drug received. Upon database release, protocol deviation and analysis population outputs will be produced and will be sent to Sponsor for review. Analysis population classifications will be discussed in Blinded Data Review

Meeting to discuss the outputs and to decide which subjects and/or subject data will be excluded from certain analyses. Decisions made regarding the exclusion of subjects and/or subject data from analyses will be made prior to unblinding and will be documented and approved by Sponsor.

8.3. General Considerations

Continuous data will be summarized by treatment group using descriptive statistics (number, mean, standard deviation [SD], minimum, median and maximum). Categorical data will be summarized by treatment group using frequency tables (number and percentage).

8.4. Protocol Deviations

All major protocol deviations will be listed by subject.

Protocol deviations will be handled in accordance with ICON's SOPs.

8.5. Subject Disposition

Subjects excluded from the different analysis sets and data excluded from the PK and PD analysis sets will be listed including the reason for exclusion. Subject disposition will be summarized and will include the following information: number of subjects randomized and dosed, number and percentage of subjects completing the study and the number and percentage of subjects who were withdrawn (including reasons for withdrawal). Disposition data will be presented based on all subjects randomized.

Subject discontinuations will be listed including the date of study exit, duration of treatment and reason for discontinuation. A listing of informed consent response will also be presented.

A randomization listing will be presented and include the following: each subject's/patient's randomization number, the subject's/patient's full enrolment number, the treatment to which the subject has been randomized and the actual treatment received and the location of the CU.

8.6. Demographic and Anthropometric Information and Baseline Characteristics

Demographic and anthropometric variables (age, sex, ethnicity, race, height, weight and BMI) will be listed by subject. Demographic characteristics (age, sex, ethnicity and race) and anthropometric characteristics (height, weight and BMI) will be summarized by treatment and for all subjects in the safety analysis set and the ITT/mITT population. The denominator for percentages will be the number of subjects in the respective analysis set for each treatment or for all subjects as applicable.

Medical history data will be listed by subject including visit, description of the disease/procedure, MedDRA SOC, MedDRA PT, start date, and stop date (or ongoing if applicable).

8.7. Prior and Concomitant Medication and Drug Administration

Prior medications are those that started and stopped prior to the first dose of IMP. Concomitant medications are those taken after first dosing (including medications that started prior to dosing and continued after).

Prior and concomitant medication will be listed by subject and will include the following information: reported name, preferred term, the route of administration, dose, frequency, start date/time, duration and indication.

Prior and concomitant medication will be coded according to the World Health Organization Drug Dictionary (WHO-DD) latest version.

Drug administration dates and times will be listed for each subject.

8.8. Exposure

A listing of drug administration will be created and will include the date and time of administration. When appropriate, a summary table of compliance will also be created.

8.9. Efficacy Analyses

[redacted].

The primary analysis population will be the mITT population

A SAP will provide details of the statistical methodology used for the analysis of Part A and Part B.

8.10. Safety Analyses

8.10.1. Adverse Events

Adverse event reporting will begin for each subject from the date the ICF is signed and will continue until the EOS Visit.

For the analysis, the concept of treatment-emergent AEs/SAEs will be applied. A treatment-emergent adverse event or serious adverse event (TEAE or TESAE) is defined as any event not present prior to the initiation of the drug treatment or any event already present that worsens in either intensity or frequency following exposure to the drug treatment. Summary tables with the number and percentage of subjects with TEAEs and TESAEs will be presented. All TEAE/TESAEs will be listed. The TEAEs will also be tabulated according to intensity and causality.

For the analysis the concept of treatment-emergent AEs/SAEs will be applied. A treatment-emergent adverse event or serious adverse event (TEAE or TESAE) is defined as any event not present prior to the initiation of the drug treatment or any event already present that worsens in either intensity or frequency following exposure to the drug treatment. Summary tables with the number and percentage of subjects with TEAEs and TESAEs will be presented.

8.10.2 Clinical Laboratory Tests

Individual data listings of laboratory results will be presented for each subject. Flags will be attached to values outside of the laboratory's reference limits along with the PI's assessment. Clinically significant laboratory test abnormalities that were considered AEs by the Principal Investigator will be presented in the AE listings.

Clinical laboratory tests (observed values) will be summarized descriptively in tabular format. In addition shift tables for change from baseline based on reference ranges will be provided.

8.10.3 Vital Signs

Individual data listings of vital signs (observed and change from baseline) will be presented for each subject. Individual clinically significant vital signs findings that were considered AEs by the Principal Investigator will be presented in the AE listings.

Observed values as well as change from baseline data will be summarized descriptively in tabular format.

8.10.4 Standard 12-lead Electrocardiogram

Standard 12-lead ECG data (observed and change from baseline) will be listed for each subject and time point. Observed values will be summarized descriptively in tabular format. Change from baseline will be summarized descriptively for QTc data. A categorical QTc analysis will also be performed.

8.10.5 Physical Examination

Abnormal physical examination findings will be listed.

8.11. Pharmacokinetics Analyses

The individual subject concentration-time data will be listed and displayed graphically on the linear and log scales. The concentration-time data will be summarized descriptively in tabular and graphical formats (linear and log scales). The non-compartmental PK parameters listed in Section 3.3.1.3 will be calculated using WinNonlin version 9.3 or later. The PK parameter data will be listed and summarized descriptively in tabular format.

8.12. Pharmacodynamics Analyses

Pre-dose measurements on Day 1 are considered baseline values for the PD variables.

Individual data listings of stem cell and immune cell activity and concentration will be presented by nominal time points and treatment.

Observed values as well as percentual change from baseline data will be summarized descriptively in tabular and figure format.

Further details on the PD analyses will be provided in the SAP.

8.13. Interim Analyses

No formal interim analysis is planned.

9 ETHICAL, LEGAL AND ADMINISTRATIVE ASPECTS

9.10 Data Quality Assurance

The Sponsor will conduct a study initiation visit to verify the qualifications of the PI, inspect the facilities and inform the Principal Investigator of responsibilities and procedures for ensuring adequate and correct documentation.

The PI must prepare and maintain adequate and accurate records of all observations and other data pertinent to the clinical study for each study participant. Frequent communication between the clinical site and the Sponsor is essential to ensure that the safety of the study is monitored adequately. The PI will make all appropriate safety assessments on an ongoing basis. The Medical Monitor may review safety information as it becomes available throughout the study.

All aspects of the study will be carefully monitored with respect to GCP and SOPs for compliance with applicable government regulations. The Study Monitor will be an authorized individual designated by the Sponsor. The Study Monitor will have access to all records necessary to ensure integrity of the data and will periodically review the progress of the study with the Principal Investigator.

Protocol deviations will be handled in accordance with ICON's SOP and IRB requirements.

9.11 Data collection and Access to Source Data/Documents

The Investigator will ensure the accuracy, completeness and timeliness of the data reported to the Sponsor. The Investigator or designee will cooperate with the Sponsor's representative(s) for the periodic review of study documents to ensure the accuracy and completeness of the data at each scheduled monitoring visit.

The Investigator will allow Sponsor representatives, contract designees, authorized regulatory authority inspectors and the IEC to have direct access to all records pertaining to the study.

All data should be recorded, handled and stored in a way that allows its accurate reporting, interpretation and verification.

9.12 Archiving Study Documents

All source documents generated in connection with the study will be retained in the limited access file storage area, respecting the privacy and confidentiality of all records that could identify the

subjects. Direct access is allowed only for authorised people for monitoring and auditing purposes. Source documents will be handled, stored and archived according to in-house procedures.

It is the PI's responsibility to retain study essential documents for at least 2 years after the last approval of a marketing application in their country and until there are no pending or contemplated marketing applications in their country or at least 2 years have elapsed since the formal discontinuation of clinical development of the investigational product. These documents should be retained for a longer period if required by an agreement with the Sponsor. In such an instance, it is the responsibility of the Sponsor to inform the investigator/institution as to when these documents no longer need to be retained.

9.13 Good Clinical Practice

The procedures set out in this clinical study protocol are designed to ensure that the Sponsor and the Principal Investigator abide by the principles of the ICH guidelines on GCP. The clinical study also will be carried out in keeping with national and local legal requirements in accordance with United States investigational new drug [IND] regulations [21 CFR 56]).

9.14 Informed Consent

Eligible subjects may only be included in the study after providing IRB approved informed consent.

Informed consent must be obtained from the subject before conducting any study-specific procedure.

As part of the informed consent procedure, the PI or designee must explain orally and in writing the nature, duration and purpose of the study and the action of the drug in such a manner that the subject is aware of the potential risks, inconveniences or AEs that may occur. The subject should be informed that he/she is free to withdraw from the study at any time. Subjects will receive all information that is required by federal regulations and ICH guidelines. The ICF must be signed and dated; one copy will be handed to the subject, and the Principal Investigator will retain a copy as part of the clinical study records. The PI will not undertake any investigation specifically required for the clinical study until written consent has been obtained. The terms of the consent and when it was obtained must be documented in the subject source documents.

The Sponsor will review the investigator-proposed ICF to ensure it complies with the ICH GCP guideline (including the ethical principles that have their origins in the Declaration of Helsinki)

and regulatory requirements and is considered appropriate for this study. The Principal Investigator or designee will provide the Sponsor with a copy of the IRB approved ICF prior to the start of the study.

If a protocol amendment is required, then the ICF may need to be revised to reflect the changes to the protocol. If the ICF is revised, it must be reviewed and approved by the responsible IRB and signed by all subjects subsequently enrolled in the clinical study, as well as those currently enrolled in the clinical study as applicable.

9.15 Insurance and Compensation for Injury

The Sponsor has covered this clinical study by means of an insurance of the clinical study according to national requirements.

In the event of a subject suffering any bodily injury caused directly by his/her participation in the study, compensation will be paid to the subject according to rules set forth in the Chubb Life Sciences Products- Completed Operations Liability Policy Claims- Made Coverage.

Details are provided in the ICF.

9.16 Protocol Approval and Amendment(s)

Before the start of the clinical study, the clinical study protocol and other relevant documents will be approved by the IRB, in accordance with local legal requirements. The Sponsor must ensure that all ethical and legal requirements have been met before the first subject is enrolled in the clinical study.

This protocol is to be followed exactly. To alter the protocol, amendments must be written, which must be released by the responsible staff and receive IRB approval prior to implementation (as appropriate).

Administrative changes may be made without the need for a formal amendment but will also be mentioned in the integrated clinical study report. All amendments will be distributed to all study protocol recipients, with appropriate instructions.

9.17 Confidentiality Data Protection

Information about study subjects will be kept confidential and managed according to the requirements of the Health Insurance Portability and Accountability Act of 1996 (HIPAA). Those regulations require a signed subject authorization informing the subject of the following:

- What protected health information (PHI) will be collected from subjects in this study.
- Who will have access to that information and why.
- Who will use or disclose that information.
- The rights of a research subject to revoke their authorization for use of their PHI.

All clinical study findings and documents will be regarded as confidential. Study documents (protocols, IBs and other material) will be stored appropriately to ensure their confidentiality. The Principal Investigator and members of his/her research team (including the IRB/IEC) must not disclose such information without prior written approval from the Sponsor, except to the extent necessary to obtain informed consent from subjects who wish to participate in the trial or to comply with regulatory requirements.

In the event that a subject revokes authorization to collect or use PHI, the investigator, by regulation, retains the ability to use all information collected prior to the revocation of subject authorization. For subjects that have revoked authorization to collect or use PHI, attempts should be made to obtain permission to collect at least vital status (i.e. that the subject is alive) at the end of their scheduled study period.

The anonymity of participating subjects must be maintained. Subjects will be specified on study documents by their subject number, initial or birth date, not by name. Documents that identify the subject (e.g., the signed ICF) must be maintained in confidence by the PI.

[redacted] [redacted]

[redacted]

[redacted]

10. REFERENCE LIST

Bonow RO, Fonarow GC, O'Gara PT, Yancy CW. Association of Coronavirus Disease 2019 (COVID-19) with myocardial injury and mortality. JAMA Cardiol. 2020;5(7):751–753. doi:10.1001/jamacardio.2020.1105.

Brandon Michael Henry, Gaurav Aggarwal, Johnny Wong, Stefanie Benoit, Jens Vikse, Mario Plebani, Giuseppe Lippi. Lactate dehydrogenase levels predict coronavirus disease 2019 (COVID-19) severity and mortality: A pooled analysis. Am J Emerg Med. 2020;38(9):1722–1726.

Cameron AM, Wesson RN, Ahmadi AR, Singer AL, Hu X, Okabayashi T, Wang Y, Shigoka M, Fu Y, Gao W, Raccusen LC, Montgomery RA, Williams GM, Sun Z. Chimeric Allografts induced by short-term treatment with stem cell mobilizing agents result in long-term kidney transplant survival without immunosuppression: II, Study in Miniature Swine. Am J Transplant. 2016;16 (7):2066-76.

Chan KW, Wong VT, Tang SCW. COVID-19: An Update on the Epidemiological, Clinical, Preventive and Therapeutic Evidence and Guidelines of Integrative Chinese-Western Medicine for the Management of 2019 Novel Coronavirus Disease. Am J Chin Med. 2020;48(3):737-762. doi:10.1142/S0192415X20500378.

Chen, G., Wu, D., Guo, W., Cao, Y., Huang, D., Wang, H., ... & Zhang, X. (2020). Clinical and immunological features of severe and moderate coronavirus disease 2019. The Journal of clinical investigation, 130(5).

Coperchini,F, Chiovato L, Croce L, Magri F, Rotondi M. The cytokine storm in COVID-19: An overview of the involvement of the chemokine/chemokine-receptor system. Cytokine Growth Factor Rev.2020; 53: 25.

D'Alessio F.R. Mouse Models of Acute Lung Injury and ARDS. In: Alper S., Janssen W. (eds) Lung innate immunity and inflammation. methods in molecular biology, vol 1809 2018. Humana Press, New York, NY. https://doi.org/10.1007/978-1-4939-8570-8_22.

Diao B, Wang C, Tan Y, et al. Reduction and functional exhaustion of t cells in patients with coronavirus disease 2019 (COVID-19). Front Immunol. 2020;11:827. Published 2020 May 1. doi:10.3389/fimmu.2020.00827.

Ding Y., He L., Zhang Q. Organ distribution of severe acute respiratory syndrome (SARS) associated coronavirus (SARS-CoV) in SARS patients: implications for pathogenesis and virus transmission pathways. J Pathol. 2004;203:622.

Gabarre, P., Dumas, G., Dupont, T., Darmon, M., Azoulay, E., & Zafrani, L. (2020). Acute kidney injury in critically ill patients with COVID-19. Intensive Care Medicine, 1-10.

George PM, Wells AU, Jenkins RG. Pulmonary fibrosis and COVID-19: the potential role for antifibrotic therapy [published online ahead of print, 2020 May 15]. Lancet Respir Med. 2020;8(8):807-815. doi:10.1016/S2213-2600(20)30225-3.

Guan W.J., Ni Z.Y., Hu Y. Clinical characteristics of coronavirus disease 2019 in China [published online ahead of print, 2020 Feb 28] N Engl J Med. 2020 doi: 10.1056/NEJMoa2002032. NEJMoa2002032.

Hamming I., Timens W., Bulthuis M.L., Lely A.T., Navis G., van Goor H. Tissue distribution of ACE2 protein, the functional receptor for SARS coronavirus. A first step in understanding SARS pathogenesis. J Pathol. 2004;203:631.

Hendrix CW et al. Safety, Pharmacokinetics, and Antiviral activity of amd3100, a selective cxcr4 receptor inhibitor, in hiv-1 infection. J Acquir Immune Defic Syndr 2004;37(2):1253-62.

Henry, B. M., Aggarwal, G., Wong, J., Benoit, S., Vikse, J., Plebani, M., & Lippi, G. (2020). Lactate dehydrogenase levels predict coronavirus disease 2019 (COVID-19) severity and mortality: A pooled analysis. The American Journal of Emergency Medicine.

Hu X, Okabayashi T, Cameron AM, Wang Y, Hisada M, Li J, Raccusen LC, Zheng Q, Montgomery RA, Williams GM, Sun Z. Chimeric Allografts induced by short-term treatment with stem cell-mobilizing agents result in long-term kidney transplant survival without immunosuppression: a study in rats. Am J Transplant. 2016;16(7):2055-65.

Huang C., Wang Y., Li X. Clinical features of patients infected with 2019 novel coronavirus in Wuhan, China [published correction appears in Lancet. 2020 Jan 30; Lancet. 2020;395:497

Iwasaki K, Ahmadi A, Qi L, Chen M, Wang W, Cameron A, Burdick J, Sun Z. Pharmacological mobilization of endogenous stem cells prevents postsurgical intra-abdominal adhesion in rats. Sci Reports. Sci Rep. 2019;9(1):7149.

Lamers MM, Beumer J, van der Vaart J, et al. SARS-CoV-2 productively infects human gut enterocytes. Science. 2020;369(6499):50-54. doi:10.1126/science.abc1669.

Liao, Y. C. et al. IL-19 induces production of IL-6 and TNF-alpha and results in cell apoptosis through TNF-alpha. J. Immunol. 2020;169:4288.

Li Y, Chen M, Cao H, Zhu Y, Zheng J, Zhou H. Extraordinary GU-rich single-strand RNA identified from SARS coronavirus contributes an excessive innate immune response. Microbes Infect. 2013;15(2):88-95. doi:10.1016/j.micinf.2012.10.008

Lu, R., Zhao, X., Li, J., Niu, P., Yang, B., Wu, H., ... & Bi, Y. (2020). Genomic characterisation and epidemiology of 2019 novel coronavirus: implications for virus origins and receptor binding. The Lancet, 395(10224), 565-574.

Lurie, N., Saville, M., Hatchett, R., and Halton, J. Developing covid-19 vaccines at pandemic speed. N Engl J Med 2020;382:1969–1973.

National Institutes for Health. COVID-19 Treatment Guidelines Panel. Coronavirus Disease 2019 (COVID-19) Treatment Guidelines. Available at https://www.covid19treatmentguidelines.nih.gov/. Accessed 2020.

Okabayashi T, Cameron AM, Hisada M, Montgomery RA, Williams GM, Sun Z. Mobilization of host stem cells enables long-term liver transplant acceptance in a strongly rejecting rat strain combination. Am J Transplant. 2011 Oct;11(10):2046-56. doi: 10.1111/j.1600-6143.2011.03698.x. Epub 2011 Aug 30. PMID: 21883903; PMCID: PMC3190303.

Parasa S, Reddy N, Faigel DO, Repici A, Emura F, Sharma P. Global Impact of the COVID-19 pandemic on endoscopy: an international survey of 252 centers from 55 countries [published online ahead of print, 2020 Jun 11]. Gastroenterology. 2020;S0016-5085(20)34761-2. doi:10.1053/j.gastro.2020.06.009.

Peiffer BJ, Qi L, Ahmadi AR, Wang Y, Guo Z, Peng H, Sun Z, Liu JO. Activation of BMP signaling by fkbp12 ligands synergizes with inhibition of cxcr4 to accelerate wound healing. cell Chem Biol. 2019;26(5):652-661.

Plerixafor prescribing information. https://www.accessdata.fda.gov/drugsatfda_docs/label/2017/022311s018lbl.pdf

Pons, S., Fodil, S., Azoulay, E. *et al.* The vascular endothelium: the cornerstone of organ dysfunction in severe SARS-CoV-2 infection. Crit Care 24, 353 (2020). https://doi.org/10.1186/s13054-020-03062-7.

Prograf Label, 2019 https://www.accessdata.fda.gov/drugsatfda_docs/label/2012/050709s031lbl.pdf.

Qi L, Ahmadi AR, Huang J, Chen M, Pan B, Kuwabara H, Iwasaki K, Wang W, Wesson R, Cameron AM, Cui S, Burdick J, Sun Z. Major Improvement in Wound Healing Through Pharmacologic Mobilization of Stem Cells in Severely Diabetic Rats. Diabetes. 2020 Jan 23. pii: db190907. doi: 10.2337/db19-090.

Lin Q, Wesson R, Maeda H, Wang Y, Cui Z, Liu O, Gao B, Williams G, and Sun Z. Pharmacological Mobilization Of Autologous Stem Cells Significantly Promotes Skin Regeneration After Full Thickness Excision: The Synergistic Activity Of AMD3100 And Tacrolimus. Journal of Investigative Dermatology 2014 Sep; 134(9):2458-68.

Schiff J, Cole E, and Cantarovich M. Therapeutic Monitoring of Calcineurin Inhibitors for the Nephrologist. CJASN. 2007, 2 (2) 374-384; DOI: https://doi.org/10.2215/CJN.03791106.

Spiekerkoetter E, Sung YK, Sudheendra D, et al. Low-Dose FK506 (Tacrolimus) in End-Stage Pulmonary Arterial Hypertension. Am J Respir Crit Care Med. 2015;192(2):254-257. doi:10.1164/rccm.201411-2061LE.

Stewart DA, Smith C, MacFarland R, Calandra G. Pharmacokinetics and pharmacodynamics of plerixafor in patients with non-Hodgkin lymphoma and multiple myeloma. Biology of Blood and Marrow Transplantation. 2009 Jan 1;15(1):39-46.

Sun, Z Patent US20200038381A1 - Methods of treating inflammatory bowel disease with amd3100 and tacrolimus - https://patents.google.com/patent/US20200038381A1/en

Tan L, Wang Q, Zhang D, Ding J, Huang Q, Tang Y-Q, Wang Q, Miao H. Lymphopenia predicts disease severity of COVID- 19: a descriptive and predictive study. Signal Transduct Target Ther. 2020;5:33.

Wang D, Hu B, Hu C, et al. Clinical Characteristics of 138 Hospitalized Patients With 2019 Novel Coronavirus-Infected Pneumonia in Wuhan, China. JAMA. 2020;323(11):1061-1069. doi:10.1001/jama.2020.1585.

WHO R&D Blueprint novel Coronavirus COVID-19 therapeutic trial synopsis. Available at https://www.who.int/blueprint/priority-diseases/key-action/COVID-19_Treatment_Trial_Design_Master_Protocol_synopsis_Final_18022020.pdf. Accessed 2020.

Wu Z, McGoogan JM: Characteristics of and important lessons from the coronavirus disease 2019 (COVID-19) outbreak in China. JAMA. 2020;323:1239.

Zhai R, Wang Y, Qi L, Song G, Williams G, Gao B, Burdick J, Sun Z. Pharmacological Mobilization of Endogenous Bone Marrow Stem Cells Promotes Liver Regeneration after Extensive Liver Resection in Rats. Sci Rep. 2018;8(1): 3587.

Zhang R, Li Y, Zhang AL, Wang Y, Molina MJ. Identifying airborne transmission as the dominant route for the spread of COVID-19. Proc Natl Acad Sci *U S A*. 2020;117(26):14857-14863. doi:10.1073/pnas.2009637117.

Zhao Q, Meng M, Kumar R, et al. Lymphopenia is associated with severe coronavirus disease 2019 (COVID-19) infections: A systemic review and meta-analysis. Int J Infect Dis. 2020;96:131-135. doi:10.1016/j.ijid.2020.04.086.

Zhou, P., Yang, X. L., Wang, X. G., Hu, B., Zhang, L., Zhang, W., ... & Chen, H. D. (2020). A pneumonia outbreak associated with a new coronavirus of probable bat origin. nature, 579(7798), 270-273.

Zsuzsanna Varga, Andreas J Flammer, Peter Steiger, Martina Haberecker, Rea Andermatt, Annelies S Zinkernagel, Mandeep R Mehra, Reto A Schuepbach, Frank Ruschitzka, Holger Moch. Endothelial cell infection and endotheliitis in COVID-19. Lancet. 2020;395(10234):1417–1418.

11 APPENDICES

[redacted] [redacted]

[redacted]

[redacted]

[redacted]

[redacted]

[redacted]

[redacted]

[redacted]

- [redacted]
- [redacted]
- [redacted]
- [redacted]
- [redacted]

[redacted]

Source: COVID-19 Treatment Guidelines Panel. Coronavirus Disease 2019 (COVID-19) Treatment Guidelines. National Institutes of Health. Available at https://www.covid19treatmentguidelines.nih.gov/. Accessed 2020.

Appendix 5 Local Injection Site Reaction Score

Parameter	Extent	Score
**Redness**	No	0
	Slight	1
	Severe	2
**Size of redness**	0.00–0.50 cm	0
	0.60–2.00 cm	1
	> 2.00 cm	2
**Swelling**	No	0
	Slight	1
	Severe	2
**Pain during palpation**	No	0
	Slight	1
	Severe	2
**Necrosis/ulceration**	No	0
	Slight	1
	Severe	2
**Induration**	No	0
	Firm	1
	Hard	2
**Maximum score**		**12**

Injection sites will be monitored for presence or absence of reactions including redness, size of redness, swelling, pain during palpation, necrosis/ulceration and induration. This clinical scoring system provides an objective and reliable method to not only monitor for injection site related side effects but is also able to monitor progression or regression of registered injection site problems. Injection sites will be scored with this scoring tool every 1, 6, 12 and 24 hours. This scoring system will also aid in objectively determining the frequency and degree of clinical signs between both the MRG-001 and placebo group.
